# Optimized mechano-fluidic metamaterials inspired by deep-sea sponges

**DOI:** 10.1038/s41467-026-72612-4

**Published:** 2026-05-05

**Authors:** Timon Meier, Sergey Litvinov, Runxuan Li, Brian W. Blankenship, Andrew Kokubun, David Hahn, Stefanos Mavrikos, Zacharias Vangelatos, M. Erden Yildizdag, Simo A. Mäkiharju, Xiaoyu Zheng, Petros Koumoutsakos, Costas P. Grigoropoulos

**Affiliations:** 1 Laser Thermal Laboratory, Department of Mechanical Engineering, University of California, Berkeley, CA USA; 2 School of Engineering and Applied Sciences, Harvard University, Cambridge, MA USA; 3 FLOW Lab, Department of Mechanical Engineering, University of California, Berkeley, CA USA; 4 Department of Materials Science and Engineering, University of California, Berkeley, CA USA; 5 Faculty of Naval Architecture and Ocean Engineering, Istanbul Technical University, Istanbul, Türkiye

**Keywords:** Mechanical engineering, Mechanical properties, Engineering

## Abstract

Multifunctional materials that balance mechanical resilience and fluid dynamic efficiency are critical in engineering applications, yet their synergistic optimization remains challenging due to inherent trade-offs, computational expense, and high-dimensional design spaces. Inspired by the skeleton of the deep-sea sponge *Euplectella aspergillum*, this work presents an automated framework integrating Finite Element Analysis for mechanics, Computational Fluid Dynamics for flow behavior, and multi-objective Bayesian optimization. Leveraging high-performance computing, the framework efficiently explores complex design spaces to identify Pareto-optimal solutions. Optimized lattices achieve an average 140% increase in critical buckling load across a range of volume fractions relative to baseline designs, while simultaneously reducing drag, lift, and vortex shedding at porosities as low as 5%. We fabricate selected designs via stereolithography and validate them through compression experiments and particle image velocimetry, showing agreement with simulations. By jointly optimizing mechanics and fluidics, this work establishes a scalable methodology for designing lightweight, high-performance architected materials.

## Abstract (web summary)

Inspired by deep-sea sponge skeletons, this study introduces an automated design framework for architected materials that simultaneously improve mechanical strength and fluid flow efficiency.

## Introduction

Nature, through millions of years of evolutionary processes, has developed a wide range of structures and materials that show efficiency, adaptability, and multifunctionality. This natural optimization, driven by survival and reproduction, has led to biological designs that often surpass both intuitive and engineered solutions. As our understanding of these systems deepens, so does our ability to draw inspiration from them, fueling the field of biomimetic engineering^1,2^. From the self-cleaning properties of lotus leaves^3^ to the impact resistance of mollusk shells^4^, the adhesive capabilities of gecko feet^5^, and the color-changing abilities of chameleon skin^6^, nature offers a vast repository of design principles.

Load-bearing biological structures are especially compelling for their ability to achieve mechanical strength with minimal material. Examples for lightweight yet strong structures include the trabecular bone, with its optimized lattice structure^7^, and the honeycomb architecture^8^ found in beehives. Marine organisms, which face unique challenges in their aquatic environments, offer additional inspiration. The deep-sea glass sponge, *Euplectella aspergillum (E.a.)*^9,10^, commonly known as Venus’ flower basket, is a notable example of structural efficiency and multifunctionality^11–15^. This organism has evolved a skeletal system that combines lightweight design with high mechanical strength^16–20^ and distinctive fluid dynamic interactions^21–26^. Over millions of years, the sponge’s structure has evolved to withstand the extreme conditions of its deep-sea habitat, such as high pressures, limited light, cold temperatures, and persistent exposure to fluid flows. The skeletal structure of *E.a*. consists of a lattice-like arrangement of silica spicules, forming a cylindrical structure with a hierarchical organization spanning multiple length scales^11^.

Since the early 2000s, studies have extensively explored the mechanical and fluidic aspects of this architecture, establishing *E.a*. as a long-standing framework for biomimetic benchmarks and optimization. Weaver et al.^12^ demonstrated the structural integrity of its six-level hierarchical design, ranging from nanometers to centimeters, emphasizing its efficiency in material use and mechanical stability. Monn et al.^13,18^ further showed that the individual sponge spicules exhibit high flexibility and optimal strength.

At the lattice scale, several works have explored the mechanical efficiency of sponge-inspired architectures and formulated explicit optimization problems focused on structural performance. Fernandes et al.^16^ employed evolutionary optimization (CMA-ES) to explore 2.5D cubic lattice design spaces and showed that the sponge’s characteristic checkerboard geometry with double-diagonal reinforcement approaches the theoretical optimum for buckling resistance, achieving ~20% higher effective buckling stress compared to conventional diagonally reinforced lattices. Li and Sun^27^ extended this analysis using multi-objective optimization (NSGA-II), identifying alternative sponge-inspired configurations that improved stiffness and strength while reducing redundant material. Fully three-dimensional implementations were investigated by Li et al.^28^, who demonstrated that cylindrical sponge-inspired lattices outperform hollow honeycomb structures in compressive strength-to-weight ratio. Vangelatos et al.^19^ analyzed the nonlinear buckling behavior of sponge-inspired lattices and designed an optimized structure via topology optimization, achieving higher load-carrying capacity with reduced volume compared to a baseline geometry. Sharma and Hiremath^29^ extended sponge-inspired concepts to crashworthiness applications, showing through parametric studies that bioinspired thin-walled tubes exhibit significantly higher specific energy absorption at lower peak crushing forces than other bionic tube designs. Chen et al.^20^ performed parametric mechanical simulations and showed that sponge-like double-diagonal lattices tend to maximize torsional rigidity while exhibiting weak sensitivity to diagonal spacing, enabling independent control of porosity relevant for fluid transport. In addition, Du et al.^30^ introduced machine-learning-assisted surrogate modeling to optimize simplified sponge-like lattices, achieving improvements of roughly 40% buckling resistance relative to the biological baseline.

In parallel, substantial effort has been devoted to understanding the hydrodynamic role of the *E.a*. skeleton. Chen et al.^20^ studied the influence of the helical ridge system spiraling along the cylindrical lattice and showed that these ridges enhance radial stiffness while affecting fluid transport. Using Computational Fluid Dynamics (CFD) on sponge-inspired porous, thin-plate representations, they reported improved permeability under laminar conditions (Re ≈ 43), though such models do not capture the full three-dimensional wake dynamics around a cylindrical geometry. Falcucci et al.^21,24^ carried out high-performance computing (HPC) flow simulations on a complete skeletal model of *E.a*., showing that the sponge skeleton reduces hydrodynamic stress and drag forces by up to 49% at Re ≈ 2000 compared to solid cylinders. In related work, Fernandes et al.^22^ analyzed the flow–structure interaction of ridge-reinforced cylindrical models and demonstrated that the sponge’s ridge system suppresses von Kármán vortex shedding^31,32^, thereby mitigating resonance-induced vibrations and reducing lift-force oscillations across flow regimes. While these ridges provide only a limited contribution to mechanical resistance under compression, they were identified as a key geometric feature governing vortex-shedding control in bioinspired cylindrical structures.

Taken together, prior work has firmly established the outstanding mechanical efficiency and favorable hydrodynamic characteristics of *E.a*.-inspired architectures, making them attractive templates for engineered systems where structural resilience and fluid control are equally important, such as offshore structures, aerospace components, filtration devices, and biomedical scaffolds. However, existing studies have predominantly treated mechanical and fluid-dynamic performance as separate objectives. Fluidic behavior has not been explicitly optimized, nor has it been systematically coupled with mechanical performance within a unified, large-scale multi-objective design framework. Even studies that addressed both physics typically considered them sequentially or in isolation, with CFD evaluations limited to a small number of representative geometries rather than embedded within automated optimization loops exploring extensive design spaces. Moreover, experimental validation has largely focused on mechanical metrics, while fluid-dynamic performance has rarely been measured or quantitatively compared with CFD predictions. Consequently, the coupled trade-offs that ultimately govern multifunctional performance in sponge-inspired architectures remain insufficiently characterized. Addressing these challenges requires an integrated framework capable of balancing competing objectives, which is increasingly important given the demand for lightweight structures and efficient fluid management in engineering applications.

Recent advances in computational modeling, simulation techniques, multi-objective optimization, and HPC now offer the tools to address this complexity, enabling the development of designs that integrate and optimally balance both mechanical and fluidic performance. This work presents an automated framework that combines high-fidelity CFD and Finite Element Analysis (FEA) simulations within a multi-objective Bayesian optimization (MOBO)^33,34^ scheme. While traditional optimization algorithms, like NSGA-II^35^ and MOEA/D^36^ have been widely used for multi-objective optimization, they struggle to handle the computational expense of high-fidelity simulations. In contrast, MOBO enables efficient optimization of costly black-box functions with a limited number of evaluations^37,38^ and can be parallelized on high-performance computing resources. Here, MOBO relies on probabilistic Gaussian-process surrogate models for each objective, allowing uncertainty-aware sampling and sample-efficient exploration of the design space.

Using these capabilities, our framework efficiently explores complex design spaces to identify Pareto-optimal solutions inspired by the *E.a*. skeleton, balancing mechanical performance, defined as the critical buckling load normalized by volume, and hydrodynamic performance, quantified by drag force, lift force, and vortex-shedding behavior. Detailed formulations of both cost functions are provided in the “Methods” section (Eqs (1) and (2)). The study combines high-fidelity computational modeling with 3D fabrication via high-precision stereolithography (SLA) and experimental validation using mechanical compression tests and stereo particle image velocimetry (SPIV). By integrating simulation-driven multi-objective optimization with experimental verification, we establish a general methodology for translating bioinspired principles into multifunctional architected designs. This research not only advances the understanding of *E.a*. structural principles but more importantly demonstrates their relevance in engineering, where lightweight, resilient, and efficient multifunctional designs are critical.

## Results

### Design framework

**Fig. 1:**
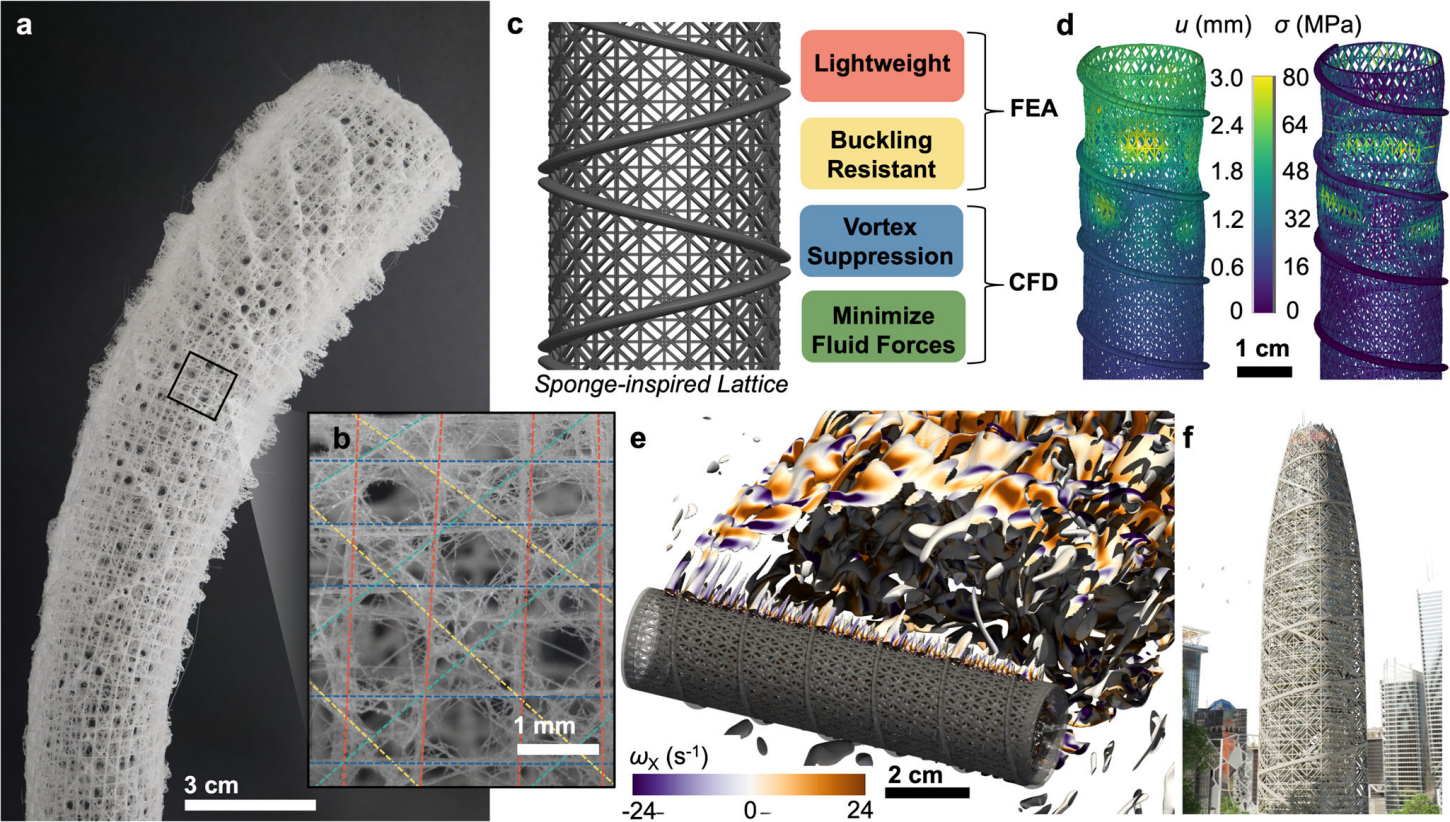
Biological inspiration, geometric abstraction, and multifunctional performance of sponge-inspired lattice architectures. **a** Photograph of the deep-sea sponge *Euplectella aspergillum*, whose skeletal architecture exhibits hierarchical organization, diagonal reinforcement, and helical ridges. **b** Magnified view of the sponge skeleton highlighting characteristic geometric motifs, including longitudinal and circumferential beams, diagonal reinforcements, and external ridges. **c** Schematic of a parametric lattice model inspired by the lightweight sponge architecture, forming the basis of the computational design and optimization framework. The engineered geometry preserves key structural features while enabling evaluation of buckling resistance using finite element analysis and vortex suppression and fluid dynamic forces through computational fluid dynamics. **d** Finite element analysis of an example lattice under axial compression, showing displacement (*u*, mm; left) and equivalent (von Mises) stress (σ, MPa; right). Colorbars indicate field magnitude and units. A scale bar is included to indicate the physical size of the structure. These simulations are used to quantify stiffness and critical buckling load. **e** Computational fluid dynamics simulation of incompressible flow through the lattice (Navier–Stokes with Brinkman penalization), illustrating three-dimensional flow structures and wake behavior used to quantify fluid dynamic forces. The background color represents the vorticity magnitude (*ω*_x_, s^−^¹), highlighting vortex dynamics around the porous geometry (structure height: 100 mm). **f** Conceptual application illustrating how optimized sponge-inspired lattice architectures can be integrated into lightweight, flow-interacting engineering or architectural systems.

We translate biological insights into engineered multifunctional designs, developing a computational framework inspired by the hierarchical lattice structure of *E.a*. As illustrated in Fig. 1, the sponge skeleton features a checkerboard-like grid of longitudinal and circumferential beams, diagonal reinforcements, and helical ridges curling over the tubular structure. Together, these features contribute to lightweight construction, improved buckling resistance, and passive vortex suppression.

Building on the structural insights from *E.a*., we developed a parameterized design space that captures the sponge’s key architectural features in an idealized cylindrical lattice model. The abstraction incorporates the square lattice of longitudinal and circumferential beams, reinforced by double diagonals and external helical ridges, and serves as the foundation for computational modeling and optimization. The cylindrical lattice is defined with constant length *L* and diameter *D*. The multiple spicule fibers within the sponge are represented as beams with rectangular cross sections, described by the design parameters width *W* and height *H*. Additional design parameters include the number of vertical beams *N*_V_, the number of circumferential beams *N*_C_, the radius of the semi-ellipsoidal cross-section for the helical ridges *R*_H_, the number of loops in the helical ridges *N*_L_, and the counts for clockwise (CW) and counterclockwise (CCW) helical ridges *N*_CW,_ *N*_CCW_. Each parameter was chosen to enable precise control over the structural elements, with boundaries set by the sponge’s skeletal feature sizes and stability constraints for the computational simulations. An overview of all design parameters and their ranges is shown in Table 1.

**Table 1:** Geometric design parameters and ranges used in the optimization

Parameter	Description	Parameter range
*L*	Length of cylindrical lattice	100 mm
*D*	Diameter of cylindrical lattice	22 mm
*W*	Width of cross-section	0.15–0.65 mm
*H*	Height of cross-section	0.15–0.65 mm
*N*_v_	Number of vertical beams	20–50
*N*_c_	Number of circumferential beams	30–74
*R*_H_	Radius of the semi-ellipsoid of helical ridges	0.6–1.6 mm
*N*_L_	Number of loops for helical ridges	1–6
*N*_CW_	Number of clockwise helical ridges	1–3
*N*_CCW_	Number of counterclockwise helical ridges	1–3

The parameterized cylindrical lattice is defined by geometric variables controlling beam dimensions, lattice topology, and helical ridge architecture. The lattice length (*L* = 100 mm) and diameter (*D* = 22 mm) are held constant, while the remaining parameters are varied within the ranges shown to explore the design space. Beam cross sections are defined by width (*W*) and height (*H*), and lattice topology is controlled by the number of vertical (*N*_V_) and circumferential (*N*_C_) beams. Helical ridge geometry is described by the semi-ellipsoidal radius (*R*_H_), number of loops (*N*_L_), and the number of clockwise (*N*_CW_) and counterclockwise (*N*_CCW_) ridges. Parameter bounds are chosen based on characteristic feature sizes of the biological template and constraints for geometric feasibility and numerical stability in simulations.

Geometries were generated using custom Python scripts, as detailed in Supplementary Methods and Software, and evaluated with high-fidelity FEA and CFD simulations to capture mechanical and fluidic performance. FEA simulations, implemented through the ANSYS^®^ Python environment (PyAnsys), were used to study how variations in geometry affect the mechanical behavior of the lattice. In parallel, CFD simulations were performed using the incompressible Navier–Stokes solver in Basilisk^39^ with Brinkman penalization^40^ and adaptive mesh refinement (AMR) to resolve fluid forces, wake dynamics, and vortex shedding around the lattice structures. During the MOBO stage, moderate-resolution, flow-resolved AMR settings were employed to efficiently explore the design space across hundreds of candidate geometries. For all final Pareto-optimal designs, higher-resolution simulations were subsequently performed involving grids at a resolution that enabled Direct Numerical Simulations (DNS). The validity and rationale of this two-fidelity simulation strategy are detailed in the Supplementary Methods. We selected a ${\mathrm{Re}}={UD}/\nu=2100$ for optimization as it corresponds to typical deep-sea flow conditions^21^.

Building on our previous inverse design frameworks^41,42^ for tailoring mechanical properties of architected materials, we apply MOBO to explore the design space and identify optimal trade-offs between mechanical and fluid dynamic properties. In contrast to our prior studies, which employed either NSGA-II genetic algorithms requiring large numbers of evaluations or single-objective Bayesian optimization with limited scalability, the present framework enables fully automated multi-objective optimization integrating FEA and CFD simulations with batch parallel evaluations. The optimization was conducted using the Thompson Sampling Efficient Multi-Objective Optimization (TS-EMO) algorithm, developed by Bradford et al.^34^. Objective and cost functions are defined in the “Methods” section (Eqs (1) and (2)). To manage the computational expense of FEA and CFD, we conducted large-scale parallel simulations.

**Fig. 2:**
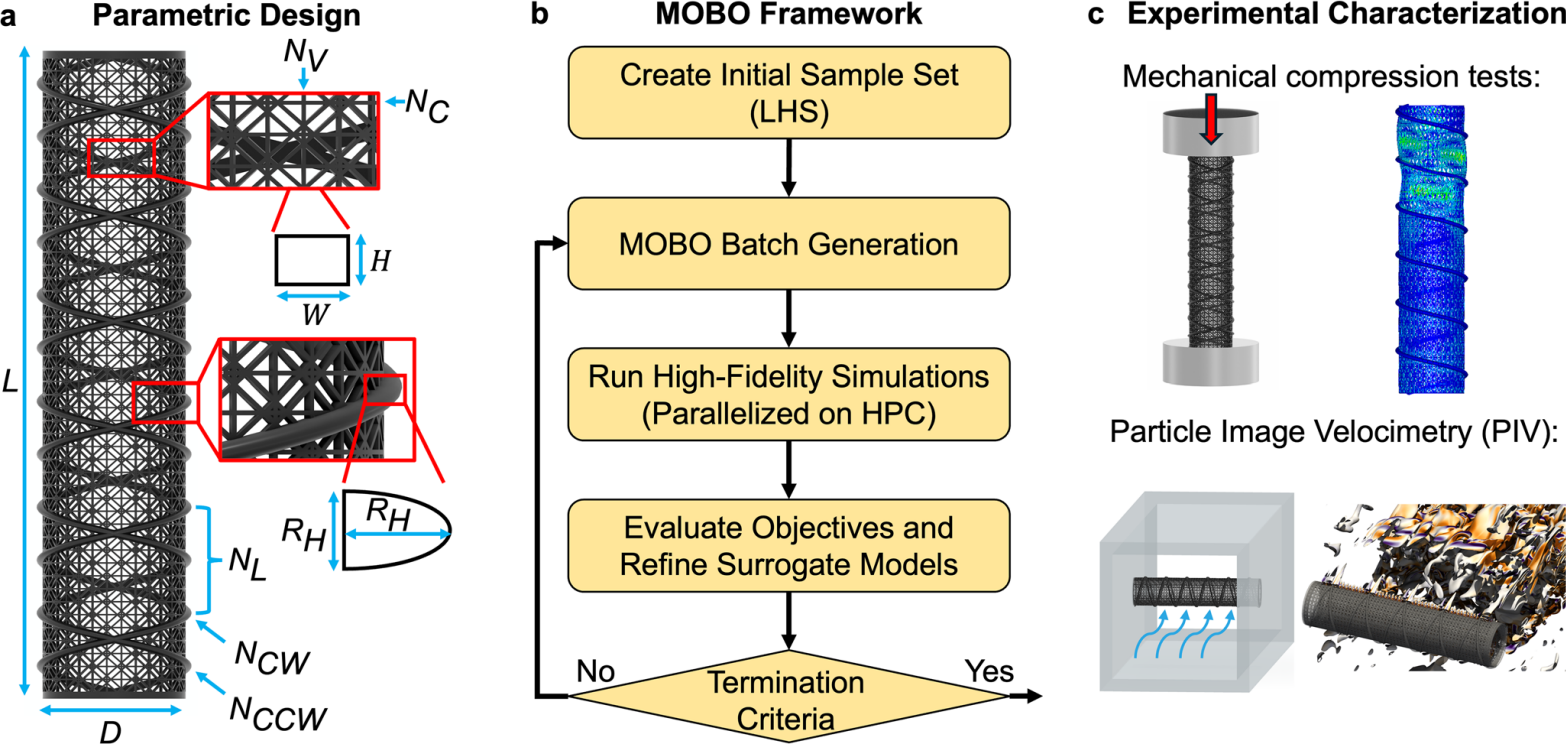
Schematic of the bioinspired design, optimization, and experimental validation framework. **a** Schematic of the parametric sponge-inspired lattice design, in which geometric parameters, including beam width (*W*), beam height (*H*), number of vertical (*N*_V_) and circumferential (*N*_C_) elements, and helical ridge parameters, are derived from the architecture of *Euplectella aspergillum*. These parameters define an idealized cylindrical lattice used for computational modeling. **b** Flowchart illustrating the multi-objective Bayesian optimization workflow. The process begins with an initial set of 50 design samples generated using Latin hypercube sampling, followed by iterative batch generation and evaluation. Batches of size 20 are executed in parallel on high-performance computing resources, where high-fidelity finite element and computational fluid dynamics simulations are used to evaluate the multi-objective cost functions capturing mechanical and fluid dynamic performance. Surrogate models are updated iteratively, and the process continues until the convergence criteria are met. **c** Schematic of experimental characterization of selected Pareto-optimal designs. Mechanical compression tests are used to assess structural performance, while stereo particle image velocimetry is used to measure flow behavior and validate the computational predictions.

Our multi-stage workflow is summarized in Fig. 2. It starts with parameterization of the sponge-inspired lattice, including helical ridges and diagonal reinforcements (Fig. 2a). MOBO then evaluates candidate designs through iterative FEA and CFD simulations to capture both mechanical and fluidic performance (Fig. 2b). The resulting Pareto-optimal geometries illustrate different trade-offs between strength and flow efficiency, with potential applications in wind-resistant structures, biomedical implants, and fluid management devices. Selected designs were fabricated and tested through compression experiments and SPIV measurements (Fig. 2c). These experiments provided validation of the computational results. Details of the simulation, optimization, fabrication, and testing procedures are provided in the “Methods” section.

### Pareto front analysis

**Fig. 3:**
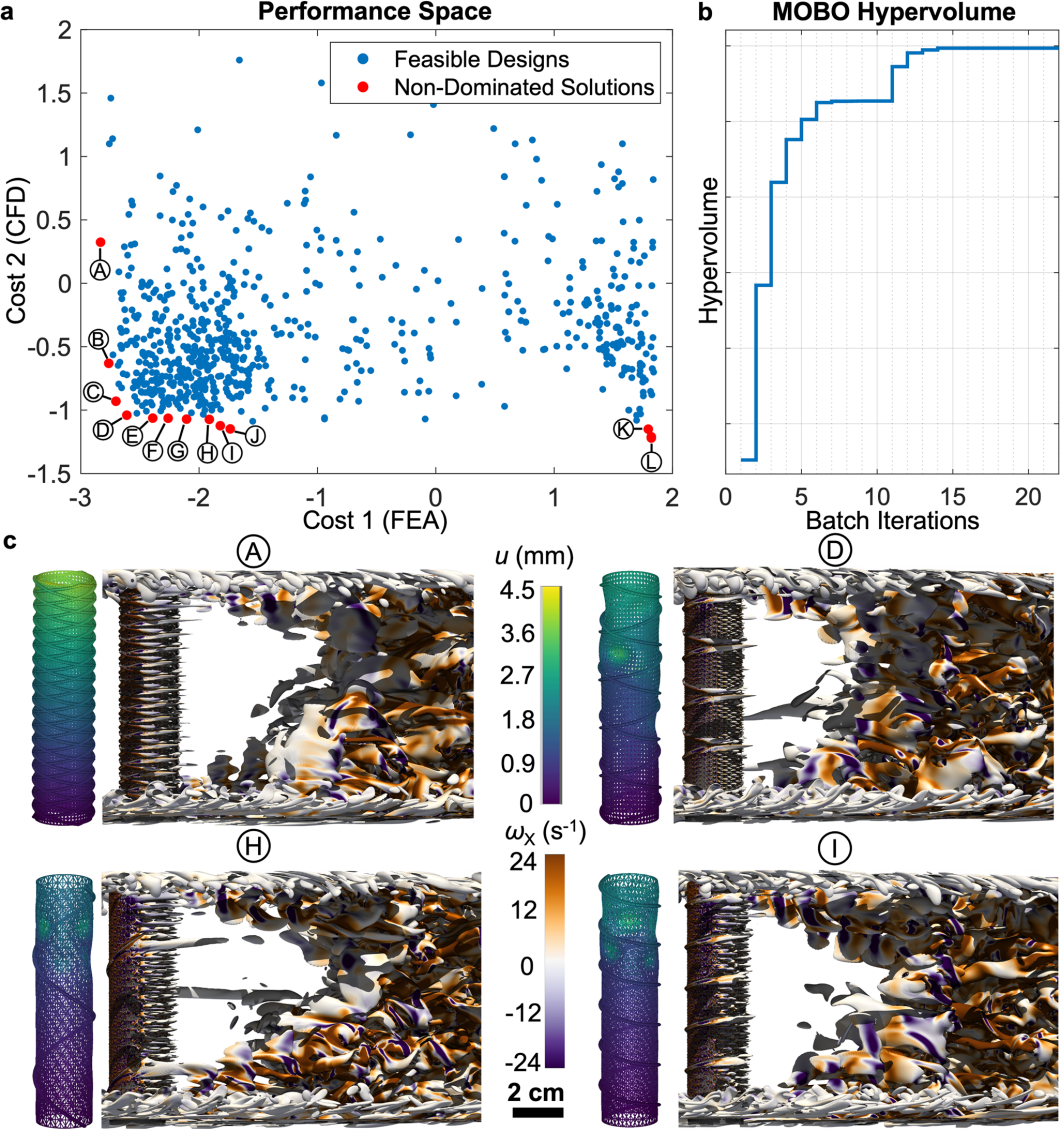
Pareto front and representative designs from multi-objective optimization. **a** Normalized cost-function space (see “Methods”, Eqs (1) and (2)), showing the trade-off between fluidic efficiency and mechanical robustness. Blue points represent feasible designs, and red points indicate non-dominated (Pareto-optimal) solutions (A–L). Design A corresponds to high mechanical strength, while design L prioritizes fluid dynamic efficiency. **b** Hypervolume progression during the multi-objective Bayesian optimization. Improvement levels off in later batches, suggesting that further iterations would yield minimal gains, providing a natural stopping criterion for the optimization. **c** Simulations of selected designs (A, D, H, and I). For each design, finite element analysis results (left) show displacement magnitude (*u*, mm), and computational fluid dynamics results (right) show vorticity magnitude (*ω*_x_, s^−^¹). Colorbars indicate field magnitude and units. These designs illustrate different trade-offs along the Pareto front and were selected for experimental validation. Source data are provided as a Source Data file.

The optimization produced a design space mapping the trade-offs between mechanical performance, defined as the critical buckling load normalized by volume, and fluidic performance, quantified by reductions in drag force, lift force, and vortex-shedding intensity (Fig. 3a; see “Methods”, Eqs (1) and (2), for full cost-function definitions). Pareto-optimal designs are shown in red. Convergence was monitored using the hypervolume metric (Fig. 3b), which leveled off in later batches, indicating that further iterations would likely add little benefit and providing a natural stopping point. A complete list of evaluated designs, including their geometric parameters and corresponding cost-function values, is provided in Supplementary Data 1, and the evolution of the optimization process across iterations is illustrated in Supplementary Movie 1.

The Pareto front makes clear that gains in one objective come at the expense of the other. Simulations of selected designs (Fig. 3c) highlight these trade-offs: Design I, for example, is optimized for reduced vortex shedding and drag, while design A emphasizes mechanical strength. These examples highlight the balance between fluid stability and structural strength, influenced by features such as helical ridge density, lattice configuration, and beam cross-section.

To further quantify how geometric parameters influence the optimization landscape, we performed a sensitivity analysis on the MOBO dataset using Pearson correlations, Spearman rank correlations, and standardized regression coefficients, with full results presented in the Supplementary Notes and Supplementary Fig. 11. All three metrics consistently identify the beam cross-section dimensions *H* and *W*, and the number of vertical members *N*_V_, as the dominant contributors to mechanical performance, with correlation magnitudes in the range of ~0.4–0.85. This is consistent with structural mechanics theory, as these parameters directly increase bending stiffness, moment of inertia, and load-bearing pathways, thereby governing buckling resistance.

In contrast, the fluidic objective exhibits weaker and more distributed sensitivities across all parameters ($\left|{\rho }_{{{\rm{i}}}}\right|$ ≲0.3), indicating that drag, lift, and vortex shedding arise from multivariate geometric interactions, such as global porosity distribution, rather than a single controlling parameter. The moderate cross-correlation between the two objective functions (≈ 0.25 –  0.27) confirms that mechanical and fluidic objectives are partially competing but not redundant, reinforcing that the optimization problem is genuinely multi-objective and well-suited for a MOBO framework.

Analysis of clusters and outliers along the Pareto front further highlighted trends. For example, modifying helical ridge pitch can reduce vortex shedding with only modest reductions in mechanical strength. The Pareto front thus provides a practical tool for selecting designs based on application priorities. Four representative points were chosen for experimental validation, spanning designs from fluidically efficient to mechanically robust. Full parameter configurations and images of Pareto-optimal designs are given in the Supplementary Table 2 and Supplementary Figs. 9 and 10.

### Fabrication and experimental validation of selected designs

Designs A, D, H, and I were selected from the Pareto front to represent a spectrum of performance attributes, covering mechanically robust, fluid-efficient, and balanced designs.

The selected designs were fabricated using an Anycubic Photon D2 DLP printer with a resolution of 51 µm, enabling features and pore sizes as small as 150 µm, comparable to those of the deep-sea sponge. The printed geometries were validated with optical microscopy to confirm dimensional accuracy. Mechanical performance was assessed by measuring critical buckling load and deformation under compression. Results showed high repeatability across four samples, confirming the reliability of the fabrication process.

**Fig. 4:**
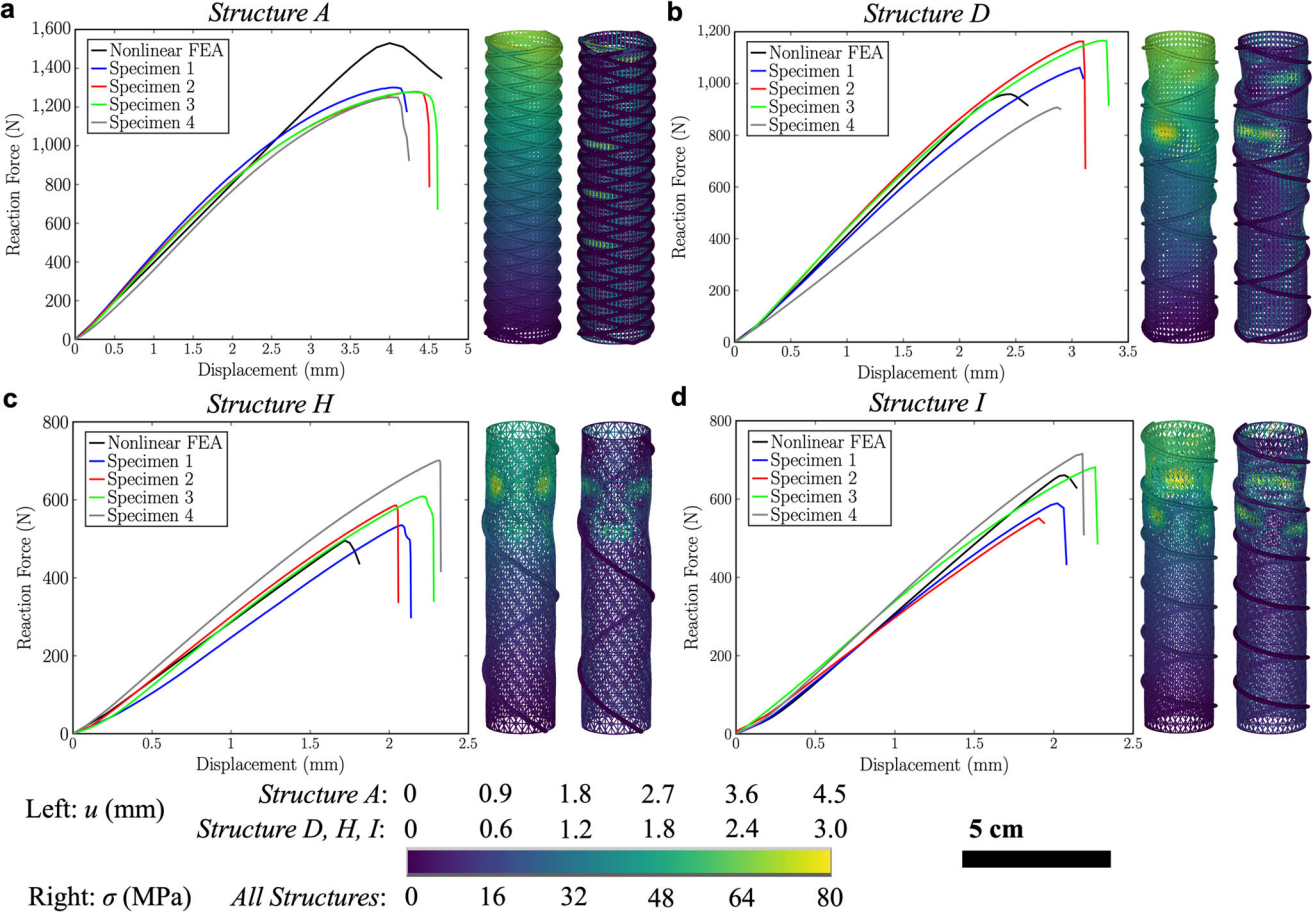
Compression tests and nonlinear finite element validation of Pareto-optimal designs. **a**–**d** Comparison of experimental and simulated force–displacement responses for four representative designs (A, D, H, and I) selected from the Pareto front. In each panel, the solid black line represents nonlinear finite element analysis predictions, while colored curves correspond to experimental measurements from four independently fabricated specimens. The results show excellent agreement between simulations and experiments across all designs, with deviations in effective stiffness below 5%. All structures exhibit an initial linear elastic regime followed by nonlinear stiffening and a peak force corresponding to the onset of buckling. The location and magnitude of the peak load are consistently captured by the simulations, indicating accurate prediction of critical buckling behavior. Post-buckling responses show slight variability between specimens, reflecting sensitivity to fabrication imperfections and geometric tolerances at small length scales. Representative deformation fields from finite element analysis are shown alongside each plot, illustrating displacement magnitude (*u*, mm) and equivalent (von Mises) stress (*σ*, MPa). Colorbars indicate field magnitude and units. The deformation patterns highlight the spatial localization of buckling and stress concentration regions, which are consistent with experimentally observed failure modes (see Supplementary Fig. 2 and Supplementary Movies 2–5). Across all designs, the results confirm that the computational framework accurately captures both global stiffness and local instability mechanisms, validating the predictive capability of the combined optimization and simulation approach. Source data are provided as a Source Data file.

Force–displacement curves from the experiments closely matched predictions from nonlinear buckling analyses (Fig. 4). Agreement was consistent across key metrics, including Young’s modulus (effective stiffness) and buckling behavior, with deviations between experimental and simulated stiffness values below 5% as shown in Supplementary Table 1. Experimentally observed buckling locations, shown in Supplementary Fig. 2 and Supplementary Movies 2–5, also matched simulated behavior, confirming the accuracy of the computational model.

**Fig. 5:**
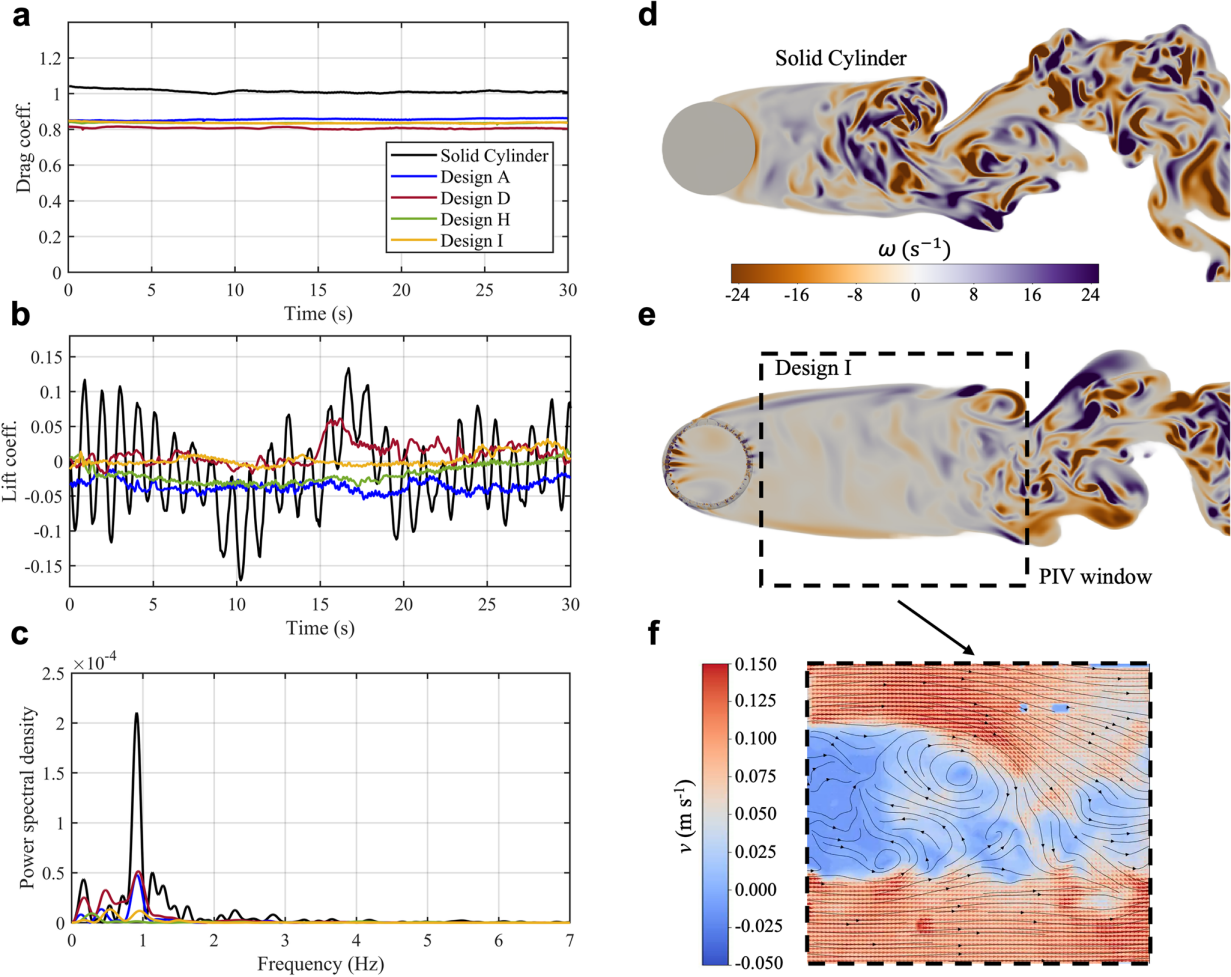
Hydrodynamic performance of optimized designs compared to a solid cylinder. **a** Drag coefficient over time obtained from computational fluid dynamics simulations. The solid cylinder exhibits a baseline drag coefficient of ~1.0, while the optimized designs reduce drag to values between 0.8 and 0.85. **b** Time-resolved lift coefficient under quasi-steady flow conditions. The optimized designs significantly reduce both the amplitude and variability of lift fluctuations compared to the solid cylinder. **c** Power spectral density of the lift force signal, showing a dominant vortex shedding frequency near 0.9 Hz (corresponding to a Strouhal number of approximately 0.21). The optimized designs exhibit reduced peak magnitudes, indicating suppression of vortex shedding and unsteady wake dynamics. **d** The vorticity field of the solid cylinder, showing strong alternating vortices forming in the near wake. **e** The vorticity field of optimized Design I, illustrating reduced vorticity magnitude and a downstream shift of vortex formation. The dashed region indicates the measurement domain used for stereo particle image velocimetry. **d**, **e** Color indicates vorticity (*ω*, s^−^¹). **f** Representative stereo particle image velocimetry measurement of the wake behind Design I. Background color indicates velocity (*v*, m s^−^¹), while vectors and streamlines illustrate flow direction and structure. Across all optimized designs, hydrodynamic loading is significantly reduced relative to the solid-cylinder reference, with both drag and lift fluctuations suppressed due to delayed and weakened vortex shedding. Source data are provided as a Source Data file.

Validation of fluidic performance was performed using two complementary approaches: a CFD baseline simulation of a solid cylinder and SPIV measurements on the fabricated lattice structures. The solid cylinder served as a reference case for validating our CFD formulation, with detailed comparisons presented in Supplementary Fig. 6. SPIV provided time-resolved velocity fields under quasi-steady flow at a Reynolds number of ~2100, enabling a direct assessment of wake behavior and unsteady loading. As shown in Fig. 5, all optimized designs, regardless of porosity, substantially reduce hydrodynamic loading relative to the solid-cylinder benchmark.

For the reference solid cylinder, the drag coefficient remained close to 1.0, consistent with the literature. The optimized designs reduced drag to 0.8– 0.85 (Fig. 5a). Lift fluctuations were also suppressed (Fig. 5b): Design A, the most rigid structure, reduced the RMS lift coefficient by 39%, while the most porous, Design I, achieved an 84% reduction. Power spectral density of the lift signal (Fig. 5c) confirmed the attenuation of vortex shedding, with all optimized designs showing reduced spectral peaks near 0.9 Hz, matching the expected Strouhal number of ~0.21.

Wake dynamics are illustrated by vorticity plots of the solid cylinder (Fig. 5d) and optimized Design I (Fig. 5e). The cylinder generates strong alternating vortices close to the body, whereas Design I shifts vortex formation downstream and lowers overall vorticity. These trends were supported by SPIV measurements recorded within the wake region, from the cylinder up to approximately three diameters downstream, as indicated by the dashed box in Fig. 5e. A representative SPIV snapshot is shown in Fig. 5f, with velocity magnitude shown by background color and overlaid streamlines and vectors illustrating flow direction and structure. Further views of the wake structure and vorticity fields for the four optimized designs are shown in Supplementary Figs. 12 and 13 and Supplementary Movies 6 and 7.

To compare CFD and SPIV data, velocity fields were extracted from simulations over the same spatial domain. Side-by-side snapshots and time-resolved movies (Supplementary Figs. 14–16 and Supplementary Movies 8–11) show agreement in velocity magnitude and flow structure. Some differences were observed between SPIV and CFD results as detailed in Supplementary Notes: vortex shedding appeared more pronounced and initiated closer to the structure in the SPIV data, likely due to fabrication-induced surface roughness or boundary effects in the water tunnel. Nonetheless, both experimental and numerical data confirm the similar wake topology and reduction in unsteady flow. Quantitative comparisons presented in Supplementary Fig. 17 support these findings. Dominant shedding frequencies from CFD and SPIV matched within ~20%. Average transverse (lift direction) velocities in the wake were on average 0.011 m s^−1^ higher in SPIV, consistent with the more visible shedding. Importantly, both datasets showed that optimized designs reduced transverse velocities by a factor of three to five and lowered vortex intensity compared to the solid cylinder baseline, confirming the effectiveness of the optimization in stabilizing wake dynamics and reducing hydrodynamic forces.

## Discussion

This study establishes a robust design framework for multi-objective optimization of multifunctional structures that balance mechanical stability, quantified by the critical buckling load normalized by volume, and fluidic performance, quantified by drag, lift, and vortex-shedding suppression. Inspired by the hierarchical skeleton of *E.a*., we couple fluid dynamics and structural mechanics through high-fidelity simulations and experiments. Using automated FEA and CFD simulations with MOBO on HPC resources, our framework identifies Pareto-optimal designs that balance competing objectives. Each design represents a trade-off between mechanical strength and fluidic efficiency, echoing the dual functionality of the *E.a*., where structural integrity and flow permeability are simultaneously maintained under extreme conditions.

The optimization yields substantial mechanical performance gains relative to baseline geometries, with the critical buckling load increasing on average by ~ 140% compared to the initial Latin Hypercube samples while maintaining or reducing material usage. Sensitivity analysis (Supplementary Notes and Supplementary Fig. 11) provides insights into how this improvement is achieved: beam cross-section dimensions *H* and *W* and the number of vertical beams *N*_V_ emerge as the dominant controls on mechanical performance, consistent with their influence on bending stiffness, moment of inertia, and primary load-bearing pathways. These trends, together with the strong agreement between nonlinear FEA and compression experiments in terms of buckling loads, force–displacement response, and failure locations (Supplementary Fig. 2), confirm that the optimization is guided by physically meaningful structure–property relationships.

Fluid dynamic efficiency is improved in parallel. The optimized lattices reduce drag and suppress unsteady lift relative to a solid-cylinder benchmark while preserving significant load-bearing capacity. In CFD, drag coefficients of the optimized designs fall well below those of the solid cylinder and are consistent with reported values for far more porous sponge structures^21^. As detailed in the results section, unsteady lift forces are strongly attenuated across the design set, and SPIV measurements confirm the CFD predictions. The observed wake stabilization is consistent with prior findings that increasing porosity attenuates turbulence kinetic energy (TKE), elongates shear layers, and suppresses vortex shedding^43–46^. In our case, the time-averaged TKE in the cylinder wake was about 50% higher and peaked roughly one diameter closer to the body than in the optimized designs, while CFD predicted an even more pronounced downstream shift than observed in SPIV, as shown in Supplementary Fig. 16. Our results further show that porosities as low as 5% are sufficient to suppress vortex shedding, a significant reduction compared to the effective porosity of natural *E.a*. structures. This highlights how targeted geometric refinement through optimization can exceed biological benchmarks for specific functional metrics. While natural sponges may retain higher porosities due to multifunctional biological roles^47–49^, our designs isolate and enhance a subset of performance attributes. Prior work on perforated cylinders reported similar wake suppression at porosities around 8%^45^, with similar wake suppression mechanisms^43,46^. Although those studies were typically conducted at higher Reynolds numbers, our results show that optimization can achieve comparable benefits at lower porosities.

The interplay between mechanical and fluidic objectives is further clarified by the sensitivity analysis in the Supplementary Notes. While mechanical performance is dominated by a few geometric parameters, the fluidic objective exhibits weaker and more distributed sensitivities across all parameters, suggesting that drag, lift, and vortex shedding arise from collective geometric interactions rather than a single controlling variable. The moderate cross-correlation between mechanical and fluidic objectives indicates that they are partially competing but not redundant, reinforcing that the problem is genuinely multi-objective and well-suited to a MOBO framework rather than a weighted single-objective optimization.

**Fig. 6:**
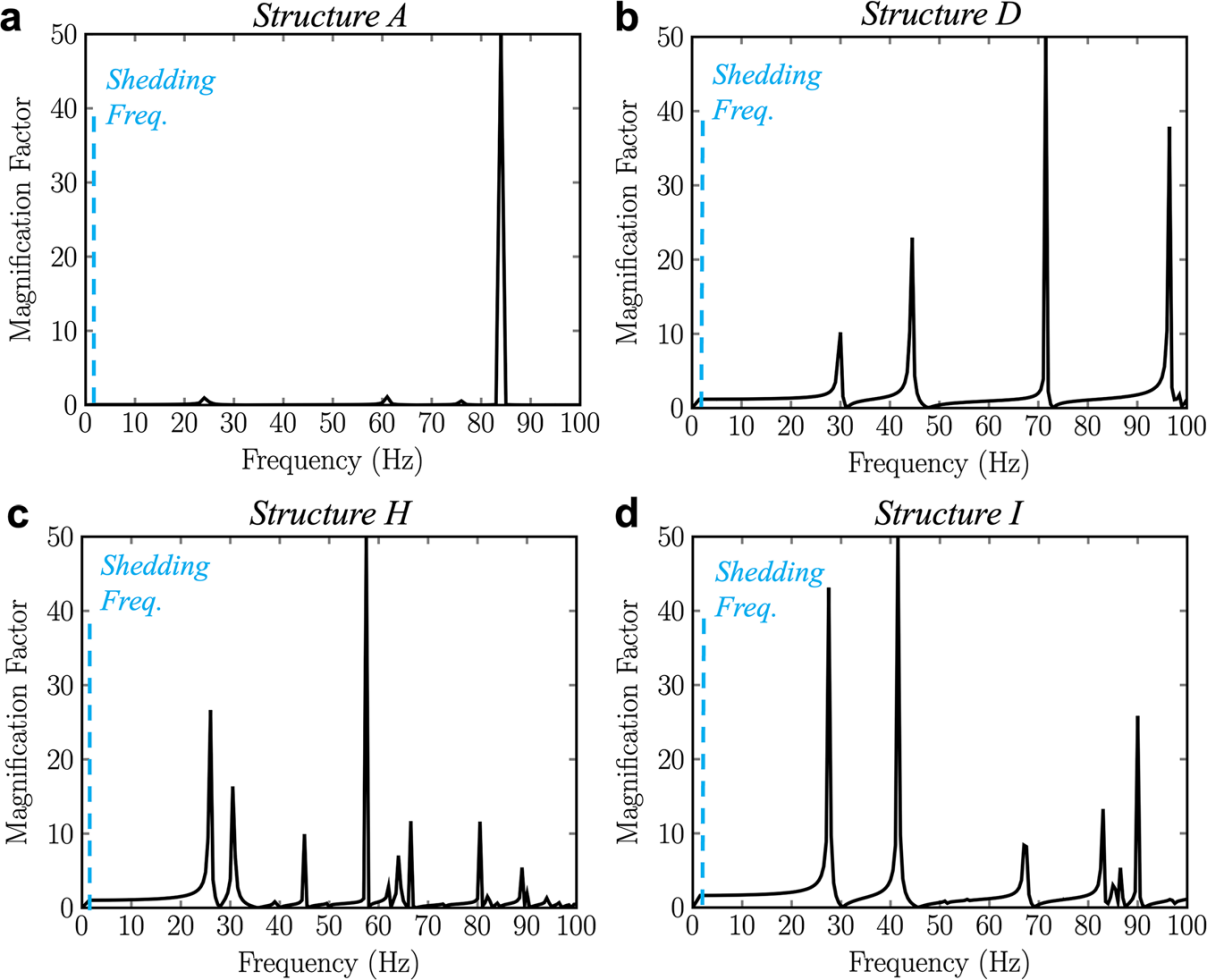
Harmonic response of Pareto-optimal designs under radial excitation. **a**–**d** Frequency response functions for designs A, D, H, and I, obtained from harmonic finite element analysis. The plotted magnification factor represents the normalized displacement amplitude under unit radial excitation applied at mid-height, with all nodes at the top and bottom surfaces fixed to mimic the experimental setups. Distinct peaks correspond to structural resonance modes of the lattice. For all designs, the first significant resonance occurs above 20 Hz, with additional higher-order modes appearing at larger frequencies depending on geometry. The dashed blue line indicates the characteristic vortex shedding frequency range (~0.85–1.1 Hz) obtained from computational fluid dynamics simulations and stereo particle image velocimetry measurements. The clear separation between fluid-induced excitation frequencies and structural resonance frequencies demonstrates that vortex shedding occurs well below the first structural mode. This spectral decoupling implies minimal energy transfer between fluid and structural dynamics under the operating conditions studied, supporting the validity of the decoupled simulation approach used during optimization. Source data are provided as a Source Data file.

Given the partially competing nature of the two objectives and the computational expense of fully coupled simulations, fluidic and mechanical analyses were decoupled during optimization. Harmonic FEA of the four Pareto-optimal designs (A, D, H, I) shows that the first structural resonance occurred above 20 Hz (Fig. 6), well above the vortex shedding frequencies of 0.85–1.1 Hz (Strouhal ≈ 0.21) obtained from CFD and SPIV. This spectral separation implies minimal energy transfer between global shedding modes and structural resonances, thereby justifying the decoupled approach for the operating conditions studied. Although valid under these conditions, future work should explore fully coupled fluid–structure interaction (FSI) models to capture transient dynamics, resonance interactions, and fatigue effects, especially at higher Reynolds numbers or under cyclical loading. While global vortex shedding occurs at frequencies well below structural resonance, localized shedding from smaller-scale features may occur at higher frequencies, potentially overlapping with structural modes. Modal analysis and dynamic testing could study these interactions and guide designs for long-term durability.

Beyond the specific performance improvements reported here, this framework demonstrates how multifunctional designs can be applied across engineering domains. In underwater settings, such as offshore platforms and pipelines, drag reduction and structural resilience are both critical. In biomedical applications, flow-permissive stents and filtration devices benefit from combining strength with low fluid resistance^29^. In aerospace and civil engineering, high strength-to-weight ratios are essential for components in bridges and aircraft. Other potential uses include energy-absorbing structures for transportation and sports^50^, high-throughput water purification and flow catalysts^26,51^, and multifunctional metamaterial solutions for sensing, energy harvesting, and communication^52,53^. Recent advances in cellular fluidics and bubble-resolved transport within architected lattices further highlight opportunities for programmable multiphase flow control in such systems^54,55^.

Our framework opens several paths for future work. Scalable fabrication approaches could bring these designs into industrial applications^56^. Machine-learning surrogates or reduced-order models may cut computational costs and speed up exploration of large design spaces. Further validation across different Reynolds number regimes, or the development of adaptive structures with tunable porosity and stiffness, would broaden applications in robotics, infrastructure, and flow control. The same approach can also be applied to other biological systems with multifunctional geometries, especially when combined with generative design and AI-based optimization. Together, these directions highlight the broader potential of bioinspired, computationally optimized architectures to enable next-generation multifunctional materials and structural systems.

## Methods

This section details the methodologies, parameters, and materials used throughout the study. It includes the multi-objective optimization framework, computational simulations, fabrication techniques, and experimental techniques applied.

### Multi-objective optimization framework

Two cost functions were defined to capture the competing objectives of structural strength and fluidic performance. The mechanical cost function *F*_mech_, Eq. (1):

1 $${F}_{{{\rm{mech}}}}=\,-\,{F}_{{{\rm{Buckling}}}}/{V}_{{{\rm{sponge}}}}$$

was designed to maximize the lattice’s buckling resistance while minimizing material usage, where *F*_Buckling_ is the standardized critical buckling force obtained from FEA, and *V*_Sponge_ is the lattice volume. The fluid dynamic cost function *F*_fluid_, Eq. (2):

2 $${F}_{{{\rm{fluid}}}}=\,\alpha \overline{{F}_{{{\rm{D}}}}}+\beta \overline{{F}_{{{\rm{L}}}}}+{{\rm{\gamma }}}{\sigma }_{{{{\rm{F}}}}_{{{\rm{L}}}}}$$

combined average drag forces $(\bar{{F}_{{{\rm{D}}}}})$, average lift $(\bar{{F}_{{{\rm{L}}}}})$ forces, and ${\sigma }_{{{{\rm{F}}}}_{{{\rm{L}}}}}$ the over time calculated standard deviation of the lift force, a measure for lift force oscillations and vortex shedding. Weighting factors were chosen as $\alpha=\beta=0.2$ and $\gamma=$ 0.6, to emphasize suppression of unsteady lift. The overall optimization problem was therefore formulated as a bi-objective optimization, minimizing *F*_mech_ and *F*_fluid_ simultaneously.

The optimization process was initialized with 50 Latin Hypercube samples^57^ to provide an initial coverage of the design space. Multi-objective optimization was then performed using the Thompson Sampling Efficient Multi-Objective (TSEMO) algorithm^34^ to generate Pareto-optimal solutions, iterating through 22 batches of 20 samples each. In this approach, independent Gaussian-process surrogate models are constructed for the mechanical and fluidic objective functions based on the evaluated designs. At each iteration, Thompson sampling is used to draw candidate objective functions from these surrogate models, and a multi-objective acquisition function is optimized to identify new evaluation points. The hypervolume quality indicator, together with the NSGA-II algorithm, is used to approximate the Pareto front and select new candidate designs for evaluation.

TSEMO was chosen because it efficiently approximates Pareto fronts, handles noisy objective functions, and supports batch evaluations, which are advantageous for computationally expensive simulations^34^. The AutoOED^58^ platform was used as a graphical interface to monitor optimization progress and adjust parameters. Additional background on the TSEMO algorithm and the AutoOED platform is available in Bradford et al.^34^ and Tian et al.^58^.

Given the high computational cost of high-fidelity FEA and especially CFD simulations, all runs were carried out on the FASRC Cannon cluster at Harvard University. Each job was executed on a Sapphire Rapids node with 112 processes. Structures were evaluated in batches of 20, which allowed efficient parallelization. While batch optimization may require more evaluations to converge, running designs simultaneously minimized overhead and enabled much greater throughput within the same timeframe. In total, the CFD simulations required ~10,000 node-hours.

Automation was achieved through parameterized Python scripts that generated geometries of the sponge-inspired lattices. These were passed directly to CFD and FEA solvers, with data management and transfers handled via the Globus platform^59^ (web-based platform). Once the convergence criteria were met, selected designs from the Pareto front were fabricated and validated experimentally through compression tests and SPIV.

### Finite element analysis (FEA) for mechanical properties

Finite element analysis was used to predict the mechanical response of each design, focusing on critical buckling load, stiffness, and failure points. Models were created in ANSYS^®^ Mechanical™ 2024 R1 using the Parametric Design Language (APDL) and automated through PyMAPDL, which enabled direct scripting of lattice geometries and simulation runs. Different lattice configurations were generated by varying geometric parameters in the code.

All lattice geometries were discretized using Timoshenko beam elements, selected for their ability to capture bending and shear deformation in slender load-bearing members. A maximum element length of 1 mm was adopted for all optimization and validation simulations. This value was determined through a mesh-refinement study in which the element length was progressively reduced until the computed critical buckling load changed by less than 3% compared to the highest-resolution mesh (0.125 mm). Thus, the selected mesh resolution provides an appropriate balance between computational efficiency and numerical accuracy during the multi-objective optimization. A representative mesh and the corresponding convergence curve are presented in Supplementary Fig. 1. Linear elastic material properties were assigned based on compression tests of solid, cured Anycubic DLP Craftsman resin, yielding a Young’s modulus of 1.1 GPa and a Poisson’s ratio of 0.49. For each design, the cylindrical lattice was assumed to be fully constrained at its base (*u*_x_ =  *u*_y_ = *u*_z_ = 0). Two types of structural simulations were performed. First, a linear static analysis was used to evaluate the elastic stiffness of each lattice. Second, the critical buckling load was obtained using a linear perturbation eigenvalue buckling analysis, which computes the first buckling mode and its associated load factor under a uniform compressive pressure applied on the top surface along the z-axis. For the Pareto-optimal designs, we additionally performed geometrically nonlinear buckling simulations to capture large-deformation behavior and post-buckling response. Initial geometric imperfections were introduced by scaling the first buckling eigenmode obtained from the linear eigenvalue buckling analysis. This eigenmode-based imperfection approach perturbs the geometry along the most critical buckling mode and is widely used to trigger physically realistic instability in nonlinear FEA^60,61^. An imperfection amplitude of 1% of the normalized eigenmode displacement field, scaled relative to the lattice diameter, was applied, providing a small perturbation sufficient to initiate the dominant buckling path without artificially reducing the predicted load capacity. The nonlinear simulations provided improved predictions of load-bearing capacity and stress distribution, and the predicted failure locations closely matched experimental observations. This confirmed the validity of the computational model and offered deeper insight into the failure mechanisms and robustness of the sponge-inspired designs. Additional harmonic simulations were conducted for final designs with boundary conditions applied to replicate the SPIV setup, as further detailed in Supplementary Methods.

### Computational fluid dynamics (CFD) simulations

CFD simulations were used to analyze the fluidic behavior of the designs, focusing on vortex shedding, drag, and flow stability. Parametric geometries were converted to STL files using custom Python scripts provided as Supplementary Software and imported into the CFD solver for analysis.

All simulations were performed using the open-source solver Basilisk^39^ (release 25-02-26), which solves the incompressible Navier–Stokes equations using a second-order, cell-centered finite-volume formulation on an adaptive octree grid^62,63^. Solid regions were imposed through Brinkman penalization, implemented as a resistive body-force term following the formulation of Angot et al.^40^, enabling accurate no-slip boundary conditions on complex lattice geometries without explicit body-fitted meshing. Adaptive mesh refinement (AMR) dynamically refined the grid in regions of high velocity gradients, vorticity, and wake structures, while coarsening it in quiescent regions to reduce computational cost without sacrificing accuracy. The approach follows Popinet et al.^64^ and enables efficient and robust resolution of flow–structure interactions around slender beams and porous lattice architectures.

The top and front boundaries were treated as periodic during the optimization (perpendicular to the axis of the sponge). For the refined simulations of the selected, optimized geometries, no-slip wall conditions were applied to the top boundary, which was positioned adjacent to the ends of the sponge, as detailed in Supplementary Figs. 4 and 5. At the inlet (left boundary), a constant flat velocity profile was imposed, with zero normal gradients for pressure and face pressure. At the outlet (right boundary), the normal velocity gradient was zero, while the pressure and face pressure were fixed to zero, ensuring smooth outflow and a reference pressure level.

The domain size was *L* = 12.5*D*, where *D* is the diameter of the structure, and the mesh cell size was adaptively refined, ranging from $L/{2}^{8}$ to $L/{2}^{11}$ during the optimization and from $L/{2}^{10}$ to $L/{2}^{13}$ for the refined simulations. The finest mesh resolution, $L/{2}^{13}\approx 0.0015D$ was approximately five times smaller than the smallest structural features and pore sizes, ensuring accurate geometric representation and flow resolution. Coarser cells were retained in low-gradient areas to reduce cost. This two-fidelity strategy enabled efficient exploration of hundreds of candidate geometries during optimization while ensuring fully resolved flow fields for all reported quantitative results. Additional details and validation of the two-fidelity workflow are provided in the Supplementary Methods and Supplementary Fig. 3.

Viscosity was set to match the Reynolds number based on the inlet velocity and the radius of the sponge. The simulations were performed from the initial flat velocity in the domain up to 200 dimensionless time units. A method and implementation from Wald et al.^65^ was used for iso-surface extraction in post-processing. Visualization was performed using ParaView^66^ (v5.13.3). The complete code, along with documentation and execution scripts, is available at https://github.com/cselab/sponge and is permanently archived on Zenodo^67^.

### Fabrication

Selected designs from the Pareto front were fabricated with an Anycubic Photon D2 DLP printer (ANYCUBIC Technology Co., Ltd), which provides 51 µm resolution suitable for reproducing complex geometries. Printing was performed with Anycubic DLP Craftsman Resin, whose material properties (*E* = 1.1 GPa, *ν* = 0.49) were determined from Instron compression tests and used in simulations.

The optimized geometries were exported as STL files and processed using Photon Workshop (Anycubic, v3.6.x). Models were oriented with their longitudinal direction aligned along the build direction (z-axis, layer stacking direction). Support structures were applied only at the bottom layers using automated settings to ensure stable fabrication and facilitate removal. The sliced build files were generated directly in Photon Workshop, without additional g-code generation or external scripting workflows.

Fabrication parameters included an exposure time of 2.2 s, a layer thickness of 50 µm, and post-processing with cleaning and UV curing for 5 minutes to complete polymerization. Geometric accuracy was verified by optical microscopy, with all structures within ±50 µm of the STL models, consistent with the printer resolution.

Where deviations between the printed structures and the nominal CAD geometry were identified from microscopic measurements, minor adjustments were made in the CAD/STL files in CATIA (Dassault Systèmes, 3DX platform, R2024x) to better match the intended design dimensions in subsequent prints. These adjustments remained within the printer resolution and primarily served to compensate for small fabrication-induced geometric offsets.

### Mechanical testing

Mechanical tests were performed to validate the FEA predictions. Compression experiments were carried out on an Instron 5944 universal testing machine (Instron Corporation, Norwood, MA) using a 2 kN load cell (Instron 2580-2KN). Samples were compressed under displacement control at a rate of 0.1 mm s^−1^, corresponding to an engineering strain rate of 10^−3 ^s^−1^ for the 100 mm-tall specimens. This rate lies well within the quasi-static regime, ensuring a strain-rate–independent buckling response. Tests were recorded at 60 fps to capture the location and progression of failure, and load–displacement data were sampled every 0.02 s to quantify stress–strain behavior and buckling loads with high precision. For each design, four samples were tested to confirm repeatability.

### Stereo particle image velocimetry (SPIV)

SPIV was used to evaluate the fluidic performance of the fabricated designs, focusing on vortex shedding, flow stability, and drag reduction. Experiments were conducted in a vertical recirculating flow loop (Supplementary Figs. 7 and 8). The bulk flow rate was monitored using a Coriolis flowmeter, and rate was steady within 1%. The sponge samples were mounted 4.5 inches downstream of a 5th-order polynomial contraction and flow conditioner to ensure a nominal top-hat inlet flow profile.

3D velocity fields on a plane 5 mm from the sample’s centerline were obtained using Stereo PIV (SPIV)^68^. To achieve this, the water was seeded with Potters 110P8 hollow glass microspheres 5–25 µm in diameter. A dual-cavity Nd:YAG laser was used to illuminate a nominally 60 mm × 60 mm cross-section of the wake at a 4.76 mm offset from the channel centerline. Two FlowMaster Imager ProX PIV cameras (1600 × 1200 pixels) with Scheimpflug adapters were positioned to capture the wake from the rear of the cylinder to a region 2–3 diameters downstream.

SPIV image pairs with a nominally 3 ms interframe delay were recorded at a sampling frequency of 14 Hz, with 40 seconds of data collected for each test case. Velocity fields were reconstructed in DaVis 7.2 using a multi-pass cross-correlation algorithm, with a final interrogation window size of 32 × 32 pixels and 50% overlap. Detailed experimental results for varying sponge geometries are provided in the Supplementary Figs. 14–17.

### Reporting summary

Further information on research design is available in the Nature Portfolio Reporting Summary linked to this article.

## Supplementary information

Supplementary Information

Description of Additional Supplementary Files

Supplementary Movie 1

Supplementary Movie 2

Supplementary Movie 3

Supplementary Movie 4

Supplementary Movie 5

Supplementary Movie 6

Supplementary Movie 7

Supplementary Movie 8

Supplementary Movie 9

Supplementary Movie 10

Supplementary Movie 11

Supplementary Data 1

Supplementary Software

Reporting Summary

Transparent Peer Review file

## Source data

Source Data

## Data Availability

All data supporting the findings of this study are available within the Article and its Supplementary Information. Supplementary datasets containing geometric parameters, cost-function values, and Supplementary movies of simulations and experiments are provided with this paper for download. Source data are provided with this paper.

## Code availability

The code developed in this study is freely available at https://github.com/cselab/sponge and has been archived on Zenodo with a permanent 10.5281/zenodo.19136207. This archived version corresponds to the code used in this study. Selected scripts and workflows required to reproduce the main results are also provided with the paper as Supplementary Software files for download.
