# Supplementary material for: Optimized mechano-fluidic metamaterials inspired by deep-sea sponges
Source: Nat Commun. 2026 May 5;17:6062. doi: 10.1038/s41467-026-72612-4 (PMC13350808; doi:10.1038/s41467-026-72612-4)
Supplement: Supplementary file 1 — Supplementary Information [file 41467_2026_72612_MOESM1_ESM.pdf]

Supplementary Materials for

Optimized Mechano-Fluidic Metamaterials Inspired by Deep-Sea Sponges

Timon Meier *et al.*

Corresponding authors: Costas P. Grigoropoulos: cgrigoro@berkeley.edu,
Petros Koumoutsakos: petros@seas.harvard.edu

This supplementary document provides additional details to support the results presented in the main manuscript. It expands on the computational design framework, optimization methodology, mechanical modeling, fluid dynamics simulations, and experimental procedures. Extended figures, data, and analysis are included to illustrate the optimization process, validate simulation predictions, and clarify the performance trade-offs in sponge-inspired lattices. The material is intended to aid reproducibility and give further insight into the workflow and results discussed in the main text.

Supplementary Methods

Optimization Framework and Design Parameters

Design Parameterization

To design sponge-inspired lattices with both structural and fluidic functionality, we implemented a multi-objective optimization framework that combines geometric parameterization, automated simulation workflows, and Bayesian optimization, run on high-performance computing (HPC) resources.

The parameterization is based on the hierarchical skeleton of *Euplectella aspergillum*. Its cylindrical architecture was abstracted into a lattice of rectangular beam elements with diagonal and helical ridge reinforcements. Eight geometric parameters were defined to control the design space. The full set of parameters and ranges is summarized in Table 1 of the main manuscript.

For reproducibility, we provide a parametric Python script that generates STL files directly from user-defined parameter values. The output geometries are high-resolution 3D models, ready for both simulation and fabrication. As described in later sections, these designs were evaluated using Finite Element Analysis (FEA) for mechanics and Computational Fluid Dynamics (CFD) for flow behavior.

Optimization Algorithm and Workflow

We used the Thompson Sampling Efficient Multi-Objective Optimization (TSEMO) algorithm[1] to explore the high-dimensional design space and identify non-dominated solutions that balance mechanical and fluidic performance. TSEMO, constructs independent Gaussian-process surrogate

models for the mechanical and fluidic objectives and selects new samples by optimizing a multi-objective acquisition function via Thompson sampling, using the hypervolume quality indicator and NSGA-II to choose a new evaluation point at each iteration. This makes it well-suited for expensive black-box problems with noisy outputs and parallel evaluations.

The optimization was initialized with 50 Latin Hypercube Sampling (LHS) points[2] to provide broad coverage of the parameter space. From this baseline, optimization proceeded through 22 iterative batches of 20 designs each, giving 490 additional simulations. A batch size of 20 was chosen to balance parallel throughput with model accuracy, making efficient use of the available HPC resources. Larger batch sizes can accelerate convergence in wall-clock time but tend to reduce surrogate model accuracy between updates. Conversely, smaller batches increase model fidelity but underutilize HPC resources.

The workflow was implemented in Python, linking geometry generation, simulation automation, data handling, and AutoOED[3], the GUI-based platform used to monitor optimization progress and track Pareto front evolution. STL files for each design were generated and queued for simulation on the FASRC Cannon cluster at Harvard University, using Sapphire Rapids CPUs with 112 logical cores per node. Jobs were scheduled in parallel, with data transfer and monitoring handled via the Globus platform[4].

In total, the optimization consumed ~10,000 node-hours. Despite this cost, the framework converged efficiently, producing a well-defined Pareto front within the compute budget.

Finite Element Analysis (FEA) and Mechanical Testing

Mechanical performance of the sponge-inspired lattices was evaluated through FEA simulations in ANSYS® MAPDL 2024 R1, implemented in a fully parametric pipeline via PyMAPDL. This setup allowed automated geometry generation, meshing, loading, and execution within the optimization loop.

Boundary conditions were chosen to replicate the configuration used in the Instron compression experiments. The base of the cylindrical lattice was fully constrained ($u_x = u_y = u_z = 0$). The top nodes were fixed laterally ($u_x = u_y = 0$) while remaining free to move in the vertical direction. Material properties were obtained from experimental characterization of bulk-cured Anycubic Craftsman DLP resin and modeled as linear elastic with a Young's modulus of 1.1 GPa and Poisson's ratio $v = 0.49$.

For each design, two analyses were performed: a linear static analysis to determine axial stiffness, and a linear perturbation eigenvalue buckling analysis to compute the first critical buckling load and its associated mode shape, which served as the basis for subsequent nonlinear validation simulations described later. For the stiffness evaluation, a prescribed vertical displacement of 1 mm was applied to the top surface. For buckling analysis, a uniform compressive pressure was applied on the top surface along the z-axis.

All lattice geometries were discretized using Timoshenko beam elements (ANSYS BEAM188), which accurately capture both bending and shear deformation in high–slenderness-ratio members. A maximum element length of 1 mm was applied across all beam segments to ensure consistent discretization, though the geometric intersections that form structural nodes naturally result in many segments, and therefore finite elements, being shorter than the nominal maximum. Supplementary Fig. 1a presents a representative mesh with several zoomed-in views illustrating the nodal layout and segmentation. To verify mesh adequacy, we conducted a mesh-convergence study by progressively reducing the maximum element length and computing the associated

critical buckling loads. The resulting convergence curve (Supplementary Fig. 1b) shows that the buckling load asymptotically approaches a stable value as the mesh is refined. A maximum element size of 1 mm was therefore selected for all simulations throughout the optimization, as it achieves <3% deviation from the finest-resolution mesh while significantly reducing computational demands.

For the Pareto-optimal designs, we additionally performed geometrically nonlinear buckling simulations (NLGEOM = ON) to capture large-deformation behavior and post-buckling response. Initial geometric imperfections were introduced by scaling the first buckling eigenmode obtained from the linear eigenvalue buckling analysis using the UPGEOM command in Ansys. This eigenmode-based imperfection approach perturbs the geometry along the most critical buckling mode and is widely used to trigger physically realistic instability in nonlinear FEA[5,6]. An imperfection amplitude of 1% of the normalized eigenmode displacement field, scaled relative to the lattice diameter, was applied, providing a small perturbation sufficient to initiate the dominant buckling path without artificially reducing the predicted load capacity. These simulations yielded more accurate predictions of failure load and revealed stress redistribution patterns during collapse. A Python script used to generate geometries and run simulations is provided as a Supplementary Software File.

Harmonic Analysis

Harmonic FEA was performed for Designs A, D, H, and I to compare structural resonance with fluidic excitation frequencies. Simulations replicated SPIV boundary conditions: all nodes at the top and bottom surfaces were fixed in all directions. A unit radial force was applied at mid-height nodes in the circumferential (XY) direction. The frequency response was evaluated from 0–100 Hz in 0.1 Hz steps using the Harmonic Response module in MAPDL.

The displacement spectra showed the first significant resonance above 20 Hz (Fig. 6, main manuscript), well above the vortex shedding frequencies of 0.85 – 1.1 Hz measured in CFD and SPIV. This spectral separation supports the assumption of negligible fluid–structure coupling.

Experimental Testing and Validation

To validate the simulations, four Pareto-optimal structures (Designs A, D, H, and I) were fabricated using a high-resolution DLP printer with Craftsman resin. Each design was tested in quadruplicate under quasi-static uniaxial compression on an Instron testing system.

Axial stiffness (N mm^{-1}) and critical buckling load (N) were measured for each specimen and averaged across the four samples per design. Nonlinear FEA results were then compared to experiments by matching displacement and stress fields with failure locations recorded on video during testing. Snapshots from these recordings are shown in Supplementary Fig. 2, with full movies provided as Supplementary Movies 2 – 5.

In Supplementary Fig. 2, nonlinear FEA predictions (stress and displacement fields, as in Fig. 4 of the main manuscript) are shown alongside experimental images for Designs A, D, H, and I. Insets highlight regions where buckling or localized deformation occurred. Across all four cases, the simulated peak displacement and stress regions closely matched experimentally observed failure locations.

Supplementary Table 1 summarizes axial stiffness and buckling loads from both simulation and experiment. Deviations are within experimental uncertainty and are attributed to typical factors such as fabrication tolerances, resin shrinkage, or minor misalignments during testing. The close

agreement across all four designs further confirms the robustness and reliability of the modeling approach.

Computational Fluid Dynamics (CFD)

CFD simulations were performed with Basilisk[7], an open-source finite-volume solver for incompressible flows. Here we apply Basilisk to solve the incompressible Navier–Stokes equations using a second-order, cell-centered finite-volume formulation on an adaptive octree grid**.** Solid regions were imposed with the penalization method (Brinkman penalization), which enforces no-slip conditions by treating the solid as a porous region of vanishing permeability. All simulations during the optimization stage were performed at Reynolds number Re = 2100 (based on lattice diameter and inflow velocity) to replicate typical deep-sea flow conditions for *Euplectella*-inspired structures.

A key advantage of Basilisk is its octree-based adaptive mesh refinement (AMR), which dynamically refines the grid in regions requiring higher resolution. We leveraged AMR to efficiently resolve flow features around the fine lattice geometry. Near solid surfaces, within narrow pores of the lattice, and in regions of strong velocity gradients (shear layers and vortices), the mesh automatically subdivided to smaller cells. By contrast, in quiescent regions far from the structure, a coarser mesh was maintained to save computational cost. The domain was a cube of side length L = 12.5D (chosen to encompass the structure and its wake). During the optimization simulations, we allowed mesh refinement from a base resolution of $L/2^8$ up to a finest resolution of $L/2^{11}$. This corresponds to cell sizes ranging from approximately 0.05D down to 0.006D, fine enough to capture the thinnest beams and flow through the lattice pores. For the refined simulations of the selected optimum designs, we increased the resolution by two levels (smallest cell size $L/2^{13}$ ~ 0.0015D) and raised the base resolution (to $L/2^{10}$) to better resolve the incoming flow and far-field wake. This finest resolution is roughly five times smaller than the smallest pores and beams in the lattice. This grid resolution exceeds the mesh density reported in DNS studies of cylinder wakes at higher Reynolds numbers[8] and sufficiently resolves all dynamically relevant flow scales. Combined with AMR, this strategy captured features across multiple length scales, from thin boundary layers on sub-millimeter beams to large-scale wake vortices, while requiring far fewer cells than an equivalent uniform grid, keeping fully resolved simulations computationally feasible.

The use of exploratory-resolution simulations (or reduced-order models) to guide the initial stages of evolutionary optimization is standard practice for computationally expensive problems in fluid mechanics[9–11]. It is important to note, that even at the exploratory-resolution AMR settings used during optimization, the simulated lift and drag forces on a solid cylinder baseline matched established literature values for Re ≈ 2100. This agreement confirms that the essential wake physics, including vortex-shedding frequency and near-wake structure, are adequately resolved at lower cost (see following section for cylinder benchmark validation).

To further demonstrate that the exploratory-resolution simulations remain sufficiently accurate to guide surrogate-model construction, we include an additional performance-space comparison (Supplementary Fig. 3). This figure compares the optimization-stage cost-function values to the corresponding high-resolution evaluations for the four Pareto-optimal structures (A, D, H, I). The final evaluations preserve the same ordering and relative spacing, and deviations between resolutions are small. This confirms that the exploratory AMR settings capture the correct global performance trends, drag, lift, and vortex-shedding intensity, that the MOBO surrogate model relies upon. Importantly, all quantitative results presented in the main manuscript, all comparisons

to experiments, and all visualizations are based solely on the final high-resolution simulations, ensuring that only the most accurate CFD evaluations inform the reported findings.

Boundary Conditions

We applied different boundary condition strategies for the initial optimization runs versus the final high-fidelity simulations used to validate SPIV experiments:

Optimization phase:

To minimize computational cost during optimization (Supplementary Fig. 4), we employed a simplified boundary condition setup. A steady inflow velocity was imposed on the left boundary, and an outlet was defined downstream on the right. All other domain boundaries, including top, bottom, and lateral directions, were treated as periodic. This configuration effectively mimics an unbounded domain while allowing for a compact simulation setup.

Experimental Validation Phase:

For high-fidelity simulations of the solid cylinder and optimized designs A, D, H, and I (Supplementary Fig. 5), we applied realistic open-channel conditions to enable comparison with experimental SPIV data. The upstream boundary (left) was set as an inflow with a uniform velocity profile, while the downstream boundary (right) was an outflow with a zero-pressure-gradient (Neumann) condition to approximate convective exit flow and minimize reflection artifacts. The top and bottom boundaries were modeled as no-slip walls (rigid lids), simulating a confined channel with stationary upper and lower surfaces. While the SPIV experiments used a water tunnel with no-slip walls in both vertical and lateral directions, we applied periodic boundary conditions laterally to reduce computational cost. This choice introduces a slight deviation from the experimental setup but is not expected to significantly impact the near-field flow dynamics or vortex shedding behavior of interest.

All simulations assumed incompressible flow with constant fluid properties. The flow was initialized from rest at $t = 0$, and an impulsive start was applied by immediately setting the velocity to its target value. This approach introduced initial transients, which were allowed to dissipate before data collection began. Each case was then simulated until $t = 200$ (in nondimensional time units based on inflow velocity and lattice diameter), long enough for the flow to reach a quasi steady state or periodic shedding regime. We recorded flow field snapshots and force data throughout the simulation after an initial transient period (~50 time units) to analyze the established force and flow patterns.

Hydrodynamic Benchmarking and Validation of the Solid Cylinder

Before analyzing the performance of the optimized sponge-inspired structures, we validated our numerical approach by simulating flow around a solid circular cylinder at Reynolds number $\mathrm{Re} = 2100$, representative of our experimental conditions. This benchmark case serves to confirm the fidelity of our numerical setup, mesh resolution, and force computation routines.

Supplementary Fig. 6 summarizes the hydrodynamic response of the solid cylinder. Supplementary Fig. 6a and b show the time evolution of drag and lift coefficients after the flow reaches a quasi-steady state. The lift coefficient exhibits clear periodic oscillations, indicative of vortex shedding. The power spectral density (PSD) of the lift signal (Supplementary Fig. 6c) identifies a dominant shedding frequency near 0.9 Hz, which corresponds to a Strouhal number of $\mathrm{St} = \frac{fD}{U_0} \approx 0.21$, where f is the shedding frequency, D is the cylinder diameter, and U_0 is the free-

stream velocity. This value is consistent with literature data for circular cylinders at similar Reynolds numbers[12]. The time-averaged drag coefficient obtained from our simulations was approximately $c_D \approx 0.99$, also in excellent agreement with established reference values for a smooth circular cylinder at Re ~ 2100[13], where drag coefficients are typically reported in the range of 0.9 – 1.1. Supplementary Fig. 6d and e compare our computed drag and Strouhal number with empirical curves from literature[12,13], showing strong agreement across both metrics. This validates the simulation framework and provides a solid reference point for evaluating the hydrodynamic improvements of the optimized designs.

SPIV Measurements and Flow Loop Setup

To characterize the fluidic performance of the optimized sponge-inspired designs, Stereo Particle Image Velocimetry (SPIV) was used to measure flow behavior in the wake region, focusing on vortex shedding, flow stability, and drag reduction. Experiments were conducted in a vertical recirculating water tunnel, with the setup schematically shown in Supplementary Fig. 7.

The sponge samples were mounted 4.5 diameters downstream of a 5th order polynomial contraction and flow straightener to ensure a uniform inlet velocity profile. Flow rate was regulated using a 2.5-inch ball valve and variable frequency drive (VFD) and monitored in real-time via a Coriolis flowmeter. This resulted in flow steady within 1% with a nominally top-hat profile. A dual-cavity Nd:YAG laser generated a planar laser sheet aligned 4.76 mm off the sample centerline, illuminating the wake region. The flow was seeded with Potters 110P8 hollow glass microspheres (5–25 μm) to serve as tracer particles.

A pair of FlowMaster Imager ProX 1600 × 1200 pixel cameras with Scheimpflug adapters were arranged in a stereoscopic configuration to capture 3D velocity data in a vertical plane extending from the rear of the sample to 2–3 diameters downstream. Image pairs were recorded at 14 Hz for 40 seconds per test case.

Supplementary Fig. 8 highlights the SPIV measurement region. The left panel shows a representative CFD snapshot of the flow around a solid cylinder at the center plane, with the SPIV domain overlaid. The right panel presents a sample velocity field visualization, with streamlines and velocity magnitude illustrating the resolved flow dynamics in the wake region. Velocity fields were reconstructed using LaVision DaVis 7.2 software via a multi-pass cross-correlation algorithm, with a final interrogation window of 32 × 32 pixels with 50% overlap, corresponding to a 0.8 mm spatial resolution. In addition, custom Python and MATLAB scripts were used to further analyze the SPIV raw data.

Supplementary Notes

Decoupling of mechanical and fluidic simulations

The optimization problem was formulated as a bi-objective minimization of mechanical and fluidic costs, with cost functions defined in the Methods section (Eqs. 1 and 2) of the main manuscript. Although both objectives were optimized together, the simulations themselves were performed as decoupled models.

This choice was justified by spectral analysis from harmonic FEA and comparison with flow-induced force spectra. Boundary conditions in the harmonic FEA were chosen to represent the clamping and loading conditions of the SPIV setup (see Simulation section).

For the fluidic side, vortex shedding frequencies were estimated using the Strouhal relation, $\mathrm{St} = fD/U$, which is approximately 0.2 for cylinders across a wide Reynolds number range[12]. In our case, the mean lattice diameter was 22 mm (with slight variations of the outer diameter D from wall thickness parameter W). For $\mathrm{Re} = 2100$, dominant force and velocity fluctuations were measured between 0.85 – 1.1 Hz. These values were consistent between SPIV data and CFD force spectra.

Using $\mathrm{U} = \nu\mathrm{Re}/D$, with $\nu = 10^{-6}$ $\mathrm{m^2\ s^{-1}}$ (water at room temperature), this corresponds to

$$\mathrm{St}_{2100}=0.9\cdot 22.7/97.3\approx 0.21,$$

in agreement with classic vortex shedding behavior of solid cylinders.

By contrast, the first structural resonance occurred at ~20 Hz in harmonic FEA (Fig. 6, main manuscript). Since shedding frequencies are more than an order of magnitude lower, energy transfer between flow and structure is expected to be negligible. Although smaller-scale features could in principle shed at higher frequencies, these would likely be weak and incoherent across the lattice. Thus, decoupling the simulations is both computationally efficient and physically justified, capturing the dominant physics while avoiding fluid–structure interaction (FSI) complexity.

Optimization convergence and Pareto front

The design performance space is shown in Fig. 3a of the main manuscript, with mechanical cost on the X-axis and fluidic cost on the Y-axis (both minimized). Convergence was monitored using the hypervolume indicator (Fig. 3b), which increased steadily before plateauing in the final batches, indicating convergence of the Pareto front and providing a natural stopping criteria for our optimization.

The final front (red points in Fig. 3a) comprises 12 non-dominated solutions labeled A–L, spanning the trade-off between structural robustness and hydrodynamic efficiency. For example, Design A maximizes buckling resistance at the expense of higher fluid forces, while Design L minimizes vortex shedding with reduced mechanical strength. Visualizations of all Pareto-optimal structures A – L are provided in Supplementary Fig. 9 and 10.

A full summary of the Pareto-optimal solutions is provided in Supplementary Table 2, which lists the design ID, all eight geometric parameters, critical buckling load, structural volume, and the corresponding mechanical and fluidic cost values.

To illustrate how the optimization progressed, Supplementary Movie 1 shows the geometries of all evaluated designs in chronological order. The animation highlights the algorithm's search path. In addition, a Supplementary Data File is provided containing all 490 evaluated designs. The dataset includes the geometric parameters along with the computed mechanical and fluidic costs.

Sensitivity Analysis

To quantify how geometric design parameters influence mechanical and fluidic performance within the MOBO framework, we performed a sensitivity analysis using three complementary statistical measures: Pearson correlation (Supplementary Fig. 11a), Spearman correlation (Supplementary Fig. 11b), and standardized regression coefficients (SRC; Supplementary Fig. 11c). Together, these metrics characterize linear, monotonic, and regression-based parameter–objective relationships across the MOBO dataset.

Both the Pearson and Spearman heatmaps (Supplementary Fig. 11a, b) reveal consistent trends. The beam cross-section dimensions H (height) and W (width), along with the number of vertical beams N_V, exhibit the strongest influence on the mechanical objective, with correlation magnitudes ranging from 0.4 to 0.85 across the two metrics. This behavior aligns with structural mechanics theory, as these parameters directly increase the cross-sectional moment of inertia, stiffness, and the number of available load-bearing pathways, which collectively govern critical buckling performance. The predominantly negative correlations arise from the definition of the mechanical cost function, which decreases for improved buckling resistance.

In contrast to the mechanical objective, the fluidic objective exhibits weaker and more diffuse correlations, typically in the range of |0.05 – 0.27|. This behavior is expected: flow through open, multiscale lattice architectures is governed by nonlinear flow–structure interactions, vortex shedding, and local pore topology, none of which are controlled by any single geometric parameter. Fluidic performance likely emerges from global porosity distribution and interactions between helical ridges, explaining the broader spread and lower magnitude of correlation values. Sign changes across parameters reflect the differing sensitivities of drag, lift, and lift fluctuations to specific geometric modifications.

The SRC distributions (Supplementary Fig. 11c) further support these observations. Across repeated bootstrapped regression fits, H, W, and N_V consistently show the largest regression weights for the mechanical objective. By contrast, the fluidic objective displays smaller and more scattered SRC values, again indicating a more complex, multi-parameter dependency. The consistency between Pearson, Spearman, and SRC analyses increases confidence in the robustness of the observed parameter rankings.

Global sensitivity metrics such as Sobol indices were not computed, as they require thousands of uniformly distributed samples across the full eight-dimensional design space, computationally prohibitive given that each evaluation involves expensive CFD and FEA. Moreover, because MOBO adaptively samples regions near the Pareto set, the resulting dataset reflects local sensitivity structure, not uniform global variation. This adaptive sampling can cause correlations to appear stronger or more directionally aligned than they would under global uniform sampling.

Despite the largely negative correlations for mechanical performance, simultaneously maximizing all geometric parameters does not yield a Pareto-optimal design. This underscores the inherently non-monotonic and genuinely multi-objective nature of the problem: improving mechanical strength often increases blockage and thereby deteriorates fluidic performance. The moderate cross-correlation between the two objectives (≈0.25–0.27) confirms that they are partially competing but not redundant, reinforcing the appropriateness of a multi-objective Bayesian optimization approach.

Overall, the sensitivity analysis shows that mechanical performance is governed by classical stiffness-driving parameters, while fluidic performance arises from multivariate, shape-dependent flow phenomena not dominated by any single geometric feature.

Flow structure and wake dynamics

To interpret the results, we post-processed the 3D flow data using ParaView. Flow structures were visualized by extracting iso-surfaces and planar slices of velocity and vorticity fields. We also examined cross-sectional slices through the lattice to see how the flow penetrates the interior. Supplementary Fig. 12 shows a representative vertical slice (through the center axis) of the vorticity field for the solid cylinder and the optimized designs A, D, H, and I at Reynolds number

Re ≈ 2100, corresponding to experimental SPIV conditions. These cross-sections reveal design-specific flow behavior, highlighting differences in how each structure guides the fluid.

The optimized structures show a clear suppression of early vortex shedding compared to the solid cylinder. Vortical structures are weaker, more dispersed, and originate further downstream, resulting in reduced wake unsteadiness and more stable flow patterns. In contrast, the solid cylinder generates strong, coherent vortices immediately behind its surface, typical of classical bluff body flow at this Reynolds number.

Supplementary Fig. 13 further illustrates these differences using horizontal mid-plane slices of vorticity magnitude. These top-down views capture the spatial organization of the wake, showing how the optimized designs alter the strength, position, and coherence of shed vortices. Flow through the porous networks is visible, and the onset of vortex shedding is consistently delayed relative to the solid cylinder.

To complement these static visualizations, we provide Supplementary Movies 6 and 7 showing time-resolved vorticity fields for all five designs. These movies offer a dynamic view of the vortex formation and shedding processes, illustrating the reduced and delayed vorticity evolution in the optimized structures. The complete code, along with documentation and execution scripts, is available at https://github.com/cselab/sponge and has been archived on Zenodo with a permanent DOI: https://doi.org/10.5281/zenodo.19136207.

CFD–SPIV comparison of wake dynamics

To directly compare experimental and simulated flow fields, we extracted the corresponding velocity data from the CFD simulations within the same spatial domain as the SPIV measurement window. This enabled side-by-side visual comparisons and allowed for direct overlays of experimental and numerical results, facilitating validation and deeper interpretation of the wake dynamics behind each design.

Supplementary Fig. 14 shows representative velocity field comparisons for the four optimized designs (A, D, H, and I). In each panel, velocity magnitude is shown as the background color, with vectors and streamlines overlaid to illustrate local flow direction and structure. The flow direction is upward, corresponding to the positive Y axis. Supplementary Movies 8–11 provide time-resolved comparisons between SPIV and CFD velocity fields, offering a dynamic view of wake evolution and vortex shedding.

Overall, velocity magnitudes match well in both spatial distribution and amplitude, confirming that the simulations capture the dominant flow structures and bulk transport observed experimentally. For all four designs, the extent and shape of the wake, as well as streamline orientations, are consistent across both datasets. However, some differences become apparent upon inspection of the time-resolved velocity field movies. In particular, vortex shedding appears more pronounced in the SPIV measurements, with vortices forming and detaching approximately 1 - 1.5-cylinder diameters closer to the structure compared to the CFD results. In the simulations, the onset of shedding is further downstream and, in some cases, partially outside the SPIV field of view. This discrepancy can be attributed to several factors inherent to experimental conditions: surface roughness introduced during sample fabrication may trigger earlier flow separation in physical tests; the finite size of the seeding particles (5–25 µm) may restrict their ability to fully enter and track flow within smaller pores, which were as small as 150 µm. Furthermore, near-wall boundary layers were more pronounced in the SPIV setup due to interaction with the tunnel walls, whereas the CFD simulations used periodic boundary conditions on the side walls to reduce

computational expense as previously described. Despite these differences, the overall dynamics and flow trends are well captured.

To complement the instantaneous snapshots in Supplementary Fig. 14, we include side-by-side comparisons of flow statistics aggregated over time for the same spatial domain and designs (A, D, H, and I). Supplementary Fig. 15 shows time-averaged velocity magnitude fields from CFD (left) and SPIV (right), using the same color scheme and spatial domain as Supplementary Fig. 14. The mean-flow fields agree in wake shape and width across the designs. In the SPIV data, the more fluidic-optimized designs (H and I) show vortex formation beginning further downstream compared to the mechanically optimized designs (A and D). This delayed vortex organization is consistent with the reduced force fluctuations of designs H and I reported in Fig. 5 of the main manuscript. In the CFD results, the wake extends further downstream, with part of the coherent structures lying outside the plotted field of view.

Supplementary Fig. 16 presents maps of turbulence kinetic energy (TKE) for the four optimized designs between CFD and SPIV. High-TKE regions correspond to shear layers and the vortex street. In the solid-cylinder wake, time-averaged TKE was about 50% higher and peaked roughly one diameter closer to the body compared to the optimized designs. CFD predicted an even more pronounced downstream shift than observed in SPIV.

To further assess agreement between simulation and experiment, Supplementary Fig. 17 provides quantitative comparisons of two wake metrics across all designs. Supplementary Fig. 17a shows the dominant vortex shedding frequencies obtained from FFT analysis of the velocity signals in the wake. For CFD, including the solid cylinder, the peak frequencies lie between 0.85 and 0.95 Hz. The corresponding SPIV peaks fall slightly higher, between 1.00 and 1.10 Hz. These values correspond to Strouhal numbers ranging from 0.20 (lowest in CFD) to 0.25 (highest in SPIV). The observed shift is within expectations when considering that: (i) the SPIV sampling rate results in a frequency bin width of 0.1 Hz, which limits precision in peak detection; (ii) small differences in the effective reference velocity and diameter between CFD and experiment can shift the calculated Strouhal number; and (iii) physical effects such as blockage, wall interactions, and geometric imperfections in the fabricated samples can promote earlier vortex formation and slightly higher shedding frequencies in SPIV.

Supplementary Fig. 17b compares the mean transverse velocity component in the wake, denoted v_x. Here we use the coordinate system introduced in Supplementary Fig. 14, where the bulk flow direction is upward along $+y$ and the transverse (lift) direction is along x. The comparison region is centered on the structure in x and located downstream in y, where vorticity is strongest. SPIV measurements report slightly elevated v_x values, on average 0.011 m s^{-1} higher than CFD. This is consistent with the more pronounced vortex shedding observed in the SPIV movies and can be explained by real-world effects such as tunnel wall influence, imperfect structural symmetry, or surface roughness from fabrication. In addition, the seeding particles used for SPIV (5 – 25 µm) are relatively large compared to the smallest lattice pores (~ 150 µm), and the bigger particles may not fully follow global flow within these regions. Such effects could bias local velocity measurements and contribute to the slightly stronger vortex shedding observed in SPIV relative to CFD. Importantly, relative to the solid cylinder, both CFD and SPIV show that the optimized designs reduce the transverse wake velocity v_x by a factor of roughly 3–5. This suppression of wake dynamics highlights the effectiveness of the design optimization process in reducing unsteady forces and enhancing flow stability.

Supplementary Tables

Supplementary Table 1: Comparison of Simulated and Experimental Mechanical Performance for Fabricated Designs. Axial stiffness (k, N mm^{-1}) and critical buckling load (P_{CR}, N) are reported for the four optimized lattice designs (A, D, H, and I). Finite element analysis (FEA) values were obtained from nonlinear simulations under axial compression. Experimental values are reported as mean ± standard deviation based on $n = 4$ specimens per design. Good agreement between simulation and experiment validates the predictive accuracy of the computational framework.

Optimum Design	Axial Stiffness [N mm^{-1}]		Critical Buckling Load [N]	
	Nonlinear FEA	Experiments	Nonlinear FEA	Experiments
A	405	423 ± 15	1505	1276 ± 21
D	420	420 ± 55	970	1075 ± 121
H	303	319 ± 22	502	608 ± 70
I	330	341 ± 27	660	634 ±78

Supplementary Table 2: Design parameters and performance metrics of Pareto-optimal solutions. Summary of all 12 non-dominated designs (A–L) identified from the multi-objective optimization. Columns include the design ID, eight geometric parameters defining the lattice architecture (see Methods), critical buckling load (P_{CR}, N), total structure volume (V, mm^3), mechanical cost (F_{mech}), and fluidic cost (F_{Fluid}). Both cost functions are minimized and represent normalized objective metrics quantifying structural performance (buckling resistance) and hydrodynamic behavior (e.g., drag and vortex shedding), respectively. Together, the listed designs span the trade-off between mechanical robustness and fluid-dynamic efficiency.

Optimum Design	Design Parameters								Results			
	N_V	N_C	W [mm]	H [mm]	N_L	R_H [mm]	N_{CW}	N_{CCW}	Buckling Load [N]	Volume [mm^3]	FEA Cost	CFD Cost
A	50	73	0.62	0.64	6	1.28	3	3	1638.7	12959	-2.83	0.32
B	47	57	0.64	0.64	4	1.37	3	3	1370.7	11003	-2.76	-0.63
C	49	56	0.63	0.64	4	1.54	3	0	1250.7	10173	-2.7	-0.93
D	49	57	0.64	0.65	5	1.34	1	1	1187.4	9858	-2.61	-1.04
E	48	62	0.59	0.65	4	1.58	0	3	1147.6	10019	-2.39	-1.06
F	48	67	0.56	0.63	3	1.36	0	3	1007.6	9076	-2.26	-1.06
G	46	69	0.63	0.64	3	1.42	1	0	1019.3	9544	-2.1	-1.07
H	34	30	0.58	0.65	2	1.40	1	0	538.1	5294	-1.91	-1.07
I	42	34	0.58	0.58	6	1.40	1	0	606.3	6118	-1.82	-1.12
J	50	53	0.60	0.63	1	1.60	0	1	843.9	8714	-1.73	-1.14
K	20	30	0.15	0.15	1	1.60	3	0	1.32	950	1.79	-1.15
L	20	30	0.15	0.15	6	1.60	0	3	1.95	2786	1.82	-1.21

Supplementary Figures

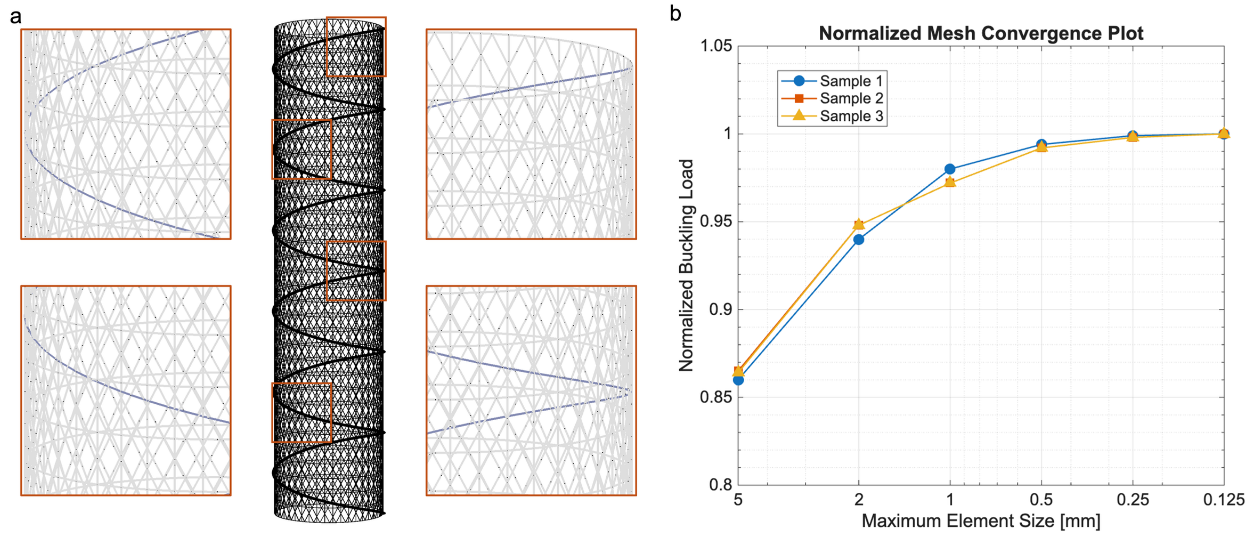

Supplementary Fig. 1: Mesh discretization and FEA convergence study for lattice buckling analysis. **a** Representative finite-element discretization of the *Euplectella*-inspired lattice structure with multiple detailed zoomed-in views, shown as line geometry with nodal points indicated as black dots. A maximum element length of 1 mm was applied along all beam segments to ensure consistent discretization; however, due to geometric intersections that form structural nodes, many beam segments, and thus finite elements, are shorter than this nominal maximum. For visualization clarity, elements are not explicitly shown. **b** Mesh-convergence plot of the normalized critical buckling load, obtained from linear eigenvalue buckling analyses for three representative designs. The mesh was progressively refined by decreasing the maximum element length from 5 mm down to 0.125 mm, and the predicted buckling loads were normalized by the value at the finest resolution. The results show that the buckling load approaches a stable value as the mesh is refined. A 1 mm maximum element length was selected for all simulations, yielding deviations below 3% relative to the finest mesh while substantially reducing computational cost. Source data are provided as a Source Data file.

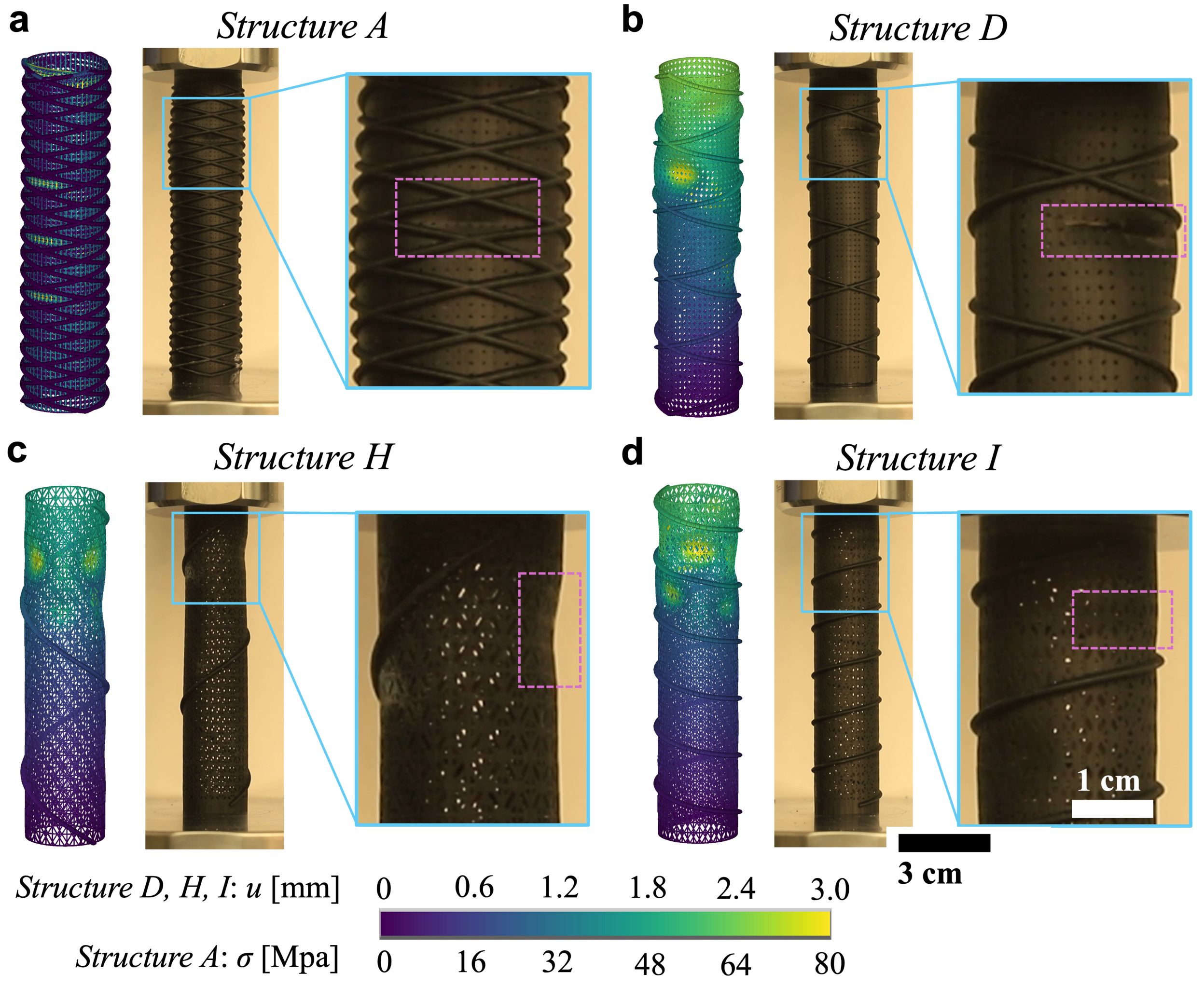

Supplementary Fig. 2: Comparison of nonlinear finite element predictions and experimental failure observations. a–d Four fabricated sponge-inspired lattice designs (A, D, H, and I) under quasi-static uniaxial compression. For each design, nonlinear finite element analysis (FEA) results are shown on the left, displaying either displacement magnitude (u, mm) or equivalent (von Mises) stress (σ, MPa), alongside corresponding experimental snapshots from compression tests on the right. Insets in the experimental images highlight the locations of initial buckling or localized failure. Colorbars indicate the magnitude of the plotted field in each simulation. Scale bars are shown in representative panels; all main images and all insets share consistent scales, respectively. Regions of maximum displacement or stress in the simulations closely coincide with experimentally observed failure locations, demonstrating strong agreement between numerical predictions and physical behavior.

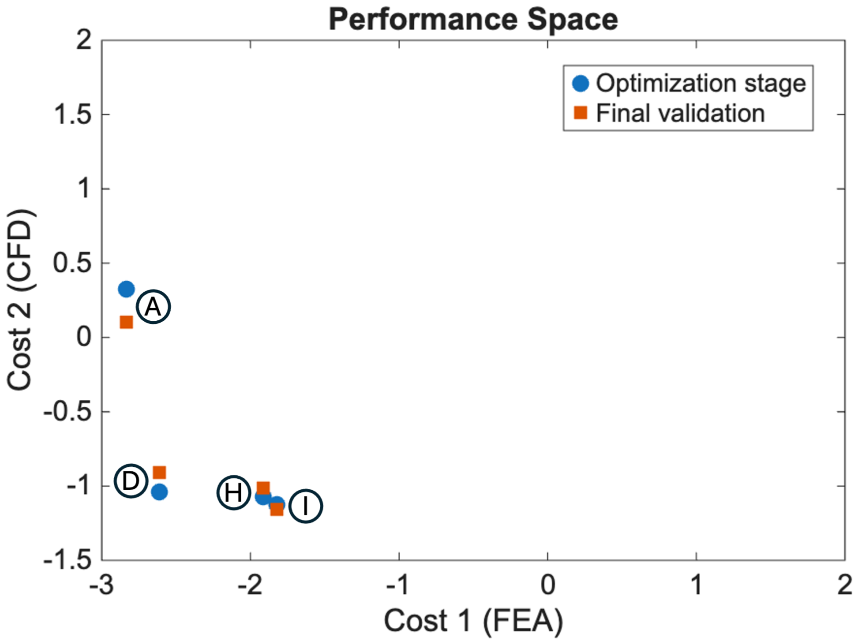

Supplementary Fig. 3: Comparison of optimization-stage and final high-resolution cost-function evaluations. Performance-space comparison for four Pareto-optimal designs (A, D, H, and I). Blue circles denote cost-function values obtained during the multi-objective Bayesian optimization (MOBO) stage using exploratory-resolution adaptive mesh refinement (AMR) simulations, while orange squares indicate the corresponding final high-resolution evaluations. The mechanical cost (F_{mech}) and fluidic cost (F_{fluid}) are shown (both minimized), representing normalized objective metrics associated with structural performance and hydrodynamic response, respectively. The ordering and relative spacing of designs are preserved between resolutions, and deviations are small, demonstrating that the lower-cost exploratory simulations accurately capture the global performance trends required for surrogate-model construction and design selection. Source data are provided as a Source Data file.

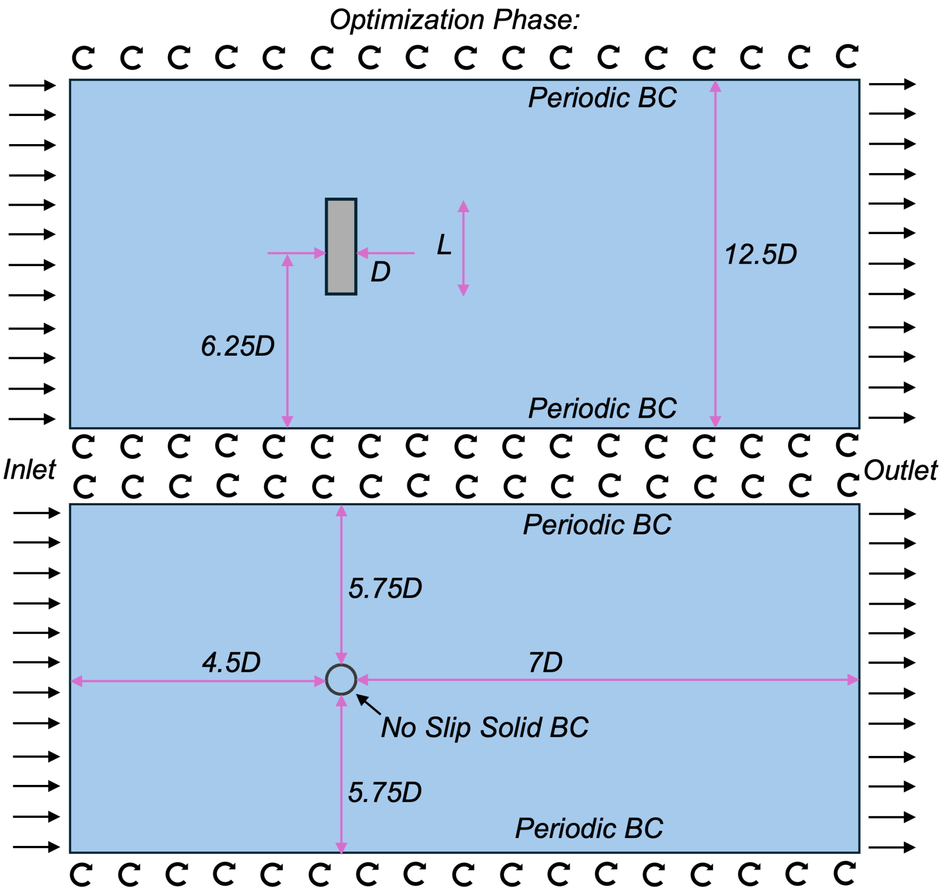

Supplementary Fig. 4: Computational domain and boundary conditions used during optimization-stage CFD simulations. Schematic of the fluid domain and boundary conditions employed during the optimization phase. A steady inflow velocity is prescribed at the inlet (left boundary), and an outlet boundary condition is applied downstream (right boundary). Periodic boundary conditions are imposed on all remaining domain boundaries (top, bottom, lateral directions, and along the cylinder axis), effectively approximating an unbounded flow domain while minimizing computational cost. The figure indicates all relevant domain dimensions. Simulations are performed at Reynolds number Re $\approx$ 2100. This configuration enables efficient evaluation of hydrodynamic performance during the multi-objective Bayesian optimization (MOBO) stage.

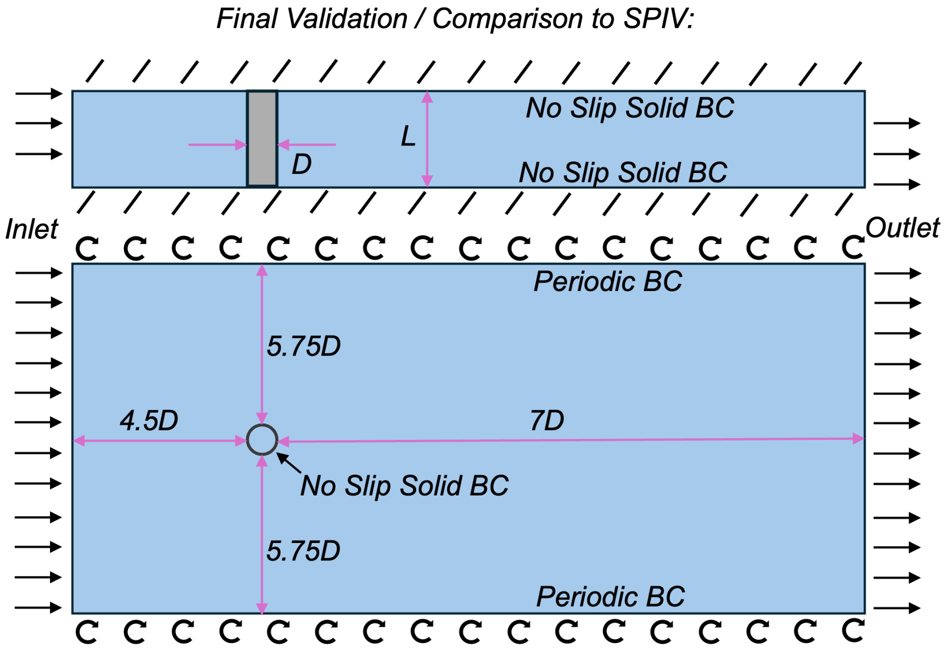

Supplementary Fig. 5: Computational domain and boundary conditions for high-fidelity CFD validation simulations. Schematic of the fluid domain and boundary conditions used for final validation simulations and comparison with experimental stereo particle image velocimetry (SPIV) measurements. A uniform inflow velocity is prescribed at the inlet (left boundary), and an outflow boundary condition with zero-pressure-gradient (Neumann condition) is applied downstream (right boundary). The top and bottom boundaries are modeled as no-slip walls, representing a confined channel. Periodic boundary conditions are applied in the lateral direction to reduce computational cost. No-slip boundary conditions are imposed on all solid surfaces of the cylinder and lattice structures. The figure indicates the relevant domain dimensions. Simulations are performed at Reynolds number Re ≈ 2100.

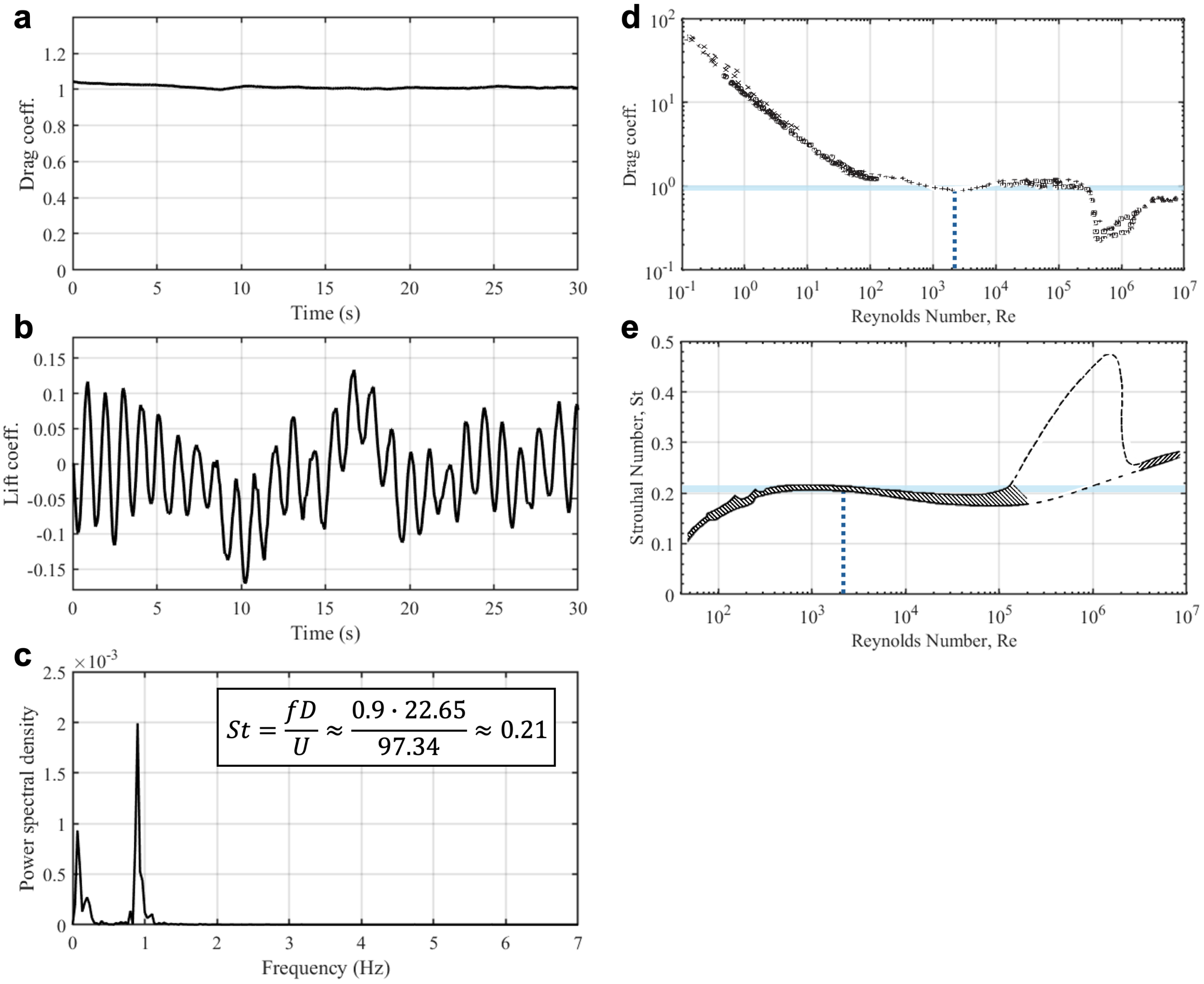

Supplementary Fig. 6: Hydrodynamic validation of flow around a circular cylinder at Reynolds number Re ≈ 2100. **a** Time-resolved drag coefficient after reaching quasi-steady-state conditions. **b** Corresponding lift coefficient showing periodic vortex shedding. **c** Power spectral density of the lift coefficient, indicating a dominant shedding frequency around 0.9 Hz, from which a Strouhal number of St≈0.21 is calculated. **d** Recreated plot of drag coefficient versus Reynolds number from Panton[13], showing close agreement with the simulated time-averaged drag coefficient ($c_D \approx 0.99$). **e** Recreated plot of Strouhal number versus Reynolds number for circular cylinders from Lienhard[12], confirming strong agreement with the computed value. Together, these results validate the accuracy of the simulation framework and establish a reliable baseline for assessing the performance of optimized porous designs. Source data are provided as a Source Data file.

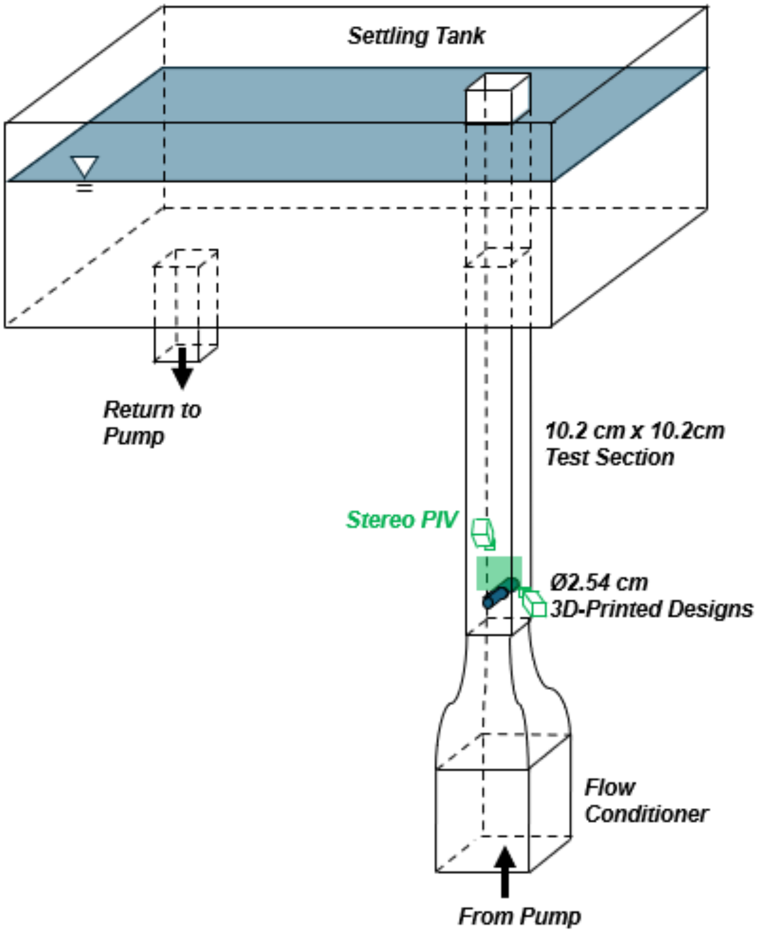

Supplementary Fig. 7: Schematic of the vertical flow loop and stereo particle image velocimetry (SPIV) system. Schematic illustration of the experimental setup used for flow measurements. The system consists of a vertical recirculating water tunnel including a flow conditioner and contraction section to provide a uniform inlet velocity profile, followed by a test section containing the sample. A dual-cavity Nd:YAG laser generates a planar light sheet intersecting the wake region downstream of the structure. Two cameras arranged in a stereoscopic configuration capture tracer particle motion for three-component velocity reconstruction in a planar measurement region. The figure highlights the relative arrangement of the flow loop, illumination system, and imaging setup used for quantitative comparison with computational fluid dynamics (CFD) simulations.

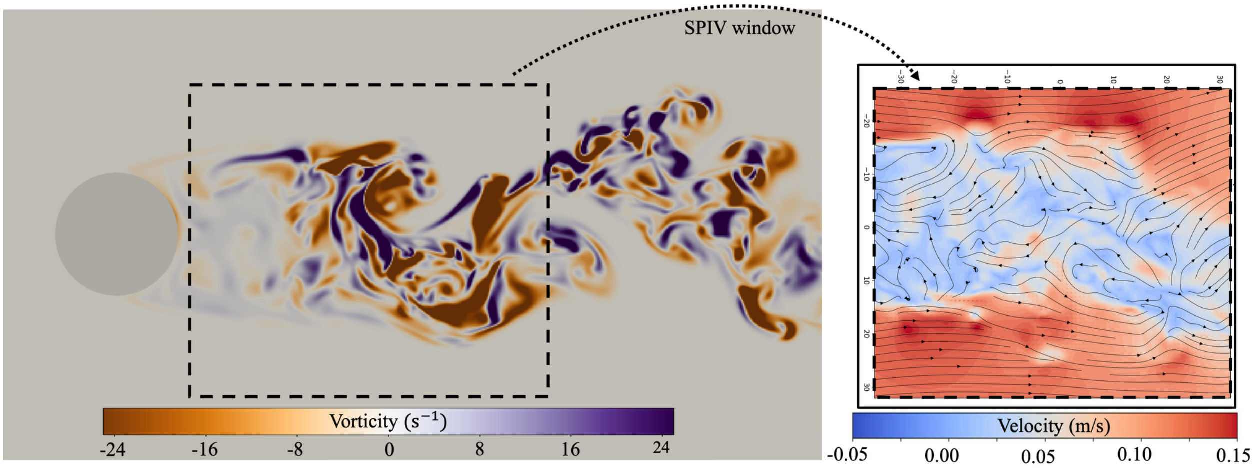

Supplementary Fig. 8: Definition of the SPIV measurement domain and representative flow-field visualization. Flow direction is from left to right. The left panel shows a representative computational fluid dynamics (CFD) snapshot of flow around a solid cylinder, with the stereoscopic particle image velocimetry (SPIV) measurement region indicated by a dashed box. The background color represents the spanwise vorticity. The right panel presents an example of the experimentally reconstructed velocity field within this domain, including streamlines. The background color represents the velocity magnitude, and arrows indicate the in-plane velocity direction. This figure illustrates the spatial extent and type of flow data used for comparison between experiments and simulations.

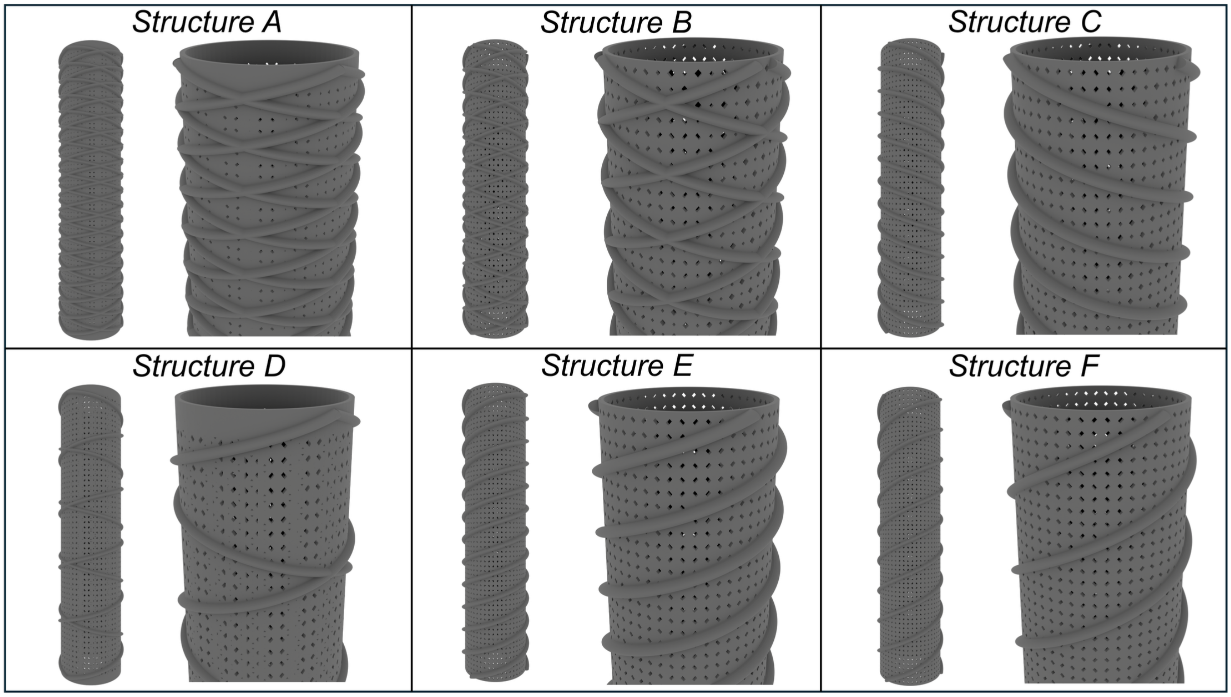

Supplementary Fig. 9: Three-dimensional renderings of Pareto-optimal lattice designs A–F. Three-dimensional visualizations of six non-dominated designs (A–F) identified from the multi-objective Bayesian optimization (MOBO). The geometries illustrate variations in the underlying lattice architecture arising from trade-offs between mechanical robustness and hydrodynamic efficiency, as quantified by the mechanical cost and fluidic cost (both minimized).

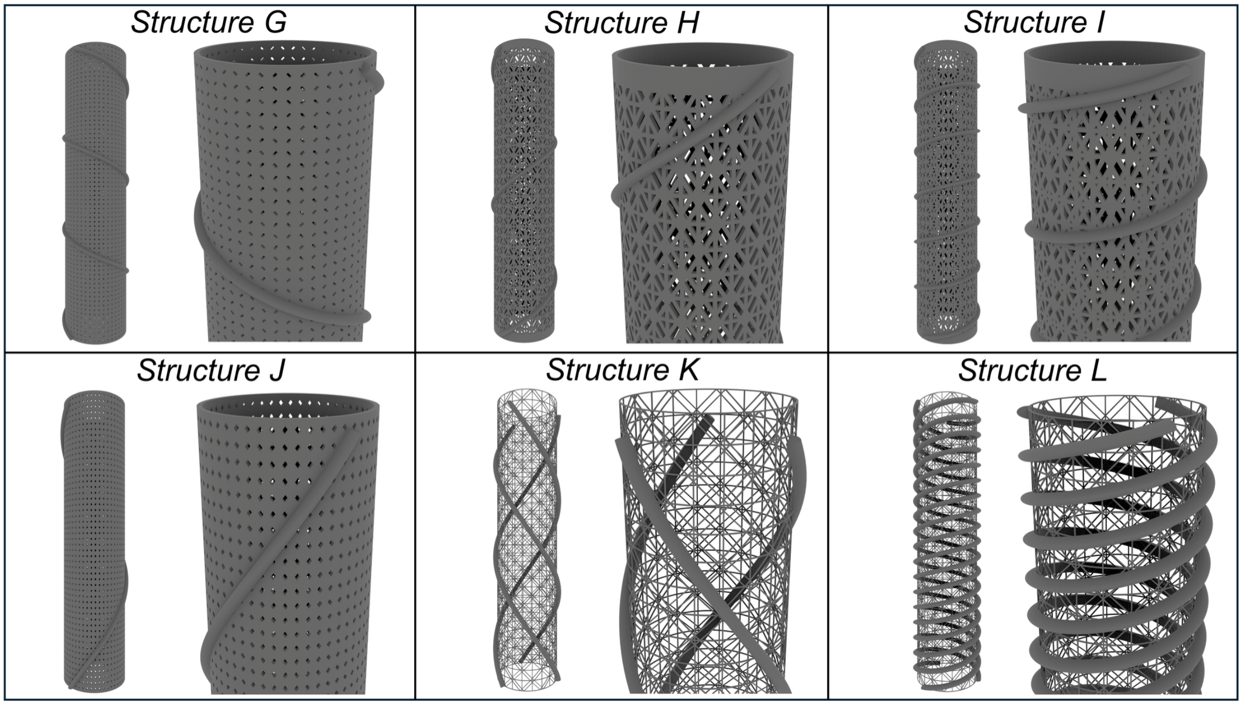

Supplementary Fig. 10: Three-dimensional renderings of Pareto-optimal lattice designs G-L. Three-dimensional visualizations of six non-dominated designs (G–L) identified from the multi-objective Bayesian optimization (MOBO). The geometries illustrate variations in the underlying lattice architecture arising from trade-offs between mechanical robustness and hydrodynamic efficiency, as quantified by the mechanical cost and fluidic cost (both minimized).

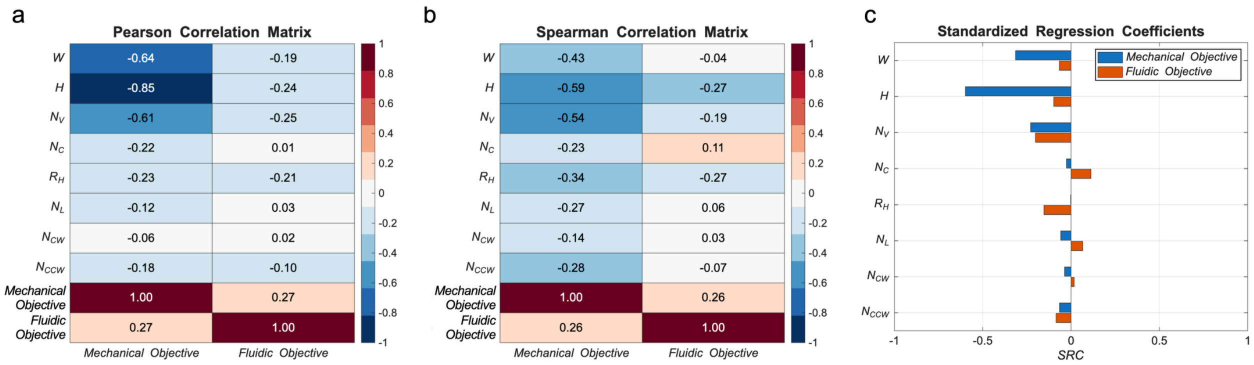

Supplementary Fig. 11: Sensitivity analysis of geometric parameters in the multi-objective Bayesian optimization (MOBO) framework. a Pearson correlation matrix showing linear correlations between geometric design parameters and the mechanical and fluidic cost functions. **b** Spearman correlation matrix showing rank-based (monotonic) relationships between parameters and objectives. **c** Box plots of standardized regression coefficients (SRC) obtained from bootstrapped linear regression models, quantifying parameter importance and variability. Across all three metrics, the beam cross-section dimensions (*W*, width; *H*, height) and the number of vertical beams (N_v) exhibit the strongest influence on the mechanical objective, while the fluidic objective shows weaker and more distributed sensitivities across multiple parameters. Correlation values are predominantly negative for the mechanical cost due to its definition (lower values correspond to improved mechanical performance). The consistency across Pearson, Spearman, and SRC analyses indicates robust parameter–objective relationships within the sampled design space. Source data are provided as a Source Data file.

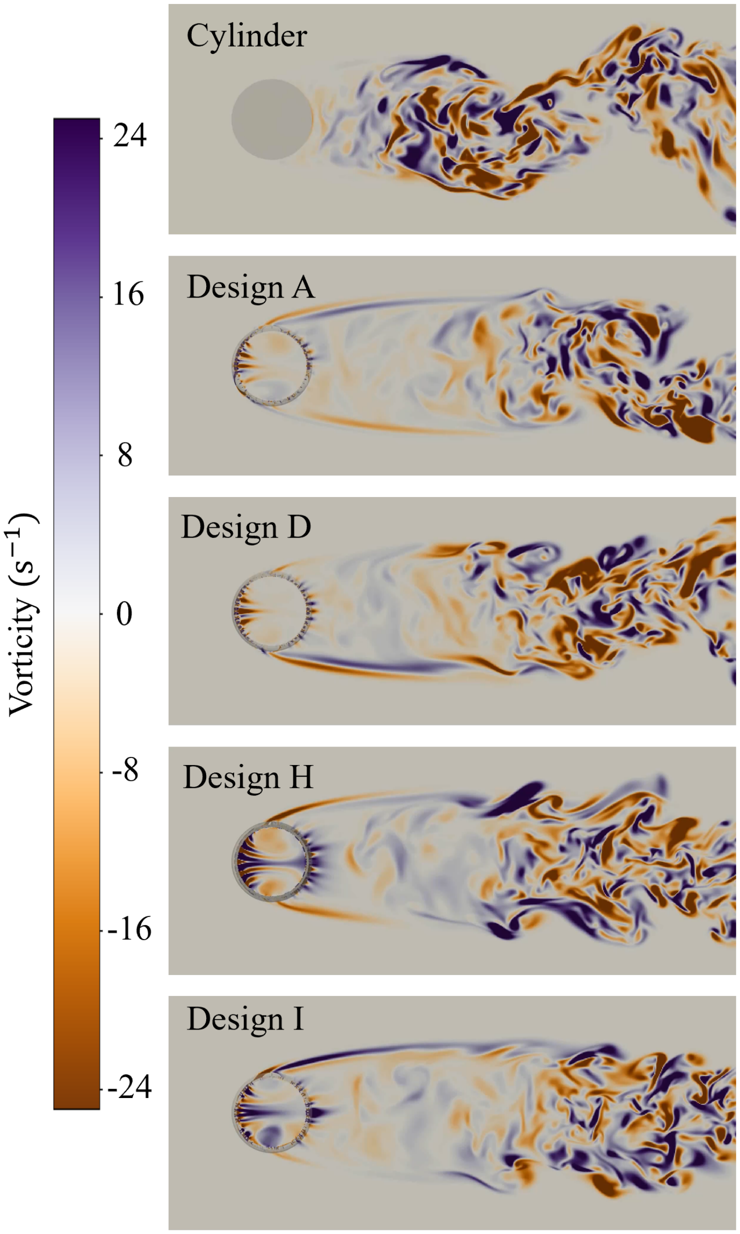

Supplementary Fig. 12: Cross-sectional vorticity fields from computational fluid dynamics (CFD) simulations for the solid cylinder and optimized lattice designs. Vertical center-plane slices of the flow field for a solid cylinder and sponge-inspired designs (A, D, H, and I) at Reynolds number Re ≈ 2100, corresponding to experimental stereo particle image velocimetry (SPIV) conditions. The background color represents the vorticity. These cross-sections reveal how the porous architectures modify near-field flow behavior: the optimized designs promote flow penetration through the structure and suppress the formation of strong near-wake vortices, displacing vorticity generation further downstream compared to the solid cylinder.

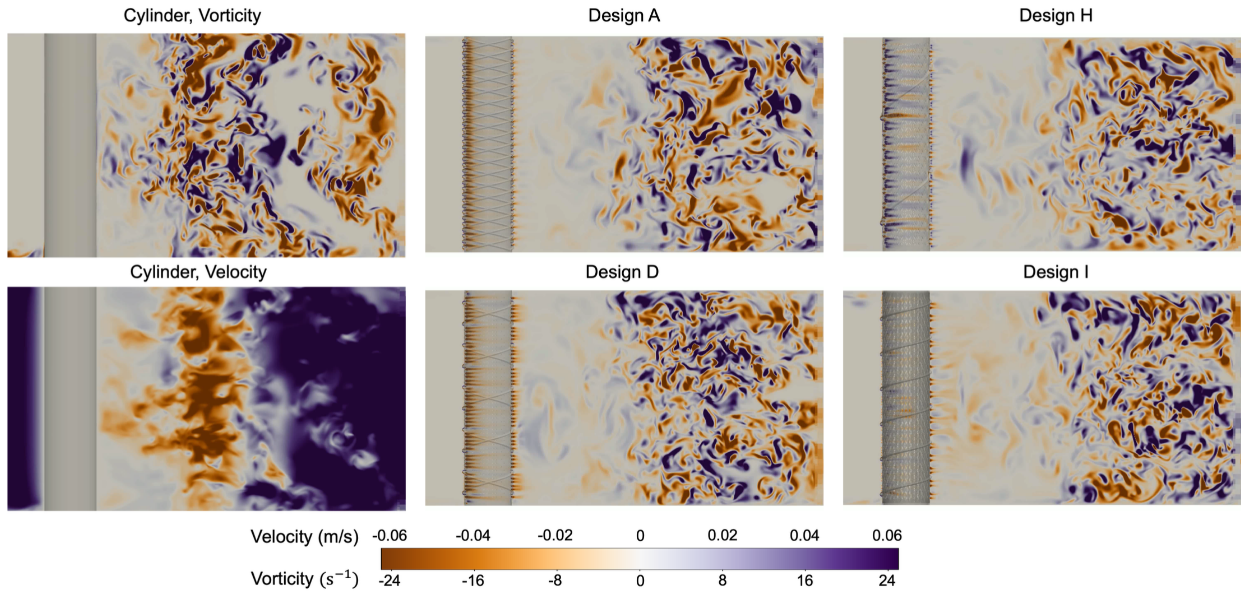

Supplementary Fig. 13: Top-down vorticity fields from computational fluid dynamics (CFD) simulations for the solid cylinder and optimized designs. Horizontal mid-plane slices of the flow field obtained from computational fluid dynamics (CFD) simulations for a solid cylinder and sponge-inspired designs (A, D, H, and I) at Reynolds number Re ≈ 2100. The background color represents the spanwise velocity and vorticity. The porous designs exhibit flow-through behavior and delayed vortex shedding, with weaker and more downstream-displaced vortical structures compared to the strong, coherent near-wake vortices generated by the solid cylinder.

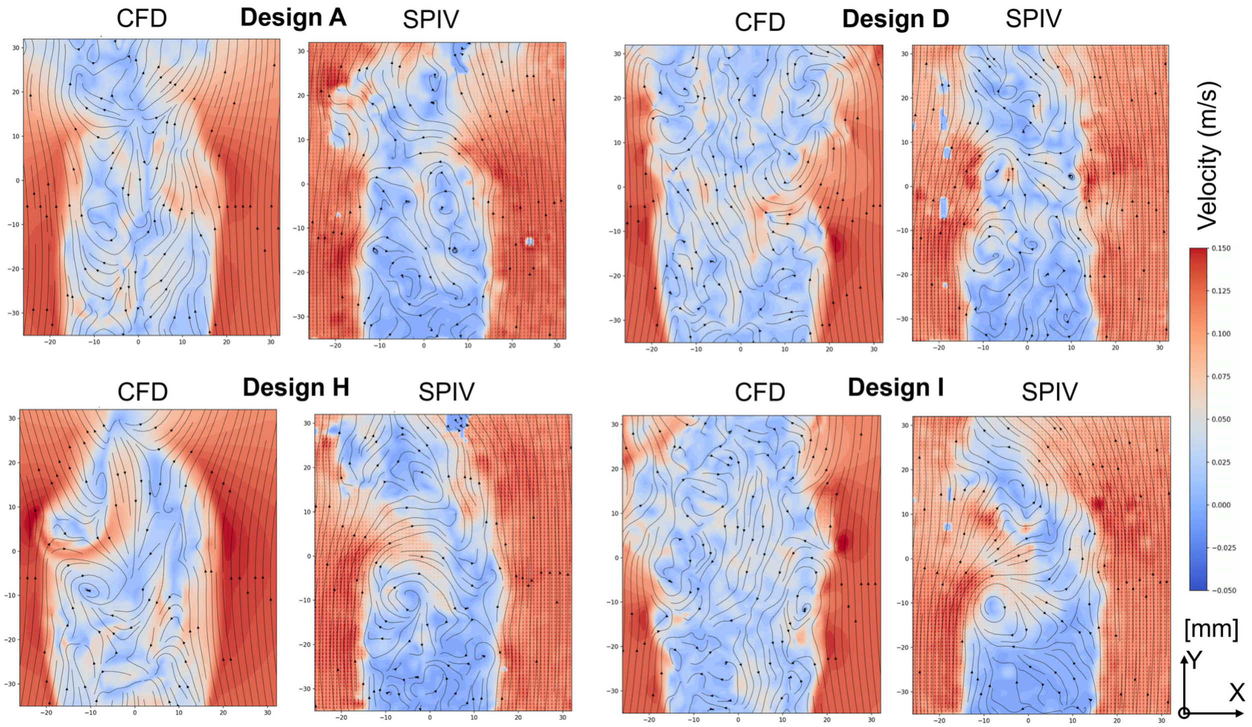

Supplementary Fig. 14: Time-matched velocity fields from CFD and SPIV for optimized designs. Side-by-side comparison of velocity fields obtained from computational fluid dynamics (CFD, left) and stereo particle image velocimetry (SPIV, right) for optimized designs A, D, H, and I at Reynolds number Re ≈ 2100. The flow is directed upward along the positive y-axis, while transverse (lift) velocities act along the x-axis. Each panel shows the same spatial domain. The background color represents the velocity

magnitude, with vectors and streamlines overlaid to indicate local flow direction and structure. The spatial distribution and magnitude of the velocity field are in good agreement between CFD and SPIV, demonstrating that the simulations capture the dominant wake features observed experimentally. Time-resolved comparisons are provided in Supplementary Movies 8–11.

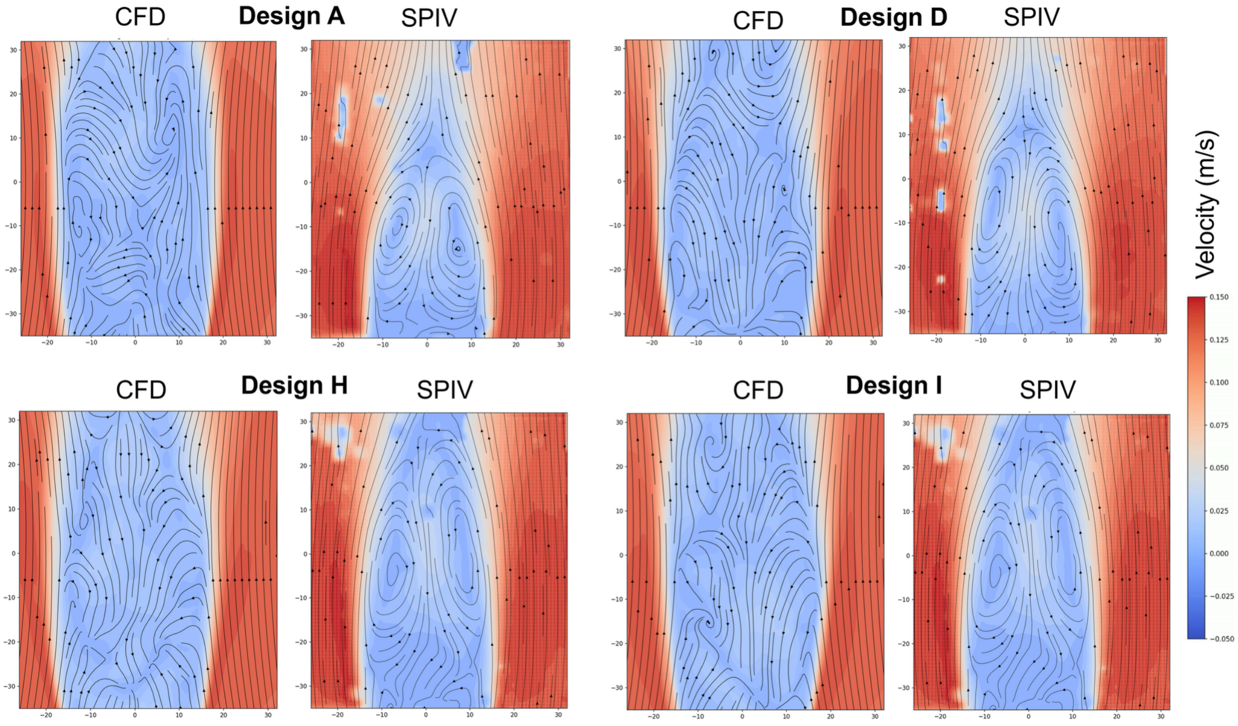

Supplementary Fig. 15: **Time-averaged velocity fields from CFD and SPIV for optimized designs.** Side-by-side comparison of time-averaged velocity fields obtained from computational fluid dynamics (CFD, left) and stereo particle image velocimetry (SPIV, right) for optimized designs A, D, H, and I at Reynolds number Re $\approx$ 2100. The flow is directed upward along the positive y-axis, while transverse (lift) velocities act along the x-axis. Each panel shows the same spatial domain. The background color represents the time-averaged velocity magnitude. The results show good agreement between CFD and SPIV in the spatial distribution of the mean flow field, confirming that the simulations capture the time-averaged wake structure of the optimized designs.

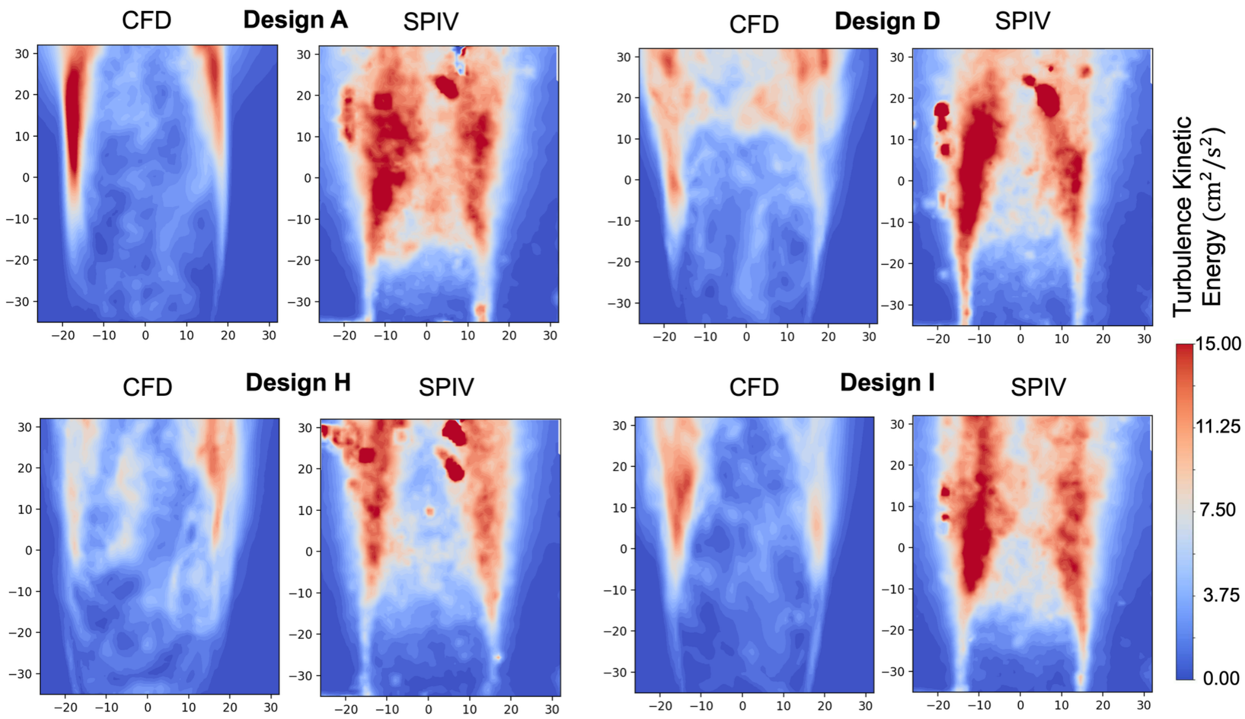

Supplementary Fig. 16: Turbulent kinetic energy (TKE) fields from CFD and SPIV for optimized designs. Side-by-side comparison of turbulent kinetic energy (TKE) fields obtained from computational fluid dynamics (CFD, left) and stereo particle image velocimetry (SPIV, right) for optimized designs A, D, H, and I at Reynolds number Re ≈ 2100. Each panel shows the same spatial domain. The background color represents the turbulent kinetic energy. High-energy regions correspond to shear layers and vortex shedding in the wake. Compared to a solid cylinder baseline, the optimized designs exhibit reduced and downstream-shifted TKE, indicating suppressed near-field turbulence generation. CFD and SPIV show consistent spatial trends, with CFD predicting a slightly more pronounced downstream shift of peak TKE.

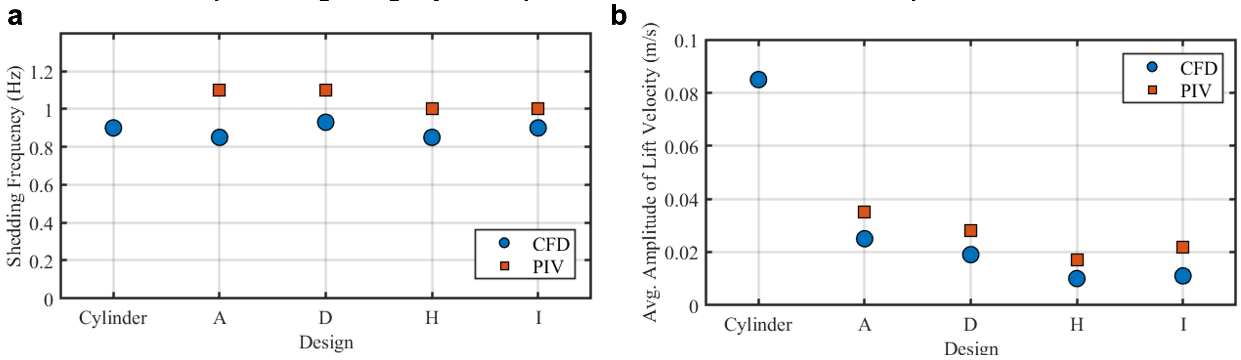

Supplementary Fig. 17: Quantitative comparison of wake dynamics between CFD and SPIV. **a** Dominant vortex shedding frequency extracted from the wake region for the solid cylinder and optimized designs (A, D, H, and I). Frequencies are obtained from power spectral analysis of the velocity signal. CFD results range from 0.85–0.95 Hz, while SPIV results range from 1.00–1.10 Hz, corresponding to Strouhal numbers $St \approx 0.20$–0.25. **b** Mean transverse velocity component (v_x,) in the downstream wake region of maximum vorticity (coordinate system as defined in Supplementary Fig. 14, with flow along $+y$ and transverse direction along x). SPIV values are slightly higher than CFD, likely due to surface roughness of fabricated samples, particle tracking limitations, and boundary effects. Both datasets, however, show that the optimized designs reduce transverse wake velocities by a factor of $\sim 3-5$ compared to the solid cylinder, demonstrating the effectiveness of the optimized geometries in suppressing wake instabilities. Source data are provided as a Source Data file.

Supplementary References

1. Bradford, E., Schweidtmann, A. M. & Lapkin, A. Efficient multiobjective optimization employing Gaussian processes, spectral sampling and a genetic algorithm. *J Glob Optim* **71**, 407–438 (2018).

2. Helton, J. C. & Davis, F. J. Latin hypercube sampling and the propagation of uncertainty in analyses of complex systems. *Reliability Engineering & System Safety* **81**, 23–69 (2003).

3. Tian, Y., Luković, M. K., Erps, T., Foshey, M. & Matusik, W. AutoOED: Automated Optimal Experiment Design Platform. Preprint at https://doi.org/10.48550/ARXIV.2104.05959 (2021).

4. Ananthakrishnan, R., Chard, K., Foster, I. & Tuecke, S. Globus platform-as-a-service for collaborative science applications. *Concurrency and Computation* **27**, 290–305 (2015).

5. Teng, J. G., Rotter, J. M., Teng, J. G. & Rotter, J. M. *Buckling of Thin Metal Shells*. (Spon Press, London, 2004).

6. He, S., Jiang, Z. & Cai, J. Investigation on Simulation Methods of Initial Geometric Imperfection Distribution in Elasto-plastic Stability Analysis of Single-layer Reticulated Shells. *KSCE Journal of Civil Engineering* **22**, 1193–1202 (2018).

7. Popinet, S. An accurate adaptive solver for surface-tension-driven interfacial flows. *Journal of Computational Physics* **228**, 5838–5866 (2009).

8. Song, B. *et al.* Direct numerical simulation of flow over a cylinder immersed in the grid-generated turbulence. *Physics of Fluids* **34**, 015109 (2022).

9. Bandler, J. W., Biernacki, R. M., Shao Hua Chen, Grobelny, P. A. & Hemmers, R. H. Space mapping technique for electromagnetic optimization. *IEEE Trans. Microwave Theory Techn.* **42**, 2536–2544 (1994).

10. Wang, H., Jin, Y. & Doherty, J. A Generic Test Suite for Evolutionary Multifidelity Optimization. *IEEE Trans. Evol. Computat.* **22**, 836–850 (2018).

11. Van Rees, W. M., Gazzola, M. & Koumoutsakos, P. Optimal shapes for anguilliform swimmers at intermediate Reynolds numbers. *J. Fluid Mech.* **722**, R3 (2013).

12. Lienhard, J. H., Service., W. S. University. T. E. & of Engineering. Research Division., W. S. University. C. Synopsis of lift, drag, and vortex frequency data for rigid circular cylinders. (1966).

13. *Incompressible Flow*. (John Wiley and Sons, Inc, Hoboken, New Jersey, 2013).
