# Supplementary material for: Optimized mechano-fluidic metamaterials inspired by deep-sea sponges
Source: Nat Commun. 2026 May 5;17:6062. doi: 10.1038/s41467-026-72612-4 (PMC13350808; doi:10.1038/s41467-026-72612-4)
Supplement: Supplementary file 2 — Description of Additional Supplementary Files [file 41467_2026_72612_MOESM2_ESM.pdf]

Description of Supplementary Data and Multimedia Files for

# Optimized Mechano-Fluidic Metamaterials Inspired by Deep-Sea Sponges

Timon Meier *et al.*

Corresponding authors: Costas P. Grigoropoulos: cgrigoro@berkeley.edu,
Petros Koumoutsakos: petros@seas.harvard.edu

This document provides a brief description of the supplementary datasets, videos, and software files associated with this manuscript.

## Supplementary Movies

**Supplementary Movie 1 - Evolution of lattice designs during optimization**
Animation illustrating the evolution of lattice geometries throughout the multi-objective Bayesian optimization process. Each frame represents a candidate design evaluated during the optimization, showing how the algorithm explores the design space and converges toward Pareto-optimal solutions.

**Supplementary Movie 2 - Compression experiment of optimized Design A**
Video recordings of the uniaxial compression experiments performed on lattice Design A. The footage illustrates the deformation and failure behavior of the structure under increasing compressive load.

**Supplementary Movie 3 - Compression experiment of optimized Design D**
Video recordings of the uniaxial compression experiments performed on lattice Design D. The footage illustrates the deformation and failure behavior of the structure under increasing compressive load.

**Supplementary Movie 4 - Compression experiment of optimized Design H**
Video recordings of the uniaxial compression experiments performed on lattice Design H. The footage illustrates the deformation and failure behavior of the structure under increasing compressive load.

**Supplementary Movie 5 - Compression experiment of optimized Design I**
Video recordings of the uniaxial compression experiments performed on lattice Design I. The footage illustrates the deformation and failure behavior of the structure under increasing compressive load.

**Supplementary Movie 6 - CFD comparison of vorticity fields (Z-slice)**
Comparison of instantaneous vorticity fields extracted from CFD simulations on a vertical (Z-

direction) slice cross-section for the solid cylinder and the optimized lattice designs. The video highlights differences in vortex formation and wake development.

**Supplementary Movie 7 - CFD comparison of vorticity fields (Y-slice)**
Comparison of instantaneous vorticity fields extracted from CFD simulations on a horizontal (Y-direction) slice cross-section across the different lattice designs and reference cylinder.

**Supplementary Movie 8 - CFD vs. SPIV comparison for Design A**
Side-by-side comparison of flow fields between computational fluid dynamics (CFD) simulations and stereo particle image velocimetry (SPIV) measurements for lattice Design A.

**Supplementary Movie 9 - CFD vs. SPIV comparison for Design D**
Side-by-side comparison of flow fields between computational fluid dynamics (CFD) simulations and stereo particle image velocimetry (SPIV) measurements for lattice Design D.

**Supplementary Movie 10 - CFD vs. SPIV comparison for Design H**
Side-by-side comparison of flow fields between computational fluid dynamics (CFD) simulations and stereo particle image velocimetry (SPIV) measurements for lattice Design H.

**Supplementary Movie 11 - CFD vs. SPIV comparison for Design I**
Side-by-side comparison of flow fields between computational fluid dynamics (CFD) simulations and stereo particle image velocimetry (SPIV) measurements for lattice Design I.

---

## Supplementary Data

**Supplementary Data 1 - Optimization dataset of evaluated designs**
Excel spreadsheet containing the geometric design parameters and corresponding cost-function values for all lattice designs evaluated during the multi-objective Bayesian optimization process. The dataset includes the parameters defining the parametric lattice geometry as well as the associated mechanical and fluidic performance metrics.

---

## Supplementary Software

**Supplementary Software - Code for lattice generation and simulations**
Compressed archive containing Python scripts used to generate the parametric lattice geometries and export them as STL files, as well as scripts used to automate the Ansys finite element analysis (FEA) simulations performed in this study.
