# Supplementary material for: Optimized mechano-fluidic metamaterials inspired by deep-sea sponges
Source: Nat Commun. 2026 May 5;17:6062. doi: 10.1038/s41467-026-72612-4 (PMC13350808; doi:10.1038/s41467-026-72612-4)
Supplement: Supplementary file 15 — Supplementary Software [file 41467_2026_72612_MOESM15_ESM.zip › Supplementary-Software/CodeSTLGeneration/README.docx]

Install required libraries, updated desired parameters in main.py and run main.py with other .py files in the same folder. A STL model will be created with the specified parameters
