# Supplementary material for: Optimized mechano-fluidic metamaterials inspired by deep-sea sponges
Source: Nat Commun. 2026 May 5;17:6062. doi: 10.1038/s41467-026-72612-4 (PMC13350808; doi:10.1038/s41467-026-72612-4)
Supplement: Supplementary file 17 — Transparent Peer Review file [file 41467_2026_72612_MOESM17_ESM.pdf]

nature portfolio

Peer Review File

Optimized Mechano-Fluidic Metamaterials Inspired by Deep-Sea Sponges

Corresponding Author: Professor Costas Grigoropoulos

This file contains all reviewer reports in order by version, followed by all author rebuttals in order by version.

Version 0:

Reviewer comments:

Reviewer #1

(Remarks to the Author)
This work uses a surrogate model to map the design space based on a framework inspired by the deep-sea sponge Euplectella aspergillum. The authors employed CFD (for drag and lift) and buckling analysis (critical buckling force) as two competing objectives to perform Pareto-front optimization and explore trade-offs between mechanical and fluidic performance. They claim to have identified several designs that balance these two competing objectives and verified their findings using 3D-printed samples and Stereoscopic Particle Image Velocimetry (SPIV).

The analysis is elaborate and interesting. However, this type of sponge structure has been extensively studied and has served as a framework for various optimization studies since the early 2000s. The manuscript lacks sufficient discussion of how similar works were conducted and how the proposed design compares to typical natural geometries and previous findings. An Ashby-type comparison could help clarify the advantages of this design more...

My assessment is that, while the work has merit, its novelty is borderline for publication in Nature Communications. Without a stronger explanation of the mechanical analysis and a clearer justification of the use of the Volume of Fluid (VOF) method, there are weaknesses and several “fudged” details in how some aspects are presented. Using a coarse sweep to explore the design space is a concern, since the entire batching and surrogate framework hinges on that step. This could be acceptable if proper comparison is made with other optimization approaches for similar structures.

Another issue is that, since the surrogate (black-box) optimization framework is already implemented, it would be straightforward to perform a sensitivity analysis to determine which of the competing cost functions is most influential.

Having said that, the reviewer does not recommend this paper for Nature Communications in its current form, without major revision and clarification.

Major Concerns

Introduction, Lines 95–96:
“In contrast, MOBO is effective for optimizing costly black-box functions with a limited number of evaluations and can be parallelized on HPC resources.”

The authors should explicitly state that MOBO uses a probabilistic surrogate model.

Introduction, Lines 97–98:
“…balancing mechanical resilience and hydrodynamic performance.”

“Mechanical resilience” is too generic; specify the mechanical property (stiffness, compliance, strain energy density, or toughness).

Figure 1F:
The figure is poorly constructed; several applications overlap and are not clearly labeled.

Figure 1B (MOBO Flow):
The flow in Fig. 1B exposes a major weakness in the optimization framework. The design space was explored using “high-fidelity” simulations with coarse-mesh settings, as stated in the manuscript. The batch generation step relies entirely on the surrogate model trained during the MOBO stage. Therefore, any inaccuracies in the coarse evaluations will directly bias the selection process. Even though the algorithmic framework appears elaborate, the entire optimization outcome is limited by the accuracy of the MOBO surrogate model, which drives all subsequent design decisions.

Lines 158–159:
“During the MOBO stage, coarser AMR settings were applied…”

It is unclear what the mechanical cost function represents. The authors refer to “strength,” but this needs clarification.

Lines 177–178:
“The optimization produced a design space mapping the trade-offs between mechanical strength and fluidic efficiency.”

The fluidic cost function is described as “fluidic efficiency,” which is vague. Define this term precisely.

Lines 389–391:
“Linear buckling analysis (Analysis type: BUCKLE) to obtain the critical buckling force.”

Stating “Analysis type: BUCKLE” is not a scientific description of the method. Specify whether the buckling was captured using an eigenvalue-based approach or a transient nonlinear analysis.

Lines 395:
“…deformations (NLGEOM), and post-buckling behavior…”

NLGEOM enables large deformation effects but does not simulate imperfections. Explain how imperfections were introduced and how they relate to NLGEOM.

Lines 408:
“Using the Volume of Fluid (VOF) method…”

The authors must justify the use of VOF, which is intended for multiphase or immiscible fluid flows. If the flow is single-phase, VOF adds unnecessary complexity.

Lines 449–450:
“…strain rate of 0.1 s^{-1} until failure, indicated by buckling.”

Clarify whether this strain rate corresponds to a displacement rate that ensures quasi-static conditions. Buckling loads can depend strongly on the applied strain rate.

(Remarks on code availability)
Code was not reviewed.

Reviewer #2

(Remarks to the Author)
The paper is relevant to the Nature Communications journal. Originality of the work is derived from the multiphysics approach to investigating the Venus flower basket sponge. Technical accuracy and completeness are excellent. References to pertinent work in the field are comprehensive. A proper combination of comprehensiveness and conciseness is achieved well. The quality of presentation is also excellent.

• Note the typo: “sies”
• You could consider including some additional details for the FEA analysis, such as meshing strategies (there was no image of the mesh) and an accompanying mesh convergence study.

Conclusion: This is an extremely well-written paper both in its clarity and its attention to detail. I therefore recommend publication in its current form.

(Remarks on code availability)

Version 1:

Reviewer comments:

Reviewer #2

(Remarks to the Author)
The authors have satisfactorily addressed my earlier comments

(Remarks on code availability)

Open Access This Peer Review File is licensed under a Creative Commons Attribution 4.0 International License, which permits use, sharing, adaptation, distribution and reproduction in any medium or format, as long as you give appropriate credit to the original author(s) and the source, provide a link to the Creative Commons license, and indicate if changes were made.
In cases where reviewers are anonymous, credit should be given to 'Anonymous Referee' and the source.
The images or other third party material in this Peer Review File are included in the article's Creative Commons license, unless indicated otherwise in a credit line to the material. If material is not included in the article's Creative Commons license and your intended use is not permitted by statutory regulation or exceeds the permitted use, you will need to obtain permission directly from the copyright holder.
To view a copy of this license, visit https://creativecommons.org/licenses/by/4.0/

Authors' response to the reviewers' comments for: "**Metamaterials from the Deep: Optimized Mechano-Fluidic Materials Inspired by Deep-Sea Sponges**" by T. Meier et al.

The authors are grateful to the reviewers for their time and their constructive comments, which have helped us improve the revised manuscript.

A detailed point-by-point response to each comment is given below, along with the location (lines) in the revised manuscript where the corresponding revision was made. In addition, main revisions have been highlighted using yellow background in the revised text of the manuscript. (Review comments are shown in black font, whereas author responses are shown in blue font). Passages in underlined italics represent quotations from the revised manuscript. Relevant references are provided at the end of each addressed comment.

Reviewer #1

This work uses a surrogate model to map the design space based on a framework inspired by the deep-sea sponge *Euplectella aspergillum*. The authors employed CFD (for drag and lift) and buckling analysis (critical buckling force) as two competing objectives to perform Pareto-front optimization and explore trade-offs between mechanical and fluidic performance. They claim to have identified several designs that balance these two competing objectives and verified their findings using 3D-printed samples and Stereoscopic Particle Image Velocimetry (SPIV).

The analysis is elaborate and interesting. However, this type of sponge structure has been extensively studied and has served as a framework for various optimization studies since the early 2000s. The manuscript lacks sufficient discussion of how similar works were conducted and how the proposed design compares to typical natural geometries and previous findings. An Ashby-type comparison could help clarify the advantages of this design more...

My assessment is that, while the work has merit, its novelty is borderline for publication in Nature Communications. Without a stronger explanation of the mechanical analysis and a clearer justification of the use of the Volume of Fluid (VOF) method, there are weaknesses and several "fudged" details in how some aspects are presented. Using a coarse sweep to explore the design space is a concern, since the entire batching and surrogate framework hinges on that step. This could be acceptable if proper comparison is made with other optimization approaches for similar structures.

Another issue is that, since the surrogate (black-box) optimization framework is already implemented, it would be straightforward to perform a sensitivity analysis to determine which of the competing cost functions is most influential.

Having said that, the reviewer does not recommend this paper for Nature Communications in its current form, without major revision and clarification.

In response to the request for a more comprehensive discussion of prior work, we substantially expanded the Introduction to provide a structured and comprehensive overview of previous studies. We discuss how earlier works approached mechanical optimization, fluid-dynamic analysis, and specific geometric motifs in isolation. We also clarify how prior studies employed evolutionary algorithms, topology optimization, parametric sweeps, or surrogate models to enhance structural performance, and how fluid-dynamic investigations were typically conducted separately using simplified geometries or limited CFD case studies.

To the best of our knowledge, our study is the first to integrate *Euplectella*-inspired geometric features into a fully automated, multi-objective optimization framework that simultaneously accounts for both mechanical and fluid-dynamic performance. Previous investigations have predominantly examined mechanical behavior or flow characteristics separately, and none have systematically explored the coupled multiphysics trade-offs between the two within a unified optimization loop. Likewise, prior optimization studies on *Euplectella*-like structures have focused almost exclusively on mechanical objectives and have not incorporated large-scale, high-fidelity CFD evaluations across hundreds of candidate geometries. In contrast, our formulation jointly maximizes buckling resistance normalized by volume while minimizing drag, lift, and vortex-shedding intensity, the latter quantified through temporal fluctuations of the lift force.

A second novelty in the present paper is the experimental validation of the proposed computational framework. We fabricated multiple designs, suggested by the CFD and FEA, spanning the Pareto front and experimentally evaluated their performance using compression tests and SPIV measurements, thereby probing both mechanical stability and flow fields. The good agreement between simulations and experiments confirms the fidelity of the FEA and CFD models and establishes the proposed optimization workflow as a robust and extensible platform that can be applied to larger design spaces and other classes of architected materials.

Modified parts of the Introduction (Lines 60 - 119):

Since the early 2000s, studies have extensively explored the mechanical and fluidic aspects of this architecture, establishing E.a. as a long-standing framework for biomimetic benchmarks and optimization. Weaver et al.[12] demonstrated the structural integrity of its six-level hierarchical design, ranging from nanometers to centimeters, emphasizing its efficiency in material use and mechanical stability. Monn et al.[13,18] further showed that the individual sponge spicules exhibit high flexibility and optimal strength.

At the lattice scale, several works have explored the mechanical efficiency of sponge-inspired architectures and formulated explicit optimization problems focused on structural performance. Fernandes et al.[16] employed evolutionary optimization (CMA-ES) to explore 2.5D cubic lattice design spaces and showed that the sponge's characteristic checkerboard geometry with double-diagonal reinforcement approaches the theoretical optimum for buckling resistance, achieving approximately 20% higher effective buckling stress compared to conventional diagonally reinforced lattices. Li and Sun[27] extended this analysis using multi-objective optimization (NSGA-

II), identifying alternative sponge-inspired configurations that improved stiffness and strength while reducing redundant material. Fully three-dimensional implementations were investigated by Li et al.[28], who demonstrated that cylindrical sponge-inspired lattices outperform hollow honeycomb structures in compressive strength-to-weight ratio. Vangelatos et al.[19] analyzed the nonlinear buckling behavior of sponge inspired lattices and designed an optimized structure via topology optimization, achieving higher load-carrying capacity with reduced volume compared to a baseline geometry. Sharma and Hiremath[29] extended sponge-inspired concepts to crashworthiness applications, showing through parametric studies that bioinspired thin-walled tubes exhibit significantly higher specific energy absorption at lower peak crushing forces than other bionic tube designs. Chen et al.[20] performed parametric mechanical simulations and showed that sponge-like double-diagonal lattices tend to maximize torsional rigidity while exhibiting weak sensitivity to diagonal spacing, enabling independent control of porosity relevant for fluid transport. Additionally, Du et al.[30] introduced machine-learning-assisted surrogate modeling to optimize simplified sponge-like lattices, achieving improvements of roughly 40% buckling resistance relative to the biological baseline.

In parallel, substantial effort has been devoted to understanding the hydrodynamic role of the E.a. skeleton. Chen et al.[20] studied the influence of the helical ridge system spiraling along the cylindrical lattice and showed that these ridges enhance radial stiffness while affecting fluid transport. Using CFD on sponge-inspired porous, thin-plate representations, they reported improved permeability under laminar conditions (Re ≈ 43), though such models do not capture the full three-dimensional wake dynamics around a cylindrical geometry. Falcucci et al.[21,24] carried out high-performance computing (HPC) flow simulations on a complete skeletal model of E.a., showing that the sponge skeleton reduces hydrodynamic stress and drag forces by up to 49% at Re=2000 compared to solid cylinders. In related work, Fernandes et al.[22] analyzed the flow–structure interaction of ridge-reinforced cylindrical models and demonstrated that the sponge's ridge system suppresses von Kármán vortex shedding[31,32], thereby mitigating resonance-induced vibrations and reducing lift-force oscillations across flow regimes. While these ridges provide only limited contribution to mechanical resistance under compression, they were identified as a key geometric feature governing vortex-shedding control in bioinspired cylindrical structures.

Taken together, prior work has firmly established the outstanding mechanical efficiency and favorable hydrodynamic characteristics of E.a.-inspired architectures, making them attractive templates for engineered systems where structural resilience and fluid control are equally important, such as offshore structures, aerospace components, filtration devices, and biomedical scaffolds. However, existing studies have predominantly treated mechanical and fluid-dynamic performance as separate objectives. Fluidic behavior has not been explicitly optimized, nor has it been systematically coupled with mechanical performance within a unified, large-scale multi-objective design framework. Even studies that addressed both physics typically considered them sequentially or in isolation, with CFD evaluations limited to a small number of representative geometries rather than embedded within automated optimization loops exploring extensive design

spaces. Moreover, experimental validation has largely focused on mechanical metrics, while fluid-dynamic performance has rarely been measured or quantitatively compared with CFD predictions. Consequently, the coupled trade-offs that ultimately govern multifunctional performance in sponge-inspired architectures remain insufficiently characterized. Addressing these challenges requires an integrated framework capable of balancing competing objectives, which is increasingly important given the demand for lightweight structures and efficient fluid management in engineering applications.

We appreciate the reviewer's suggestion of an Ashby-type comparison. While classical Ashby charts are well suited for comparing material classes or bulk cellular architectures, the present study focuses on multiphysics trade-offs (mechanical stability vs. hydrodynamic forces) within a fixed material system and lattice family. Those objectives are strongly geometry-dependent and influenced by boundary conditions such as material selection, global aspect ratio, and the external dimensions of the structure (e.g., lattice diameter and length). Because these quantities do not represent intrinsic material properties in the classical Ashby sense, and because the external dimensions (lattice diameter and length) were intentionally held constant in our optimization study, a traditional Ashby chart or direct comparison to prior designs in our opinion would not yield meaningful or transferable insights. Our framework addresses multiphysics trade-offs that have not been jointly examined in prior Euplectella-inspired studies, making direct design-to-design comparisons with earlier single-physics or descriptive analyses inherently challenging.

This limitation is also reflected in the existing literature cited in our introduction *(Fernandes et al. 2021; Li and Sun 2022; Li et al. 2020; Vangelatos et al. 2023; Chen et al. 2022; Du et al. 2023)*, where most studies compare bioinspired or optimized designs either to baseline configurations within the same optimization framework or to alternative lattice geometries studied in parallel, rather than via universal material-property charts to previous work. (*Only Sharma and Hiremath 2023* have presented an Ashby-style comparison of normalized crashworthiness metrics for different bionic tubes).

We adapted a similar strategy and incorporated quantitative comparisons to baseline geometries wherever feasible, and we significantly expanded these aspects in the revised manuscript. Relevant quotations of the revised manuscript are provided below. Specifically, we benchmark the fluidic response of optimized designs against a solid cylinder of matching length and diameter, and we quantify mechanical improvements by comparing optimized structures to the random Latin Hypercube–sampled designs of similar volume used during MOBO initialization. In addition, we extended the discussion of how our optimized geometries relate to the natural architecture of *Euplectella aspergillum*:

Revised manuscript, Results section (Lines 283 - 295):

"Validation of fluidic performance was performed using two complementary approaches: a CFD baseline simulation of a solid cylinder and SPIV measurements on the fabricated lattice structures. The solid cylinder served as a reference case for validating our CFD formulation, with detailed

comparisons presented in Supplementary Fig. 9. SPIV provided time-resolved velocity fields under quasi-steady flow at a Reynolds number of ~2100, enabling a direct assessment of wake behavior and unsteady loading. As shown in Fig. 5, all optimized designs, regardless of porosity, substantially reduce hydrodynamic loading relative to the solid-cylinder benchmark.

For the reference solid cylinder, the drag coefficient remained close to 1.0, consistent with literature. The optimized designs reduced drag to 0.8–0.85 (Fig. 5a). Lift fluctuations were also suppressed (Fig. 5b): Design A, the most rigid structure, reduced the RMS lift coefficient by 39%, while the most porous, Design I, achieved an 84% reduction. Power spectral density of the lift signal (Fig. 5c) confirmed the attenuation of vortex shedding, with all optimized designs showing reduced spectral peaks near 0.9 Hz, matching the expected Strouhal number of ~0.21."

Revised manuscript, Discussion section (Lines 338 - 366):

"The optimization yields substantial mechanical performance gains relative to baseline geometries, with the critical buckling load increasing on average by ~140% compared to the initial Latin Hypercube samples while maintaining or reducing material usage. Sensitivity analysis (Supplementary Discussion and Fig. 3) provides insights how this improvement is achieved: beam cross-section dimensions H and W and the number of vertical beams N_V emerge as the dominant controls on mechanical performance, consistent with their influence on bending stiffness, moment of inertia, and primary load-bearing pathways. These trends, together with the strong agreement between nonlinear FEA and compression experiments in terms of buckling loads, force–displacement response, and failure locations (Supplementary Fig. 5), confirm that the optimization is guided by physically meaningful structure–property relationships.

Fluid dynamic efficiency is improved in parallel. The optimized lattices reduce drag and suppress unsteady lift relative to a solid-cylinder benchmark while preserving significant load-bearing capacity. In CFD, drag coefficients of the optimized designs fall well below those of the solid cylinder and are consistent with reported values for far more porous sponge structures[21]. As detailed in the results section, unsteady lift forces are strongly attenuated across the design set, and SPIV measurements confirm the CFD predictions. The observed wake stabilization is consistent with prior findings that increasing porosity attenuates turbulence kinetic energy (TKE), elongates shear layers, and suppresses vortex shedding[43–46]. In our case, the time-averaged TKE in the cylinder wake was about 50% higher and peaked roughly one diameter closer to the body than in the optimized designs, while CFD predicted an even more pronounced downstream shift than observed in SPIV, as shown in Supplementary Fig. 16. Our results further show that porosities as low as 5% are sufficient to suppress vortex shedding, a significant reduction compared to the effective porosity of natural E.a. structures. This highlights how targeted geometric refinement through optimization can exceed biological benchmarks for specific functional metrics. While natural sponges may retain higher porosities due to multifunctional biological roles[47–49], our designs isolate and enhance a subset of performance attributes. Prior work on perforated cylinders reported similar wake suppression at porosities around 8%[45], with

similar wake suppression mechanisms[43,46]. Although those studies were typically conducted at higher Reynolds numbers, our results show that optimization can achieve comparable benefits at lower porosities."

The Pareto front serves as a performance envelope, capturing the fundamental trade-offs between mechanical and fluidic performance within the explored design space. Additionally, following the reviewer's suggestion, **we performed a sensitivity analysis**, included in the Supplementary Information which identifies the most influential geometric parameters for each objective. This analysis provides a basis for comparing parameter influence within our geometry-driven, multiphysics problem. We hope that the Reviewer finds this as a valid alternative to the Ashby-type comparison. To **further support reproducibility**, we also provide all investigated geometries from the optimization, together with their geometric parameters, cost-function values, and images, as supplementary downloadable files, enabling readers to explore additional structure–property relationships as desired.

The sensitivity analysis evaluates the influence of each geometric design parameter on both mechanical and fluidic objectives using Pearson correlation (Supplementary Fig. 3a), Spearman correlation (Supplementary Fig. 3b), and standardized regression coefficients (Supplementary Fig. 3c).

The Pearson and Spearman heatmaps (Fig. **a** and Fig. **b**) reveal the pairwise correlations between the geometric design parameters and the two objective functions. Both correlation matrices show a consistent pattern: H (beam cross section height), W (beam cross section width), and N_V (number of vertical beams) exhibit the strongest influence on the mechanical objective, with correlation magnitudes in the range of 0.4–0.85 across the two metrics. This aligns with structural mechanics theory as these parameters directly increase the cross-sectional moment of inertia, stiffness, and available load-bearing paths, which dominate the critical buckling response. The strong negative correlations simply reflect the definition of the mechanical cost function (which decreases for better buckling performance).

In contrast, the fluidic objective shows weaker and more diffuse correlations, with values typically in the range of |0.05–0.27|. This is expected: flow behavior around an open, multiscale lattice is governed by nonlinear flow–structure interactions, vortex shedding, and local pore topology, none of which are controlled by a single geometric variable. Fluidic performance emerges likely from global porosity distribution and the interaction of helical ridges, which explains the broader spread and lower magnitude of correlations. The split in sign across parameters also reflects that drag, lift, and lift fluctuations respond differently to geometric modifications.

The Standardized Regression Coefficient (SRC) box plot (Fig. **c**) confirms these trends. Across repeated bootstrapped fits, H, W, and N_V consistently exhibit the highest regression weights for the mechanical objective, whereas the fluidic objective displays smaller and more scattered coefficients, again indicating a more complex and multi-parameter dependency. Importantly, the

three sensitivity approaches (Pearson, Spearman, SRC) are mutually consistent, strengthening confidence in the observed parameter ranking.

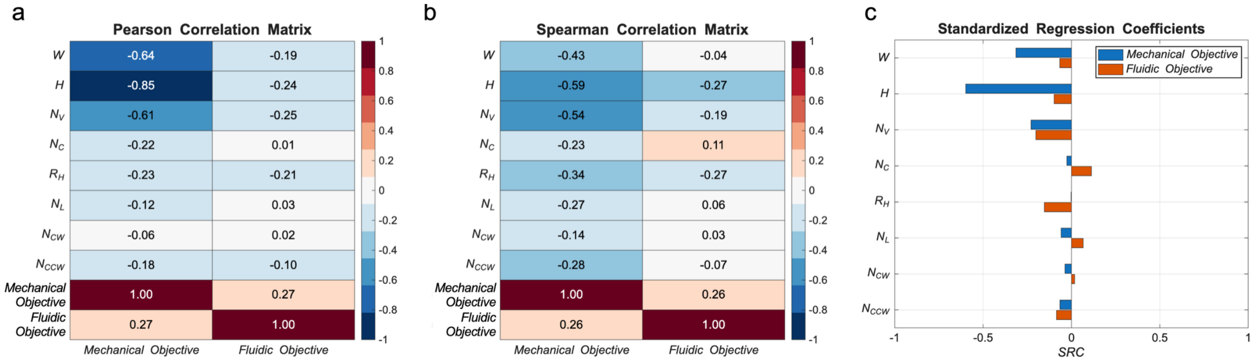

Supplementary Fig. 3: Sensitivity Analysis of MOBO optimization. a Pearson correlation matrix showing linear correlations between geometric parameters and the mechanical and fluidic objectives, with W, H, and N_V having the strongest influence on mechanical performance. **b** Spearman correlation matrix confirming these trends using rank-based, monotonic relationships, with weaker and more diffuse correlations for the fluidic objective. **c** Box plot of standardized regression coefficients, highlighting the same dominant parameters for mechanics and the more distributed sensitivity of the fluidic objective across multiple geometric features.

We note that global sensitivity metrics such as Sobol indices are not feasible for this problem, as they require thousands of uniformly sampled evaluations over the full design space, computationally prohibitive given that each CFD and FEA evaluation is expensive. Moreover, because MOBO adaptively samples regions near the Pareto set, the resulting dataset reflects local sensitivity structure, not uniform global variation. This can make correlations appear more pronounced or more aligned in sign, an inherent property of adaptive sampling in multi-objective optimization.

Despite the largely negative correlations for mechanical performance, simultaneously maximizing all geometric parameters does not yield a Pareto-optimal design. This highlights the non-monotonic and multi-objective nature of the design landscape: enhancing mechanical strength often increases blockage and deteriorates fluidic performance. The moderate cross-correlation between the two objectives (≈0.25–0.27) confirms that they are partially competing but not redundant, validating the use of a multi-objective Bayesian optimization framework.

Overall, the sensitivity analysis reinforces that mechanical performance is governed by classical stiffness-driving parameters, fluidic performance arises from multivariate, shape-sensitive flow phenomena not dominated by any single parameter, and the optimization problem is genuinely multi-objective, supporting the necessity and suitability of the MOBO strategy. We thank the reviewer for the suggestion to add a sensitivity study and believe it adds great value to readers.

Revised manuscript, Results section (Lines 241 - 255):

"To further quantify how geometric parameters influence the optimization landscape, we performed a sensitivity analysis on the MOBO dataset using Pearson correlations, Spearman rank correlations, and standardized regression coefficients, with full results presented in the Supplementary Discussion and Fig. 3. All three metrics consistently identify the beam cross-section dimensions H and W, and the number of vertical members N_V, as the dominant contributors to mechanical performance, with correlation magnitudes in the range of approximately 0.4 – 0.85. This is consistent with structural mechanics theory, as these parameters directly increase bending stiffness, moment of inertia, and load-bearing pathways, thereby governing buckling resistance.

In contrast, the fluidic objective exhibits weaker and more distributed sensitivities across all parameters ($|\rho_i| \lesssim 0.3$), indicating that drag, lift, and vortex shedding arise from multivariate geometric interactions, such as global porosity distribution, rather than a single controlling parameter. The moderate cross-correlation between the two objective functions (≈0.25 – 0.27) confirms that mechanical and fluidic objectives are partially competing but not redundant, reinforcing that the optimization problem is genuinely multi-objective and well-suited for a MOBO framework."

Revised manuscript, Discussion section (Lines 367 - 374):

"The interplay between mechanical and fluidic objectives is further clarified by the Supplementary sensitivity analysis. While mechanical performance is dominated by a few geometric parameters, the fluidic objective exhibits weaker and more distributed sensitivities across all parameters, suggesting that drag, lift, and vortex shedding arise from collective geometric interactions rather than a single controlling variable. The moderate cross-correlation between mechanical and fluidic objectives indicates that they are partially competing but not redundant, reinforcing that the problem is genuinely multi-objective and well suited to a MOBO framework rather than a weighted single-objective optimization."

The detailed explanation provided above has been incorporated into the revised SI (Pages 4 – 6), where the figure, extended interpretation, and methodology notes.

We next address the methodology concerns raised by the reviewer.

The simulations used during MOBO were performed at the highest feasible resolution that still enabled evaluation of hundreds of geometries. We also note that MOBO, chosen for optimization in our study, offers several advantages that are particularly important: it requires fewer expensive function evaluations compared to population-based optimizers, supports efficient and uncertainty-aware exploration of the design space through probabilistic surrogate models, and is well established in the optimization literature for balancing exploration and exploitation (see *Bradford et al. 2018* for further details and benchmarking). These properties make MOBO especially suitable for problems involving costly fluid–structure simulations, where evaluating even a modest number of designs must be done judiciously.

Comparisons to alternative optimization methodologies or massively higher-resolution sweeps are unfortunately not tractable here due to the substantial computational cost. To the best of our knowledge, none of the existing sponge-inspired optimization studies *(Fernandes et al. 2021; Li and Sun 2022; Li et al. 2020; Vangelatos et al. 2023; Chen et al. 2022; Du et al. 2023, Sharma et al. 2023)*, have systematically compared the performance of multiple optimization algorithms in conjunction with high-fidelity simulations, nor have they embedded fully resolved CFD analyses directly within a large-scale multi-objective optimization loop.

Additional methodology concerns are addressed in subsequent responses (see Comment 4 for the discussion of the design sweep, Comments 6 and 7 for mechanical analysis clarifications, and Comment 8 for the VOF formulation).

Relevant References:

- Fernandes, M. C. et al. Mechanically robust lattices inspired by deep-sea glass sponges. Nat. Mater. 20, 237–241 (2021).
- Li, Q.-W. & Sun, B.-H. Optimization of a lattice structure inspired by glass sponge. Bioinspir. Biomim. 18, 016005 (2023).
- Li, L. et al. Optimization design of lightweight structure inspired by glass sponges (Porifera, Hexacinellida) and its mechanical properties. Bioinspir. Biomim. 15, 036006 (2020)
- Vangelatos, Z. et al. A designer's challenge: Unraveling the architected structure of deep sea sponges for lattice mechanical metamaterials. Extreme Mechanics Letters 61, 102013 (2023)
- Chen, H. et al. Lightweight lattice-based skeleton of the sponge Euplectella aspergillum: On the multifunctional design. Journal of the Mechanical Behavior of Biomedical Materials 135, 105448 (2022).
- Du, Z. et al. Artificial intelligence-enhanced bioinspiration: Design of optimized mechanical lattices beyond deep-sea sponges. Extreme Mech. Lett. 62, 102033 (2023).
- Sharma, D. & Hiremath, S. S. Design of Euplectella aspergillum based bionic thin tubes for impact absorbing application under different loading conditions. J. Mater. Res. Technol. 23, 3790–3810 (2023).
- Bradford, E. et al. Efficient multiobjective optimization employing Gaussian processes, spectral sampling and a genetic algorithm. J. Glob. Optim. 71, 407–438 (2018).

1. Introduction, Lines 95–96: “In contrast, MOBO is effective for optimizing costly black-box functions with a limited number of evaluations and can be parallelized on HPC resources.” The authors should explicitly state that MOBO uses a probabilistic surrogate model.

We agree with the Reviewer that the probabilistic nature of MOBO should be stated explicitly in the Introduction. In the revised manuscript, we now clarify that MOBO relies on probabilistic surrogate models, specifically Gaussian process (GP) surrogates, which approximate the black-box objectives and guide the selection of new design points through acquisition functions. We have also expanded the Methods section to briefly summarize the TSEMO algorithm and its use of GP models for multi-objective surrogate construction. Further details about the algorithm can be found in *Bradford et al. 2018*.

Revised text in Introduction (Lines 127 - 131):

“In contrast, MOBO enables efficient optimization of costly black-box functions with a limited number of evaluations[37,38] and can be parallelized on high-performance computing resources. Here, MOBO relies on probabilistic Gaussian-process surrogate models for each objective, allowing uncertainty-aware sampling and sample-efficient exploration of the design space.”

Revised text in Methods (Lines: 430 - 436):

“The optimization process was initialized with 50 Latin Hypercube samples[55]. We then applied the Thompson Sampling Efficient Multi-Objective (TSEMO) algorithm[34] to generate Pareto-optimal solutions, iterating through 22 batches of 20 samples each. TSEMO, as detailed in Bradford et al.[34], constructs independent Gaussian-process surrogate models for the mechanical and fluidic objectives and selects new samples by optimizing a multi-objective acquisition function via Thompson sampling, using the hypervolume quality indicator and NSGA-II to choose a new evaluation point at each iteration.

Relevant References:

- Bradford, E. et al. Efficient multiobjective optimization employing Gaussian processes, spectral sampling and a genetic algorithm. J. Glob. Optim. 71, 407–438 (2018).

2. Introduction, Lines 97–98: "…balancing mechanical resilience and hydrodynamic performance." "Mechanical resilience" is too generic; specify the mechanical property (stiffness, compliance, strain energy density, or toughness).

We agree with the Reviewer. We have revised the text to explicitly state the exact mechanical metric used in the optimization. In the revised manuscript, we now specify that mechanical performance is defined as the critical buckling load normalized by lattice volume, a metric relevant for lightweight cylindrical lattice structures.

Similarly, we clarified the meaning of "hydrodynamic performance" by explicitly referring to the fluid-dynamic quantities minimized in the optimization, namely drag force, lift force, and vortex shedding defined by the standard deviation of lift fluctuations. These definitions are now clearly stated in the Introduction, with additional details and exact cost functions provided in the Methods section.

Revised sentence in manuscript (Lines 132 - 136):

"Using these capabilities, our framework efficiently explores complex design spaces to identify Pareto-optimal solutions inspired by the E.a. skeleton, balancing mechanical performance, defined as the critical buckling load normalized by volume, and hydrodynamic performance, quantified by drag force, lift force, and vortex-shedding behavior. Detailed formulations of both cost functions are provided in the Methods section (Equations 1 and 2)."

Methods (Lines 418 - 429):

Two cost functions were defined to capture the competing objectives of structural strength and fluidic performance. The mechanical cost function F_{mech}, Equation 1:

$$F_{mech} = -\,{F_{Buckling}}/{V_{sponge}} \tag{1}$$

was designed to maximize the lattice's buckling resistance while minimizing material usage, where $F_{Buckling}$ is the standardized critical buckling force obtained from FEA and V_{Sponge} is the lattice volume. The fluid dynamic cost function F_{fluid}, Equation 2:

$$F_{fluid} = \alpha * \overline{F_D} + \beta * \overline{F_L} + \gamma * \sigma_{F_L} \tag{2}$$

combined average drag forces ($\overline{F_D}$), average lift ($\overline{F_L}$) forces, and σ_{F_L} the over time calculated standard deviation of the lift force, a measure for lift force oscillations and vortex shedding. Weighting factors were chosen as $\alpha = \beta = 0.2$ and $\gamma = 0.6$, to emphasize suppression of unsteady lift. The overall optimization problem was therefore formulated as a bi-objective optimization, minimizing F_{mech} and F_{fluid} simultaneously.

3. Figure 1F: The figure is poorly constructed; several applications overlap and are not clearly labeled.

We agree with the Reviewer. In the revised manuscript, we have redesigned Panel **f** to improve clarity and avoid overlapping visual elements. To ensure a more coherent presentation, we now show a single, clearly labeled example application rather than multiple concepts in one panel. In addition, Panels **a** to **e** have been slightly rearranged and refined to follow the conceptual workflow of the paper more clearly, from biological inspiration, to geometric abstraction, to multiphysics simulation, to potential engineering relevance.

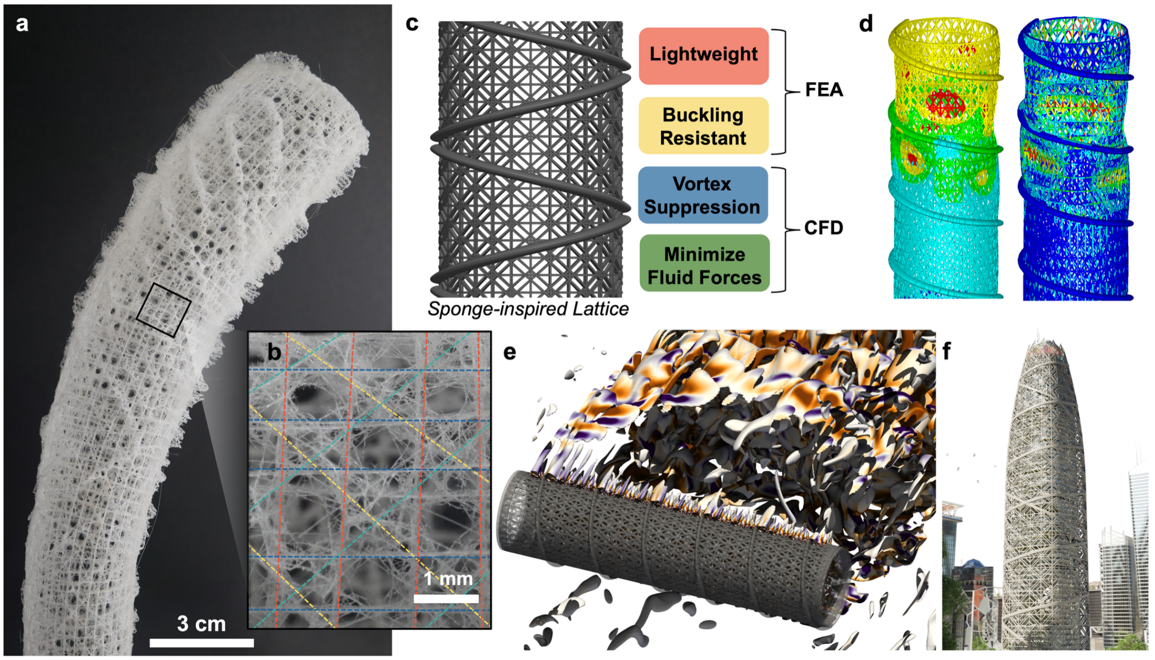

Fig. 1: Biological inspiration, geometric abstraction, and example application of *Euplectella*-inspired lattices. a Photograph of the deep-sea sponge Euplectella aspergillum, whose skeletal architecture exhibits hierarchical organization, diagonal reinforcement, and helical ridges. **b** Magnified view highlighting characteristic geometric motifs within the sponge skeleton. **c** Parametric sponge-inspired lattice model forming the basis of our automated optimization framework. The engineered geometry preserves key features of the sponge's lightweight architecture while enabling evaluation of buckling resistance through FEA and vortex suppression and fluidic forces through CFD. **d** FEA results for an example lattice showing deformation and stress fields under axial compression, used to quantify stiffness and critical buckling load. **e** CFD simulation (incompressible Navier–Stokes with Brinkman penalization) illustrating flow structures and wake behavior through the lattice, used to quantify fluidic forces. **f** Example application demonstrating how such optimized multifunctional lattice architectures could be integrated into lightweight, wind-resistant engineering or architectural systems.

4. Figure 2B (MOBO Flow): The flow in Fig. 2B exposes a major weakness in the optimization framework. The design space was explored using "high-fidelity" simulations with coarse-mesh settings, as stated in the manuscript. The batch generation step relies entirely on the surrogate model trained during the MOBO stage. Therefore, any inaccuracies in the coarse evaluations will directly bias the selection process. Even though the algorithmic framework appears elaborate, the entire optimization outcome is limited by the accuracy of the MOBO surrogate model, which drives all subsequent design decisions. Lines 158–159: "During the MOBO stage, coarser AMR settings were applied…"

We thank the reviewer for raising this point. We agree that the resolution of the exploratory simulations used during the MOBO stage must be clearly justified, and we have revised the manuscript and Supplementary Information accordingly.

Also, while we agree with the Reviewer on the issue of bias, we note that it is standard practice to use coarse resolution (or other reduced order models) for guiding at least the initial stage of evolutionary optimization algorithms applied to expensive simulations, a common issue in problems in fluid mechanics (please see also these references)

- J.W. Bandler, R.M. Biernacki, S.H. Chen, P.A. Grobelny, and R.H. Hemmers, "Space mapping technique for electromagnetic optimization," IEEE Trans. Microwave Theory Tech., vol. 42, no. 12, pp. 2536-2544, Dec. 1994.
- Wang, H., Jin, Y. and Doherty, J., 2017. A generic test suite for evolutionary multifidelity optimization. *IEEE Transactions on Evolutionary Computation*, *22*(6), pp.836-850.
- Van Rees, Wim M., Mattia Gazzola, and Petros Koumoutsakos. "Optimal shapes for anguilliform swimmers at intermediate Reynolds numbers." *Journal of Fluid Mechanics* 722 (2013): R3.

The simulations used during the MOBO stage employed moderate-resolution, flow-resolved AMR settings, the highest resolution that remained computationally feasible for evaluating hundreds of designs. This two-fidelity strategy, in which the optimization loop uses exploratory-resolution simulations while all final quantitative reporting relies on higher-resolution simulations, is widely adopted in design optimization, where performing fully resolved evaluations for every MOBO iteration is computationally prohibitive (*Leifsson and Koziel 2010; Forrester et al. 2008; Kandasamy et al. 2017*).

To demonstrate that the exploratory-resolution simulations remain sufficiently accurate to guide surrogate-model construction, we now include an additional performance-space comparison (Supplementary Fig. 6) in the SI. This figure compares the optimization-stage cost-function values to the corresponding high-resolution evaluations for the four Pareto-optimal structures (A, D, H, I). The final evaluations preserve the same ordering and relative spacing, and deviations between resolutions are small. This confirms that the exploratory AMR settings capture the correct global

performance trends, drag, lift, and vortex-shedding intensity, that the MOBO surrogate model relies upon.

We further note that, even at exploratory resolution, the baseline solid-cylinder forces (drag and lift) match established literature values, demonstrating that the essential flow physics are resolved. Importantly, none of the reported quantitative results, comparisons to experiments, or visualizations rely on exploratory-resolution data. All final evaluations used higher-resolution AMR settings, and only these high-resolution results inform the performance assessment and SPIV validation.

To improve clarity, we have updated the manuscript to remove the term "coarse mesh" and replaced it with "moderate-resolution, flow-resolved simulations," and we have expanded the Methods section to more clearly describe the two-fidelity simulation strategy.

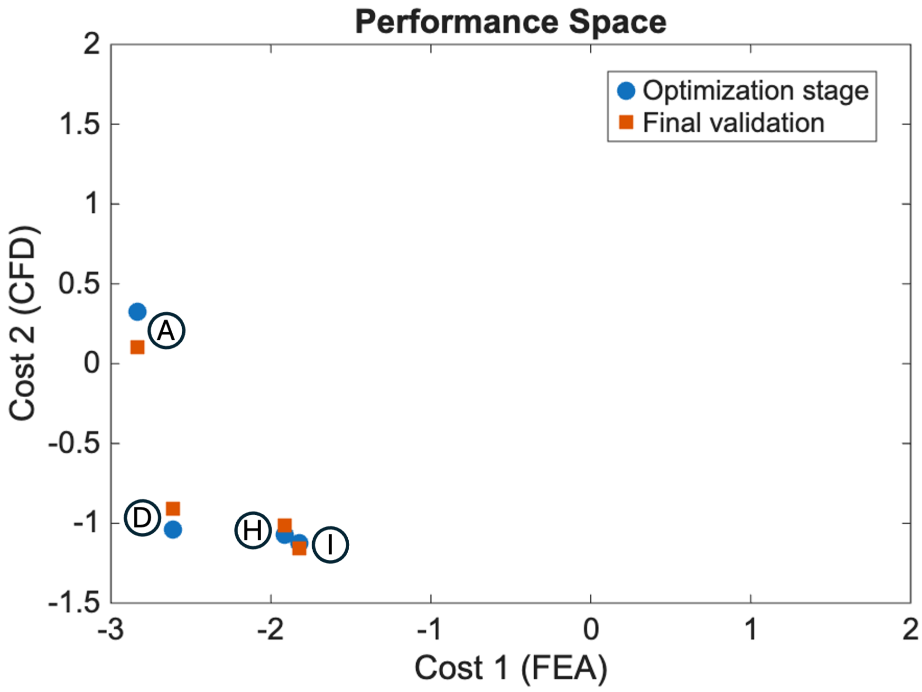

Supplementary Fig. 6: Comparison of optimization-stage and final high-resolution cost-function evaluations. Performance-space comparison for the four selected Pareto-optimal designs (A, D, H, I). Blue circles show the results obtained during the MOBO optimization stage using exploratory-resolution AMR settings, and orange squares show the corresponding final high-resolution evaluations used for all reported quantitative results and experimental validation. The ordering of designs is preserved, and deviations between exploratory and high-resolution evaluations are small, confirming that the lower-cost exploratory simulations reliably capture the global performance trends needed for MOBO-driven design selection.

Revise part of manuscript (Lines 186 - 191):

"During the MOBO stage, moderate-resolution, flow-resolved AMR settings were employed to efficiently explore the design space across hundreds of candidate geometries. For all final Pareto-optimal designs, higher-resolution simulations were subsequently performed involving grids at a resolution that enabled Direct Numerical Simulations (DNS). The validity and rationale of this two-fidelity simulation strategy are detailed in the Methods section and Supplementary Discussion."

Methods (Lines 510 - 518):

"The domain size was L= 12.5D, where D is the diameter of the structure, and the mesh cell size was adaptively refined, ranging from $L/2^8$ to $L/2^{11}$ during the optimization and from $L/2^{10}$ to $L/2^{13}$ for the refined simulations. The finest mesh resolution, $L/2^{13} \approx 0.0015D$ was approximately five times smaller than the smallest structural features and pore sizes, ensuring accurate geometric representation and flow resolution. Coarser cells were retained in low-gradient areas to reduce cost. This two-fidelity strategy enabled efficient exploration of hundreds of candidate geometries during optimization while ensuring fully resolved flow fields for all reported quantitative results. Additional details and validation of the two-fidelity workflow are provided in the Supplementary Discussion and Supplementary Fig. 6."

To avoid unnecessary repetition, we do not restate the full text from the Supplementary Information here (SI, Pages 9 – 10, Supplementary Fig. 6). The detailed explanation in this rebuttal is identical in content to the material that has been incorporated into the revised SI.

Relevant References:

- Leifsson, L. & Koziel, S. Multi-fidelity design optimization of transonic airfoils using shape-preserving response prediction. Procedia Comput. Sci. 1, 1311–1320 (2010).
- Forrester, A. I. J. et al. Engineering Design via Surrogate Modelling: A Practical Guide. (John Wiley, Chichester, 2008). doi:10.1002/9780470770801
- Kandasamy, K. et al. Multi-fidelity bayesian optimisation with continuous approximations. in International conference on machine learning 1799–1808 (PMLR, 2017)
- Kern, S., Hansen, N. and Koumoutsakos, P., 2006, September. Local meta-models for optimization using evolution strategies. In *International Conference on Parallel Problem Solving from Nature* (pp. 939-948). Berlin, Heidelberg: Springer Berlin Heidelberg.

5. Lines 177–178: "The optimization produced a design space mapping the trade-offs between mechanical strength and fluidic efficiency." It is unclear what the mechanical cost function represents. The authors refer to "strength," but this needs clarification. The fluidic cost function is described as "fluidic efficiency," which is vague. Define this term precisely.

   We agree and have revised the manuscript accordingly. As previously also discussed in our response to Comment 2, we now explicitly state the exact physical quantities optimized in this study. The mechanical objective is defined as the critical buckling load normalized by the lattice volume, commonly used for lightweight cylindrical structures. The fluidic objective is defined through the reduction of drag force, lift force, and the standard deviation of lift fluctuations, the latter serving as a quantitative measure of vortex shedding intensity.

   We have updated both the Introduction and the relevant Results section to reflect these definitions. We also refer readers to the Methods section, where the full cost-function formulations (Eqs. 1–2) are provided as discussed in Comment 2.

   Revised text in Introduction (Lines 132 - 136):

   "Using these capabilities, our framework efficiently explores complex design spaces to identify Pareto-optimal solutions inspired by the E.a. skeleton, balancing mechanical performance, defined as the critical buckling load normalized by volume, and hydrodynamic performance, quantified by drag force, lift force, and vortex-shedding behavior. Detailed formulations of both cost functions are provided in the Methods section (Eqs. 1 and 2)."

   Revised text in Results (Lines 220 - 223):

   "The optimization produced a design space mapping the trade-offs between mechanical performance, defined as the critical buckling load normalized by volume, and fluidic performance, quantified by reductions in drag force, lift force, and vortex-shedding intensity (Fig. 3a; see Methods, Eqs. 1 and 2, for full cost-function definitions). Pareto-optimal designs are shown in red."

6. Lines 389–391: "Linear buckling analysis (Analysis type: BUCKLE) to obtain the critical buckling force." Stating "Analysis type: BUCKLE" is not a scientific description of the method. Specify whether the buckling was captured using an eigenvalue-based approach or a transient nonlinear analysis.

   We agree with the Reviewer. In the revised manuscript, we now explicitly state that the linear buckling study was performed using a linear perturbation eigenvalue buckling

analysis, which computes the critical load factor and the corresponding first buckling mode.

We also clarify that this eigenvalue-based analysis was used solely to predict the onset of instability during optimization stage, while geometrically nonlinear buckling simulations were subsequently performed for the Pareto-optimal designs to capture mode evolution and post-buckling response as discussed in Comment 7.

Revised Methods (Lines: 470 - 474):

Two types of structural simulations were performed. First, a linear static analysis was used to evaluate the elastic stiffness of each lattice. Second, the critical buckling load was obtained using a linear perturbation eigenvalue buckling analysis, which computes the first buckling mode and its associated load factor under a uniform compressive pressure applied on the top surface along the z-axis.

7. Lines 395: "…deformations (NLGEOM), and post-buckling behavior…" NLGEOM enables large deformation effects but does not simulate imperfections. Explain how imperfections were introduced and how they relate to NLGEOM.

We thank the Reviewer for pointing that out. NLGEOM alone does not introduce geometric imperfections. In the revised manuscript, we now explicitly clarify how imperfections were incorporated. Specifically, initial imperfections were introduced by scaling the first buckling eigenmode computed from the linear eigenvalue buckling analysis, using the UPGEOM command in Ansys. This eigenmode-based imperfection seeding is a widely established approach for triggering realistic instability in nonlinear FEA, as documented in Ansys tutorials and literature (*Teng et al. 2004; He et al. 2018; Pastor et al. 2013*). Applying this mode shape as a small geometric imperfection ensures that the nonlinear simulation captures the physically relevant bifurcation path.

These clarifications have been incorporated into the revised Methods (Lines: 474 – 482):

"For the Pareto-optimal designs, we additionally performed geometrically nonlinear buckling simulations to capture large-deformation behavior and post-buckling response. Initial geometric imperfections were introduced by scaling the first buckling eigenmode obtained from the linear eigenvalue buckling analysis. This eigenmode-based imperfection approach perturbs the geometry along the most critical buckling mode and is widely used to trigger physically realistic instability in nonlinear FEA[58,59]. An imperfection amplitude of 1% of the normalized eigenmode displacement field, scaled relative to the lattice diameter, was applied, providing a small perturbation sufficient to initiate the dominant buckling path without artificially reducing the predicted load capacity."

Relevant References:

- Teng, J. G., et al. Buckling of Thin Metal Shells. (Spon Press, London, 2004)
- He, S. et al. Investigation on Simulation Methods of Initial Geometric Imperfection Distribution in Elasto-plastic Stability Analysis of Single-layer Reticulated Shells. KSCE J. Civ. Eng. 22, 1193–1202 (2018).
- Pastor, M. M. et al. Residual stresses and initial imperfections in non-linear analysis. Eng. Struct. 46, 493–507 (2013)

8. Lines 408: "Using the Volume of Fluid (VOF) method…" The authors must justify the use of VOF, which is intended for multiphase or immiscible fluid flows. If the flow is single-phase, VOF adds unnecessary complexity.

We agree with the Reviewer that VOF is usually deployed in multiphase flow simulations. **We have made a mistake in our statement and we have corrected it in the revised manuscript.** More specifically: We deployed the software Basilisk which allows for simulations using Adapative Mesh Refinement (AMR). Basilisk includes a powerful Volume-of-Fluid (VOF) capability and much of the literature on the code focuses on multiphase VOF applications. However, **in our work no interface was tracked and no VOF formulation was used**. **We employ instead Basilisk's incompressible Navier–Stokes solver, second-order, cell-centered finite-volume discretization (centered differences on an adaptive octree mesh).** The solid geometry is imposed via Brinkman penalization, which enforces no-slip boundary conditions through a resistive body-force term inside the solid region.

Accordingly, the revised manuscript text now accurately describes the solver configuration and no longer mentions VOF. The above-mentioned lines in the Reviewers comment have been updated as follows (Lines 493 - 497):

"All simulations were performed using the open-source solver Basilisk (Popinet, 2009), which solves the incompressible Navier–Stokes equations using a second-order, cell-centered finite-volume formulation on an adaptive octree grid (Popinet, 2003; Lagrée et al., 2011). Solid regions were imposed through Brinkman penalization, implemented as a resistive body-force term following the formulation of Angot et al. (1999),…"

Relevant references:

- Basilisk documentation: https://basilisk.fr/src/navier-stokes/centered.h
- Popinet, S. An accurate adaptive solver for surface-tension-driven interfacial flows. Journal of Computational Physics 228, 5838–5866 (2009).
- Popinet, S. Gerris: a tree-based adaptive solver for the incompressible Euler equations in complex geometries. J. Comput. Phys. 190, 572–600 (2003).

- Lagrée, P.-Y. et al. The granular column collapse as a continuum: validity of a two-dimensional Navier–Stokes model with a μ(I)-rheology. J. Fluid Mech. 686, 378–408 (2011).
- Angot, P., et al. A penalization method to take into account obstacles in incompressible viscous flows. Numer. Math. 81, 497–520 (1999).

9. Lines 449–450: "…strain rate of 0.1 s^{-1} until failure, indicated by buckling." Clarify whether this strain rate corresponds to a displacement rate that ensures quasi-static conditions. Buckling loads can depend strongly on the applied strain rate.

We thank the Reviewer for noting this inconsistency. **The value reported in the manuscript was incorrect.** The experiments were performed at a displacement rate (not strain rate) of 0.1 mm s^{-1} on specimens of 100 mm length. This corresponds to an engineering strain rate of 10^{-3} s^{-1}, not 0.1 s^{-1}. This correction has been made in the revised manuscript.

The strain rate of 10^{-3} s^{-1} is well within the quasi-static regime: inertial effects are negligible, and the buckling response is essentially rate-independent at rates $\leq 10^{-2}$ s^{-1}, as documented in *Gibson & Ashby 2001* and consistent with ISO 844:2021 for quasi-static compression of cellular polymeric materials. The supplementary experimental videos of the compression experiments provided in the also clearly show slow, quasi-static deformation.

The Method section has been updated accordingly (Lines: 538 - 546):

"Mechanical tests were performed to validate the FEA predictions. Compression experiments were carried out on an Instron 5944 universal testing machine (Instron Corporation, Norwood, MA) using a 2 kN load cell (Instron 2580-2KN). Samples were compressed under displacement control at a rate of 0.1 mm s^{-1}, corresponding to an engineering strain rate of 10^{-3} s^{-1} for the 100 mm-tall specimens. This rate lies well within the quasi-static regime, ensuring a strain-rate–independent buckling response. Tests were recorded at 60 fps to capture the location and progression of failure, and load–displacement data were sampled every 0.02 s to quantify stress–strain behavior and buckling loads with high precision. For each design, four samples were tested to confirm repeatability."

Relevant References:

- Gibson, L. J. & Ashby, M. F. Cellular Solids: Structure and Properties. (Cambridge Univ. Press, Cambridge, 2001)
- ISO 844:2021: Rigid cellular plastics - Determination of compression properties

<u>Reviewer #2</u>

The paper is relevant to the Nature Communications journal. Originality of the work is derived from the multiphysics approach to investigating the Venus flower basket sponge. Technical accuracy and completeness are excellent. References to pertinent work in the field are comprehensive. A proper combination of comprehensiveness and conciseness is achieved well. The quality of presentation is also excellent.

• Note the typo: "sies"

• You could consider including some additional details for the FEA analysis, such as meshing strategies (there was no image of the mesh) and an accompanying mesh convergence study.

Conclusion: This is an extremely well-written paper both in its clarity and its attention to detail. I therefore recommend publication in its current form.

We thank the reviewer for the positive assessment of our work and for the constructive suggestions. We appreciate the reviewer's recognition of the manuscript's originality, clarity, and technical completeness, and we are grateful for the recommendation for publication.

The typo ("sies") has been corrected to "sizes" in the revised manuscript. In addition, we have incorporated the reviewer's suggestion to provide more detail on the finite element meshing strategy.

Specifically, the Methods section has been expanded to clarify the element formulation, the characteristic element length, and the meshing approach used for the lattice architecture. The revised text now reads:

<u>Methods (Lines: 459 – 467):</u>

<u>*"All lattice geometries were discretized using Timoshenko beam elements, selected for their ability to capture bending and shear deformation in slender load-bearing members. A maximum element length of 1 mm was adopted for all optimization and validation simulations. This value was determined through a mesh-refinement study in which the element length was progressively reduced until the computed critical buckling load changed by less than 3% compared to the highest-resolution mesh (0.125 mm). Thus, the selected mesh resolution provides an appropriate balance between computational efficiency and numerical accuracy during the multi-objective optimization. A representative mesh and the corresponding convergence curve are presented in Supplementary Fig. 4."*</u>

Additionally, we added a representative mesh visualization to the Supplementary Information (Supplementary Fig. 4) illustrating the discretization strategy used for the *Euplectella*-inspired lattices, along with a mesh-convergence study. Before conducting any optimization runs, we performed a dedicated mesh refinement analysis to determine an appropriate maximum element size for all subsequent simulations. The mesh was progressively refined by decreasing the

maximum beam element length, and the corresponding critical buckling load was computed at each refinement level using linear eigenvalue buckling analysis. The predicted buckling loads rapidly approached a stable value as the mesh was refined, and the deviation between the 1 mm mesh and the finest-resolution mesh (0.125 mm, approximately six times more elements) was found to be less than 3%. Based on this convergence behavior, we selected a maximum element length of 1 mm for all optimization and final validation simulations, as this level of discretization provides sufficient accuracy while maintaining a tractable computational cost.

Supplementary Information (Pages 6 – 7, Supplementary Fig. 4):

"All lattice geometries were discretized using Timoshenko beam elements (ANSYS BEAM188), which accurately capture both bending and shear deformation in high–slenderness-ratio members. A maximum element length of 1 mm was applied across all beam segments to ensure consistent discretization, though the geometric intersections that form structural nodes naturally result in many segments, and therefore finite elements, being shorter than the nominal maximum. Supplementary Fig. 4a presents a representative mesh with several zoomed-in views illustrating the nodal layout and segmentation. To verify mesh adequacy, we conducted a mesh-convergence study by progressively reducing the maximum element length and computing the associated critical buckling loads. The resulting convergence curve (Supplementary Fig. 4b) shows that the buckling load asymptotically approaches a stable value as the mesh is refined. A maximum element size of 1 mm was therefore selected for all simulations throughout the optimization, as it achieves <3% deviation from the finest-resolution mesh while significantly reducing computational demands."

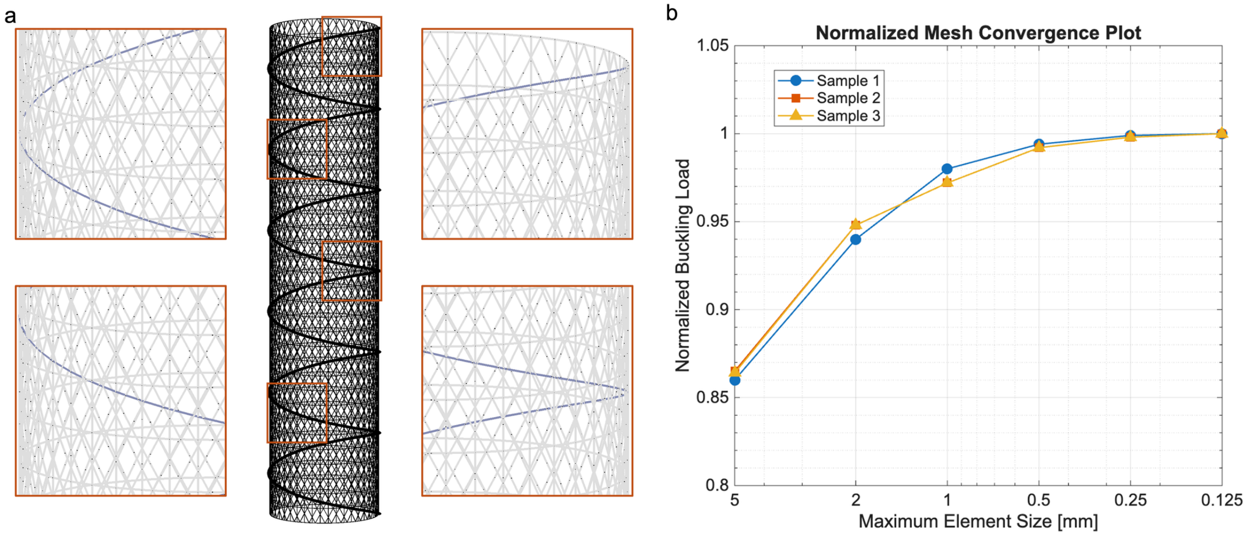

Supplementary Fig. 4: Mesh discretization and FEA convergence study for lattice buckling analysis. **a** Representative finite-element discretization of the Euplectella-inspired lattice structure with multiple detailed zoomed-in views, shown as line geometry with nodal points indicated as black dots. A maximum element length of 1 mm was applied along all beam segments to ensure consistent discretization; however,

due to geometric intersections that form structural nodes, many beam segments, and thus finite elements, are shorter than this nominal maximum. For visualization clarity, elements are not explicitly shown. **b** Mesh-convergence plot of the normalized critical buckling load, obtained from linear eigenvalue buckling analyses for three representative designs. The mesh was progressively refined by decreasing the maximum element length from 5 mm down to 0.125 mm, and the predicted buckling loads were normalized by the value at the finest resolution. The results show that the buckling load approaches a stable value as the mesh is refined. A 1 mm maximum element length was selected for all simulations, yielding deviations below 3% relative to the finest mesh while substantially reducing computational cost.
